# Quorum Sensing Inhibition by Sponge-Associated *Bacillus* Species: Suppressing *Pseudomonas aeruginosa* Virulence Factors

**DOI:** 10.3390/antibiotics14101035

**Published:** 2025-10-16

**Authors:** Carrie Shelouise Jacobs, Ryan Naicker, Hafizah Yousuf Chenia

**Affiliations:** 1 Discipline of Microbiology (Westville Campus), School of Life Sciences, University of KwaZulu-Natal, Private Bag X54001, Durban 4000, South Africa213550897@stu.ukzn.ac.za (R.N.); 2 Department of Microbiology, Stellenbosch University, Stellenbosch Campus, Private Bag X1, Stellenbosch 7599, South Africa

**Keywords:** sponge-associated bacteria, *Bacillus*, quorum sensing inhibition, *Pseudomonas aeruginosa*, anti-virulence, marine natural products

## Abstract

**Background/Objectives**: The growing threat of antimicrobial resistance has intensified the search for alternative therapeutic approaches. Quorum sensing (QS) inhibition, which disrupts bacterial communication and virulence, represents a promising approach to mitigating infection. Given the complexity of the sponge holobiont, sponge-associated microorganisms may demonstrate QS inhibitory properties and serve as potential sources of novel anti-virulence agents. This study aimed to investigate the QS inhibitory potential of sponge-associated *Bacillus* species against *Pseudomonas aeruginosa*, a multidrug-resistant pathogen that relies on QS for virulence regulation. **Methods**: Ninety-eight bacterial isolates were obtained from seven intertidal South African sponges. Biosensor-based sandwich assays using *Chromobacterium violaceum* identified 15 isolates with putative QS inhibition (QSI) activity, including five classified as *Bacillus* species via 16S rRNA gene sequencing. Crude extracts from these isolates, cultivated in medium Mannitol (Mann) and medium 5294, were screened for their ability to inhibit QS-regulated virulence factors in *P. aeruginosa*. **Results**: Extracts, particularly from medium 5294, exhibited significant QSI activity without cytotoxic effects. The five most potent extracts, i.e., *Bacillus mobilis* SP2-AB7 (5294), *Bacillus wiedmannii* SP5-AB7 (Mann), *B. mobilis* SP2-AB7 (Mann), and *Bacillus cereus* SP1-AB4 (Mann and 5294), inhibited both Las- and Rhl-regulated virulence factors, including pyocyanin, pyoverdine, elastase, protease, rhamnolipid production, motility, and initial adhesion, achieving inhibition rates of up to 93% (*p* < 0.05). Molecular analysis confirmed the presence of the *aiiA* lactonase gene in key isolates, while GC-MS and FTIR profiling revealed medium-specific differences in metabolite production. **Conclusions**: Sponge-associated *Bacillus* species from KwaZulu-Natal exhibit robust QSI activity against *P. aeruginosa*, highlighting their potential as sources of alternative anti-virulence agents. Further characterization and in vivo validation are needed to assess their therapeutic application in combatting resistant infections.

## 1. Introduction

*Pseudomonas aeruginosa* is a highly adaptable Gram-negative opportunistic pathogen that is responsible for 10–15% of nosocomial infections, including ventilator-associated pneumonia, urinary tract infections, and bloodstream infections [1,2]. Although it rarely affects healthy individuals, *P. aeruginosa* is a leading cause of hospital-acquired infections. It poses a significant threat to cystic fibrosis patients, burn victims, and immunocompromised individuals due to its adaptability, invasiveness, and extensive virulence factor production [1,2]. It can cause a wide range of infections, from localized and subacute to systemic and life-threatening. The pathogenicity of *P. aeruginosa* is largely attributed to its ability to produce an extensive arsenal of quorum sensing (QS)-regulated virulence traits, including biofilm formation, toxin production, motility, and the secretion of virulence factors such as pyocyanin, pyoverdine, elastase, and protease [3,4]. Flagella and pili facilitate tissue adhesion and motility via swarming and twitching mechanisms, while secreted exoenzymes, exotoxins, and effector proteins contribute to host tissue damage and immune evasion [3,4]. The bacterium also produces the redox-active pigment pyocyanin, which disrupts host oxidative stress responses, and siderophores such as pyoverdine and pyochelin to scavenge iron from host proteins (transferrin and lactoferrin), further enhancing its survival and virulence. In chronic infections, biofilm formation increases resistance to antimicrobials and host immune defences, making treatment particularly challenging. The impressive arsenal of secreted macromolecules and the secondary metabolite “toolbox” of virulence factors makes *P. aeruginosa* a formidable opportunistic pathogen [3,4].

The QS network in *P. aeruginosa* is primarily controlled by four interconnected systems: Las, Rhl, PQS, and IQS. Each system is autoregulatory and modulates the activity of the others, ensuring precise control of virulence expression [3]. The Las system plays a dominant role, regulating QS hierarchy. The *lasI* gene produces N-(3-oxododecanoyl)-L-homoserine lactone (3-oxo-C12-HSL), which activates the LasR regulator. This, in turn, controls the expression of *lasI* and downstream genes involved in elastase (*lasB*), alkaline protease (*apr*), exotoxin A (*toxA*), lipase, and the Rhl system (*rhlR*). The Rhl system, governed by the *rhlI* gene, produces N-butanoyl-L-homoserine lactone (C4-HSL), which activates the *rhlR* regulator, controlling the expression of genes involved in rhamnolipid biosynthesis (*rhlAB*), elastase (*lasA* and *lasB*), phenazine and pyocyanin production, and cyanide synthesis [5,6]. The *las* system positively controls both *rhl* and *pqs* system genes that code for QS signalling molecule receptors (*rhlR* and *pqsR*) and synthase genes (*rhlI* and *pqsH*). While some target genes are specifically regulated by *las* and others by *rhl*, some, however, require both QS systems for full activation [3]. The PQS system, which produces 2-alkyl-4-quinolones (4,2-heptyl-3-hydroxy-4(1*H*)-quinolone, PQS and 3,2-heptyl-4-hydroxyquinoline, HHQ), acts as a QS signal mediator between the Las and Rhl systems. It promotes biofilm formation, swarming, and twitching motility while also inducing the expression of virulence factors, including pyocyanin, rhamnolipids, lectins, and elastase [6]. The IQS system is typically controlled by Las but is activated under phosphate starvation, further modulating multiple QS-dependent genes and virulence factors during infection [7]. The hierarchical regulation of these systems allows *P. aeruginosa* to fine-tune virulence factor expression and antimicrobial resistance in response to environmental and host conditions [5,8]. Beyond its virulence, *P. aeruginosa* exhibits formidable resistance to a broad spectrum of antimicrobial agents through intrinsic (e.g., efflux pumps, chromosomally encoded β-lactamases), acquired (e.g., horizontal gene transfer of resistance genes and/or mutation in target genes), and adaptive mechanisms (e.g., stress-mediated responses, sub-inhibitory antibiotic exposure, and environmental factors) [3]. This adaptability enables resistance to multiple antibiotic classes, making *P. aeruginosa* infections increasingly difficult to treat. The World Health Organization has designated carbapenem-resistant *P. aeruginosa* as a “critical priority pathogen” requiring urgent development of alternative treatment strategies [9,10]. Given its reliance on QS for virulence regulation and its association with multidrug resistance, one such strategy involves QS inhibition (QSI), which disarms pathogens without killing them, thereby reducing the selective pressure for resistance.

Marine microbes hold significant potential for biotechnology and drug discovery due to their diverse chemical structures, underexplored bioactive compounds, and adaptation to the diluted marine environment [11,12,13]. Despite this promise, less than 1% of potential natural products from marine microorganisms have been screened, highlighting the vast untapped resource within marine biodiversity [11,13]. Sponges (*phylum Porifera*) are among the richest sources of marine-derived bioactive compounds. As sessile filter feeders, sponges produce a diverse array of secondary metabolites, including terpenes, polyketide-peptide hybrids, and carbohydrate-based molecules, many of which have demonstrated antimicrobial potential [14,15]. Increasing evidence suggests that sponges also possess QS inhibitory activity, interfering with bacterial communication systems regulating virulence and biofilm formation [16,17,18,19,20,21,22,23,24,25,26,27,28]. Sponges maintain highly specific and diverse microbial symbiont communities, which can constitute up to 50% of their biomass. These symbionts include heterotrophic bacteria, cyanobacteria, archaea, fungi, yeast, dinoflagellates, and even viruses. The symbiotic relationship is largely driven by chemical defence, where microbial symbionts produce secondary metabolites to deter predation and fouling, as well as nutrient cycling and metabolic waste recycling [14,15,29,30]. Many of the bioactive compounds originally attributed to sponges are now recognized as being synthesized by their associated bacteria [31,32].

Among the >35 sponge-associated bacterial genera known to produce antimicrobial compounds, *Streptomyces*, *Pseudovibrio*, and *Bacillus* are the most prominent [15,29,30,31]. Notably, between 5 and 15% of the *Bacillus* genome is dedicated to secondary metabolite production, making these bacteria strong candidates for drug discovery. Marine *Bacillus* species, including those from sponges, produce a wide variety of structurally diverse bioactive compounds, including lipopeptides, macrolactones, fatty acids, polyketides, and isocoumarins. These metabolites have demonstrated antimicrobial, anti-cancer, and anti-algal activities [30,31,33,34,35]. Beyond antimicrobial properties, *Bacillus* species are also known for their ability to interfere with bacterial QS systems, either by producing QS inhibitors or by degrading QS signals through quorum quenching (QQ) enzymes [36]. Despite extensive research into the antimicrobial potential of sponge-associated *Bacillus* species [29,31,33,34], relatively few studies have explored their QSI activity [21,37,38,39,40,41]. *Bacillus* spp. can inhibit QS by two main mechanisms: enzymatic degradation of QS signalling molecules via acyl-homoserine lactone (AHL) lactonases (quorum quenching; QQ) and interference with signal reception via small-molecule inhibitors. These traits make *Bacillus* species attractive candidates for anti-virulence drug discovery, especially in the face of multidrug-resistant pathogens like *P. aeruginosa* [7]. This study investigates the QSI potential of five *Bacillus* species isolated from South African marine sponges. This study specifically evaluated their ability to disrupt QS-regulated virulence phenotypes in *P. aeruginosa*, with the goal of identifying promising candidates for alternative, resistance-sparing therapeutic development.

## 2. Results

### 2.1. Identification of QS Inhibitory Sponge-Associated Bacteria Using Biosensor Sandwich Assays

**Figure 1:**
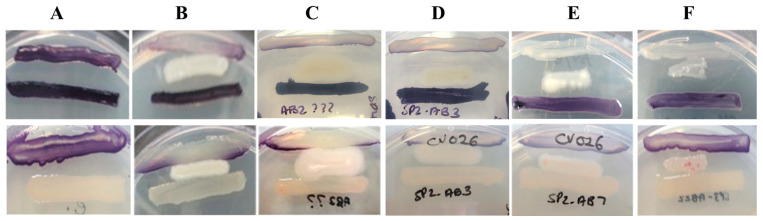
Sandwich assay screening of quorum sensing inhibitory (QSI) potential of 98 sponge-associated bacteria. All isolates were screened for QSI activity using *Chromobacterium violaceum* biosensors. Upper frames indicate *C. violaceum* VIR07 and its long-chain AHL over-producer strain ATCC 12472, while the corresponding lower frames indicate *C. violaceum* CV026 and its corresponding short-chain AHL over-producer ATCC 31532. Controls included (**A**) biosensor control (no test organism streaked between biosensor and its corresponding over-producer) and (**B**) QSI-positive control, *B. cereus* ATCC 14579. Inhibition of pigment production or QSI activity was rated 0–4, whereby 4 was a near total loss of pigment as compared to the control *Bacillus cereus* ATCC 14579 and 0 was complete activation of purple violacein production as observed with biosensor control. (**C**) *Bacillus thuringiensis* SP-AB2 and (**D**) *Streptomyces* sp. SP2-AB3 were rated 4 for both long-chain (top) and short-chain (bottom) AHL inhibition. (**E**) *Bacillus mobilis* SP2-AB7 and (**F**) *Streptomyces* sp. SP3-AB22 were rated as +4 for long-chain and 3 and 1 for short-chain QSI, respectively.

Following the biosensor sandwich assay screening of 98 sponge-associated bacteria, results were compared to that of the QSI-positive control *B. cereus* ATCC 14579 and assigned a QSI rating on a scale from 0 to 4 (Figure 1). Isolates rated 4 exhibited QSI activity equal or greater than that of the *B. cereus* ATCC 14579 control. Among the screened isolates, 88% (86/98) effectively inhibited short-chain signal transduction receiving a rating of 3 or 4. In contrast, only 17% (17/98) demonstrated comparable inhibition for long-chain signals. Among these 17 isolates, the majority (*n* = 16) exhibited broad-spectrum activity, inhibiting both short- and long-chain QS of *C. violaceum*. Based on these findings, 15 isolates were selected for further study (Table 1).

### 2.2. 16S rRNA Gene Identification of Selected QS Inhibitory Isolates

Following 16S rRNA gene amplification and DNA sequencing, 33% (5/15) of the selected sponge-associated bacterial isolates (Supplementary Figure S2) were identified as members of the genus *Bacillus.* Of these, four were further classified within the *B. cereus sensu lato* group (Table 2).

### 2.3. Amplification and Sequencing of the aiiA Lactonase Gene

A 756 bp amplicon was obtained from *B. cereus* ATCC 14579 (MW328524.1) and 60% (3/15) of the sponge-associated *Bacillus* species isolates: *B. thuringiensis* SP-AB2 (MW328525.1), *B. cereus* SP1-AB4 (MW328526.1) and *B. mobilis* SP2-AB7 (MW328527.1). These isolates exhibited >98% DNA sequence similarity to *aiiA* lactonase genes from corresponding *Bacillus* species isolates deposited in Genbank.

### 2.4. Characterization of Sponge-Associated Bacillus Species Extracts

Based on QSI screening (Table 1) and molecular identification (Table 2), five *Bacillus* species isolates were selected for shake flask fermentations using medium Mannitol and medium 5294, followed by ethyl acetate extraction. The 10 resulting extracts were then analyzed using FTIR and GC-MS.

#### 2.4.1. Fourier Transform Infrared Analysis

FTIR analysis of extracts from the five *Bacillus* species cultured in medium Mannitol (Supplementary Table S1) and medium 5294 (Supplementary Table S2) revealed both shared and medium-specific functional groups, underscoring the influence of growth media on bacterial metabolic profiles. In medium Mannitol extracts, O-H stretching vibrations (3200–3550 cm^−1^) indicated the presence of alcohols, while C=C stretching (1626–1662 cm^−1^) and C-H bending (910–990 cm^−1^ and 665–730 cm^−1^) confirmed alkenes. Alkynes were identified by C≡C stretching (2100–2145 cm^−1^), and aromatic compounds were detected by C-H bending overtones (1650–2000 cm^−1^). Specific aromatic substitution patterns were detected, including monosubstituted (750 ± 20 cm^−1^), para-disubstituted (810 ± 20 cm^−1^), and trisubstituted (880 ± 20 cm^−1^) rings. Additional signals unique to certain medium Mannitol extracts included C-O stretches (aromatic esters), O=C=O stretches (carbon dioxide), O-H bending (phenols) and S-H stretching (thiols) (Supplementary Table S1).

In contrast, extracts from medium 5294 (Supplementary Table S2) displayed N-H stretching (3200–3400 cm^−1^) and bending (1580–1650 cm^−1^) vibrations characteristic of primary amines. Halo compounds were indicated by C-Br or C-I stretching (500–690 cm^−1^). Primary amides (± 1650 cm^−1^) were uniquely identified in *B. thuringiensis* AB2-SP.

Several functional groups were common to extracts from both media (Supplementary Tables S1 and S2). Alkanes were indicated by C-H stretching of methyl (CH_3_) and methylene (CH_2_) groups (2840–3000 cm^−1^), CH_2_-CH_3_ asymmetric deformations (1360–1380 cm^−1^), and C-H bending (1375–1460 cm^−1^). Carbonyl (C=O) stretching vibrations, typical of ketones or esters, appeared between 1680 and 1725 cm^−1^. Absorption peaks between 1000 and 1250 cm^−1^ corresponded to C-O stretching of ethers; however, in medium 5294 extracts, this region could also reflect C-N stretching of amines.

#### 2.4.2. Gas Chromatography-Mass Spectrometry Characterization

Gas chromatography-mass spectrometry (GC-MS) was conducted on extracts from five *Bacillus* species cultured in two distinct fermentation media, i.e., medium Mannitol (Supplementary Table S3) and medium 5294 (Supplementary Table S4), to identify and compare secondary metabolite profiles. Extracts from medium Mannitol yielded a greater diversity of metabolites, with 300 compounds identified, compared to 195 in medium 5294.

With Mannitol extracts (Supplementary Table S3), 79.33% (238/300) of the compounds were unique to specific *Bacillus* isolates: *B. thuringiensis* AB2-SP (*n* = 49), *B. cereus* SP1-AB4 (*n* = 26), *B. mobilis* SP2-AB7 (*n* = 48); *B. pumilus* SP2-W6 (*n* = 67), and *B. wiedmannii* SP5-AB7 (*n* = 48). Similarly, in medium 5294 (Supplementary Table S4), 70.26% (137/195) of the detected compounds were isolate-specific: AB2-SP (*n* = 52), SP1-AB4 (*n* = 44), SP2-AB7 (*n* = 4); SP2-W6 (*n* = 5), and SP5-AB7 (*n* = 32). These results underscore the strong isolate-specific nature of secondary metabolite production, even under similar fermentation conditions within the same genus.

Some compounds were consistently present across all isolates within each medium. In medium Mannitol, the alkanes eicosane and heptadecane were common to all five isolates. In contrast, medium 5294 extracts, shared only a single compound across all isolates, i.e., 1,2-benzenedicarboxylic acid, bis(2-methylpropyl) ester (phthalate ester). However, this compound was also detected at high abundance in the uninoculated medium 5294 control (66.45% area), suggesting it originates from the medium rather than microbial metabolism.

Further, an inverse correlation was observed between the abundance of this phthalate ester and coverall metabolite diversity. Isolates such as *B. thuringiensis* AB2-SP (*n* = 96), *B. cereus* SP1-AB4 (*n* = 89), and *B. wiedmannii* SP5-AB7 (*n* = 42) exhibited greater compound diversity and lower levels of the ester, whereas *B. mobilis* SP2-AB7 (*n* = 11) and *B. pumilus* SP2-W6 (*n* = 12) showed lower diversity and higher ester abundance (Supplementary Table S4). This suggests that elevated levels of 1,2-benzenedicarboxylic acid, bis(2-methylpropyl ester) in medium 5294 may suppress metabolite production in certain isolates.

#### 2.4.3. Isolate-Specific Metabolite Profiles

For *B. thuringiensis* AB2-SP, 61.25% (49/80) of the compounds in medium Mannitol were unique, with four major compounds identified (Supplementary Table S3). In medium 5294, 54.67% (52/96) were unique, with eight major compounds identified (Supplementary Table S4). Benzeneacetic acid, ethyl ester was exclusively to medium Mannitol, while phthalic acid, di(2-propylpentyl) ester was unique to medium 5294.

For *B. cereus* SP1-AB4, 56.52% (26/46) of compounds in medium Mannitol were unique, with eight major compounds identified (Supplementary Table S3). In medium 5294, 49.43% (44/89) were unique, with six major compounds identified (Supplementary Table S4). Phthalic acid, dodecyl octyl ester was exclusive to medium Mannitol, while phthalic acid, di(6-methylhept-2-yl) ester appeared in both media.

For *B. mobilis* SP2-AB7, 70.59% (48/68) of compounds in medium Mannitol were unique, with four major metabolites identified (Supplementary Table S3). In medium 5294, 36.36% (4/11) of compounds were unique, with seven major compounds identified (Supplementary Table S4). Malic acid was exclusive to medium Mannitol, while 3-hexanol, 2,4-dimethyl was unique to medium 5294.

The *B. pumilus* SP2-W6, 76.14% (67/88) of compounds in medium Mannitol were unique, with nine major compounds (Supplementary Table S3). In medium 5294, 41.67% (5/12) of compounds were unique, with four major compounds identified (Supplementary Table S4).

For *B. wiedmannii* SP5-AB7, 61.54% (48/78) of compounds in medium Mannitol were unique, with eight major metabolites (Supplementary Table S3). In medium 5294, 76.19% (32/42) of compounds were unique, with six major compounds (Supplementary Table S4). Phthalic acid, di(2-ethylcyclohexyl) ester and spiro[androst-5-ene-17,1′-cyclobutan]-2′-one, 3-hydroxy-, (3β,17β)- were exclusive to medium Mannitol and medium 5294, respectively.

### 2.5. Sponge-Associated Bacterial Extracts Display QSI Activity Against C. violaceum ATCC 12472

All 10 bacterial extracts (five from medium Mannitol and five from medium 5294) were screened for long-chain QSI using *C. violaceum* ATCC 12472 in an agar overlay-well diffusion assay, with furanone serving as the positive control.

Among the Mannitol-derived extracts, 80% (4/5) exhibited QSI activity against the long-chain AHL-producing *C. violaceum* biosensor (Supplementary Table S5). In contrast, only two extracts from medium 5294 demonstrated violacein inhibition, indicating a reduced frequency of QSI activity in this medium (Supplementary Table S5).

### 2.6. Antimicrobial Testing of Sponge-Associated Bacillus Species Extracts Against Pseudomonas Aeruginosa ATCC 27853

To confirm that the observed QSI and anti-virulence effects were not attributable to bactericidal activity, all 10 extracts were evaluated for antimicrobial activity using agar well diffusion assays at concentrations of 5 and 10 mg. No zones of inhibition were observed at either concentration, indicating that none of the extracts exhibited direct antimicrobial effects against *P. aeruginosa* ATCC 27853.

### 2.7. Assessing P. aeruginosa ATCC 27853 Virulence Factor Inhibition

#### 2.7.1. *P. aeruginosa* Pyocyanin Production Inhibition by *Bacillus* Species Extracts

All 10 *Bacillus* extracts inhibited pyocyanin production by *P. aeruginosa* to varying degrees, ranging from −0.73% to 39.36%, with associated cell death remaining below 40% (Supplementary Figures S3.1–S3.6). Of these, 80% (8/10) reduced pyocyanin production by >30% (Table 3).

At the isolate level, *B. wiedmannii* SP5-AB7 (Mann) exhibited the highest inhibition, with a maximum reduction of 39.36% at 750 µg/mL (Supplementary Figure S3.6A; Table 3). This extract consistently showed the strongest inhibitory activity across all tested concentrations, except at 1000 µg/mL, where inhibition decreased to 32.69%. While none of the extracts achieved ≥50% inhibition, all significantly reduced pyocyanin production (*p* < 0.01), with the highest inhibition observed at 1000 µg/mL. Notably, extracts from medium Mannitol appeared to be more effective overall in suppressing pyocyanin production.

#### 2.7.2. *P. aeruginosa* Pyoverdine Production Inhibition by *Bacillus* Species Extracts

Pyoverdine inhibition ranged from 2.51 to 38.43%, with 50% (5/10) of the extracts reducing pyoverdine production > 30% (Supplementary Figures S3.1–S3.6). The highest average inhibition was observed at 1000 µg/mL, reaching 24.05% (*p* < 0.01; Table 3). Importantly, no significant cytotoxicity was detected at any concentration (Supplementary Figures S3.1–S3.6).

At the isolate level, the extract from *B. thuringiensis* SP-AB2 (Mann) exhibited the greatest inhibition, with a 38.43% reduction at 1000 µg/mL (Table 3; Supplementary Figure S3.2A). Interestingly, the positive control cinnamaldehyde unexpectedly increased pyoverdine production (Supplementary Figure S3.1A). Overall, *Bacillus* species extracts significantly inhibited pyoverdine production manner (*p* < 0.01) in a dose-dependent manner (Table 3).

#### 2.7.3. *P. aeruginosa* Elastase Production Inhibition by *Bacillus* Species Extracts

Elastase (LasB) inhibition ranged from −8.20% to 71.32%, with 60% (6/10) of extracts achieving ≥30% inhibition (Table 4; Supplementary Figures S4.1–S4.6). The strongest inhibitory effects were observed with extracts derived from medium Mannitol at 1000 µg/mL (Table 4).

Three Mannitol-derived extracts achieved >50% inhibition at 1000 µg/mL (Table 4), exceeding the performance of the cinnamaldehyde control, which showed 42.67% inhibition before exhibiting bactericidal effects (Supplementary Figure S4.1A). The most potent elastase inhibitor was the *B. wiedmannii* SP5-AB7 (Mann) extract, which reached 71.32% inhibition at 1000 µg/mL without affecting cell viability (Supplementary Figure S4.6A). Overall, elastase activity was significantly reduced by all *Bacillus* extracts in a concentration-dependent manner (Table 4; *p* < 0.01).

#### 2.7.4. *P. aeruginosa* Protease Production Inhibition by *Bacillus* Species Extracts

**Figure 2:**
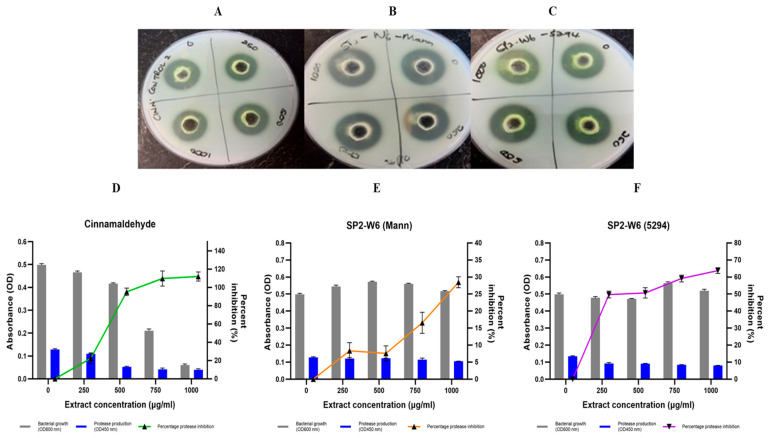
Inhibition of *Pseudomonas aeruginosa* protease production. Qualitative assessment based on skim milk agar hydrolysis comparing untreated samples with those treated using cinnamaldehyde (positive control, (**A**)), *Bacillus pumilus* SP2-W6 (Mann) extract (**B**), and SP2-W6 (5294) extract (**C**). Quantitative inhibition of growth and protease production using cinnamaldehyde (**D**), SP2-W6 (Mann) extract (**E**), and SP2-W6 (5294) extract (**F**). Data represents mean ±SD from three independent experiments, each performed in triplicate. Growth inhibition by both extracts was ≤40%, whereas cinnamaldehyde showed ≥40% inhibition from 750 µg/mL, which contributed to reduced protease production but was not indicative of quorum sensing inhibition (QSI).

Protease inhibition was assessed using both a qualitative casein agar well diffusion assay and a quantitative azocasein spectrophotometric assay. In the qualitative assay, inhibition was inferred from reduced casein hydrolysis zones. Only *B. pumilus* SP2-W6 extracts (from both Mannitol and 5294 media) showed visible reductions in hydrolysis zones (Figure 2). The cinnamaldehyde control demonstrated strong inhibition (Figure 2A), though reduced hydrolysis at 1 mg/mL was attributed to bactericidal activity. Across all 10 extracts, hydrolysis zones ranged from 10.50 to 30.39 mm (Supplementary Table S6).

In the quantitative azocasein assay, 40% (4/10) of the extracts achieved ≥50% inhibition at 1000 µg/mL (Table 4). No extract caused more than 40% cell death (Supplementary Figures S5.1–S5.6). The most effective protease inhibition was observed with extracts from medium 5294 (Table 4). The *B. pumilus* SP2-W6 (5294) extract exhibited strong, concentration-dependent inhibition, from 49.73% at 250 µg/mL to a maximum of 63.81% at 1000 µg/mL (Figure 2F).

Additional extracts with ≥45% inhibition included SP-AB2 (5294), SP1-AB4 (Mann), SP2-AB7 (5294), and SP5-AB7 (5294). DMSO (10%) alone increased protease production (Supplementary Figure S5.1B), while cinnamaldehyde significantly reduced it (*p* < 0.05; Figure 2D), likely due to its cytotoxic effects. Overall, *Bacillus* extracts significantly inhibited protease production in *P. aeruginosa* (*p* < 0.01), with a consistent dose-dependent response (Table 4).

#### 2.7.5. *P. aeruginosa* Rhamnolipid Production Inhibition by *Bacillus* Species Extracts

**Figure 3:**
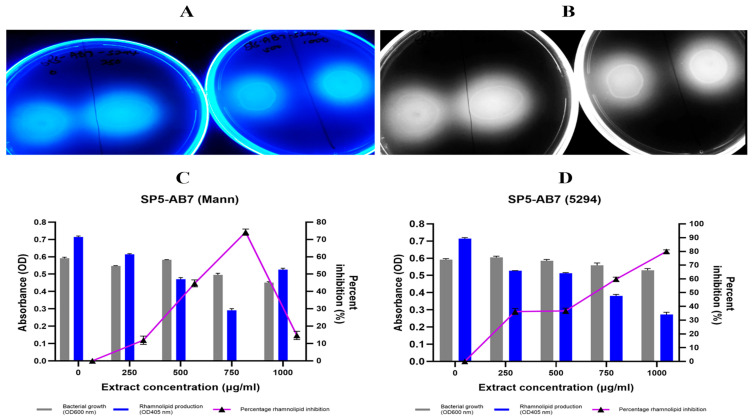
Rhamnolipid inhibition by *Bacillus wiedmannii* SP5-AB7 extracts. (**A**) CTAB agar assay showing reduced blue halos under UV light, indicating inhibition of rhamnolipid production by the SP5-AB7 (5294) extract. (**B**) The same assay under greyscale enhances visualization of inhibition zones. Spots from left to right correspond to *Pseudomonas aeruginosa* ATCC 27853 treated with 0, 250, 500, and 1000 µg/mL of SP5-AB7 (5294) extract. (**C**,**D**) Quantitative analysis of growth and rhamnolipid production inhibition by SP5-AB7 (Mann) (**C**) and SP5-AB7 (5294) (**D**) extracts. Data represent mean ± SD from three independent experiments performed in triplicate. Growth inhibition for both extracts was ≤40%.

Rhamnolipid production was assessed qualitatively using the CTAB agar assay, where blue halos form due to interactions between the cationic CTAB and anionic rhamnolipids. A reduction in halo size indicates rhamnolipid inhibition (Figure 3A). At 1000 µg/mL, inhibition ranged from 9.46% to 67.41% across all extracts (Supplementary Table S7).

Quantitative analysis using the orcinol assay revealed that 50% (5/10) of the *Bacillus* extracts inhibited rhamnolipid production by >50% (Table 5; Supplementary Figures S6.1–6.6). The most potent inhibitors were *B. wiedmannii* SP5-AB7 (Mann) (Figure 3C) and *B. mobilis* SP2-AB7 (Mann) (Supplementary Figure S6.4A), both of which exhibited significant inhibition without cytotoxic effects. Specifically, *B. wiedmannii* SP5-AB7 (Mann) reduced rhamnolipid levels by 88.44% at 1000 µg/mL and up to 93.88% at 500 µg/mL (Figure 3C), while *B. mobilis* SP2-AB7 (Mann) achieved inhibition ranging from 79.47% (500 µg/mL) to 86.03% (250 µg/mL).

Statistically significant mean inhibition was observed only at concentrations of ≥500 µg/mL (Table 5). Neither the furanone control (tested at 2.5–10 µg/mL) nor 10% DMSO (solvent control) significantly affected rhamnolipid production (*p* > 0.05).

#### 2.7.6. *P. aeruginosa* Swimming Motility Inhibition by *Bacillus* Species Extracts

**Figure 4:**
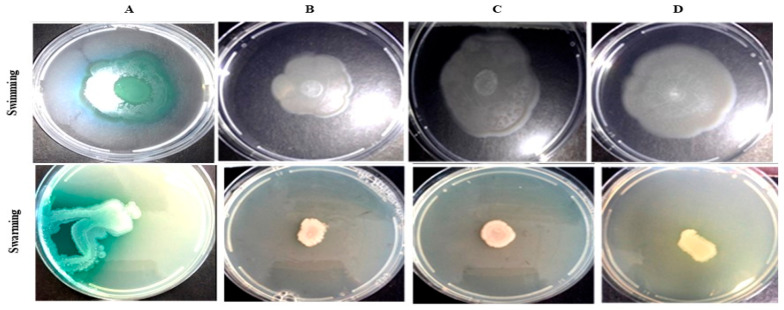
Effect of sponge-associated bacterial extracts on *Pseudomonas aeruginosa* ATCC 27853 motility. Positive inhibition was defined as a sustained reduction in swimming and swarming zones after 72 h. (**A**–**D**) Swimming motility following treatment with 0, 250, 500, and 1000 µg/mL of *Bacillus mobilis* SP2-AB7 (5294) extract. (Swimming: (**A**–**D**)) Swarming motility following treatment with 0, 250, 500, and 1000 µg/mL of *Bacillus cereus* SP1-AB4 (5294) extract (Swarming: (**A**–**D**)). In both cases, pyocyanin production was also inhibited.

The impact of *Bacillus* extracts on *P. aeruginosa* swimming motility was monitored over 72 h. Only sustained inhibition observed at 72 h were considered significant. The most notable inhibition was achieved by *B. mobilis* SP2-AB7 (5294), which reduced motility by 55.88% at 250 µg/mL (Figure 4A).

Overall, 80% (8/10) of the extracts reduced swimming motility by >30% (Supplementary Table S8), though only one extract surpassed 50% inhibition (Table 5). *Bacillus cereus* SP1-AB4 (5294) also demonstrated notable inhibition, achieving 40.29% reduction at 250 µg/mL. None of the extracts caused bacterial growth inhibition.

*Bacillus* extracts induced a statistically significant reduction in swimming motility (*p* < 0.01), with each concentration contributing to the observed effect (Supplementary Table S8). The strongest inhibition was observed at 250 µg/mL with medium 5294-derived extracts. Although cinnamaldehyde reduced swimming by >50%, this was not considered significant due to associated cytotoxicity at 1000 µg/mL (Supplementary Table S8).

#### 2.7.7. *P. aeruginosa* Swarming Motility Inhibition by *Bacillus* Species Extracts

Swarming motility was evaluated over 72 h using measurements taken every 24 h (Supplementary Table S9). Sustained reductions at 72 h were used to assess the inhibitory effect of the extracts (Figure 4B). Three extracts (30%) inhibited swarming motility by >50%. The strongest inhibition was observed with *B. cereus* SP1-AB4 (5294), which achieved a 56.96% reduction at 250 µg/mL. *Bacillus mobilis* SP2-AB7 extracts (both Mann and 5294 media) also demonstrated significant inhibition, reducing swarming by 59.49% and 55.7%, respectively, at 500 µg/mL. Overall, the extracts caused a significant reduction (*p* < 0.01) in swarming motility at 72 h, with statistically significant inhibition observed at 250 µg/mL (*p* < 0.05), and at 500 and 1000 µg/mL (*p* < 0.01) (Supplementary Table S9).

A summary of virulence factor inhibition at 1000 µg/mL for both medium Mannitol and medium 5294 extracts is provided in Table 6.

#### 2.7.8. *P. aeruginosa* Initial Adhesion Inhibition by *Bacillus* Species Extracts

The impact of the 10 *Bacillus* extracts on *P. aeruginosa* initial adhesion was evaluated at concentrations of 0, 0.5, 1, 5, and 10 mg/mL (Table 7). Inhibition ranged from −17.73% to 73.58% (Supplementary Figure S7). Overall, 40% (4/10) of the extracts reduced initial adhesion by >50%, with three achieving >65% inhibition without inducing ≥40% bacterial growth inhibition.

Although some extracts exceeded 75% inhibition, this was primarily linked to cytotoxicity at higher concentrations. Three extracts demonstrated >60% inhibition, while maintaining cell viability, suggesting possible QS-mediated interference with early biofilm development. These included: *B. cereus* SP1-AB4 (Mann) with 73.58% reduction at 10 mg/mL; *B. cereus* SP1-AB4 (5294) with 69.39% inhibition at 5 mg/mL; and *B. wiedmannii* SP5-AB7 (Mann) with 66.51% inhibition at 5 mg/mL (Table 7).

Overall, the extracts significantly reduced *P. aeruginosa* initial adhesion (*p* < 0.01), although inhibition at 0.5 mg/mL was not statistically significant. The cinnamaldehyde control demonstrated adhesion inhibition only at 5 and 10 mg/mL, but this was associated with cell death (Table 7; Supplementary Figure S7).

#### 2.7.9. *P. aeruginosa* Mature Biofilm Inhibition by *Bacillus* Species Extracts

To assess the effect on established biofilms, all 10 *Bacillus* extracts were tested against mature *P. aeruginosa* (pre-formed) biofilms at 0, 0.5, 1, 5 and 10 mg/mL (Supplementary Figure S8). Among all tested extracts, only *B. thuringiensis* SP-AB2 (Mann) achieved a ≥40% inhibition, reaching a maximum reduction of 86.28% at 10 mg/mL (*p* < 0.05; Table 7). Despite limited high-level inhibition across the full extract set, a statistically significant overall decrease in mature biofilm biomass was observed (*p* < 0.01), with each concentration contributing significantly to the effect.

## 3. Discussion

Quorum sensing is widespread in the marine environment [11], with QS signal-producing microbes, free-living or host-associated coordinate behaviours such as secondary metabolite production, biofilm formation, and virulence. Bacteria from the genera *Pseudoalteromonas*, *Thalassomonas*, *Pseudomonas*, *Roseobacter*, *Aeromonas* and *Vibrio* are common QS signal producers in this environment [42] and predominantly utilize AI-1 (AHLs and hydroxyketones) and AI-2 (furanosyl-borate diesters) QS systems. These signalling molecules regulate vital microbial behaviours such as secondary metabolite production, virulence factor expression, enzyme secretion and biofilm development. In marine microbial communities, AHL-based QS dominate, being primarily associated with ecological and biogeochemical processes, while AI-2-QS regulates interspecies interactions within complex microbial consortia [42]. Several marine macro-organisms (e.g., coral, sponges and algae) and their microbial symbionts (e.g., *Bacillus, Pseudovibrio, Streptomyces, Vibrio* species and fungi), have evolved QS inhibitory (QSI) mechanisms. Both secondary metabolite-based QSI and enzymatic QQ appear to be widespread in marine environments, highlighting the ecological and therapeutic relevance of chemical interference in microbial competition [42].

Sponge holobionts, in particular, are rich sources of symbiotic microbes with biosynthetic potential [11,14,21,42]. In this study, 98 bacterial isolates from seven intertidal sponges in KwaZulu-Natal were screened for QSI activity using *Chromobacterium violaceum* biosensor sandwich assays. A large proportion (87.76%; 86/98) inhibited short-chain Ahl-mediated signalling, while only 17.35% (17/98) inhibited long-chain signals (Figure 1), consistent with the known preference of AHL-lactonases for short-chain AHLs (<C8), and increased stability and persistence of long-chain AHLs [43]. Comparative studies have reported similar trends: Gutiérrez-Barranquero et al. [38] found that only 4.09% (18/440) of sponge-associated isolates exhibited QSI activity, while Singh et al. [40] reported a higher rate of 40% (4/10). Such variability in QSI profiles among sponge-associated bacteria reflects differences in bacterial identity, signal structure, and assay conditions.

Although *Bacillus* spp. have been widely studied for antimicrobial activity, their QSI potential against *Pseudomonas aeruginosa* remains underexplored [38,39,44,45,46]. Here, 10 extracts obtained from five *Bacillus* strains exhibited differential inhibition of QS-regulated phenotypes in *P. aeruginosa* ATCC 27853, including pigment production, enzyme secretion, motility, and biofilm formation. Notably, several extracts demonstrated broad-spectrum activity across Las, Rhl, and PQS systems, with isolate- and fermentation medium-specific variation. The inhibition of QS-regulated virulence phenotypes by these marine-derived isolates, at a concentration of 1 mg/mL, is summarized in Table 6, with the highest inhibition values across a concentration range detailed in Supplementary Table S10.

**Pyocyanin and Pyoverdine**: The phenazine pigment pyocyanin is a key factor in chronic *P. aeruginosa* infections, notably in cystic fibrosis patients’ sputum and in the urine of individuals with persistent infections. Pyocyanin contributes to host cell damage, including neutrophil apoptosis [47]. Pyocyanin inhibition was evaluated alongside pyoverdine, a siderophore involved in iron acquisition. Most extracts (80%) moderately suppressed pyocyanin and pyoverdine (30–40%) (Table 6; Supplementary Table S10). This aligns with findings from Saurav et al. [21], who observed that only 35.29% (6/17) of their sponge-derived isolates exhibited >70% pyocyanin inhibition at approximately 1 mg/mL. Similarly, Gutiérrez-Barranquero et al. [38] reported >50% inhibition by only 20% (1/5) of their sponge-associated *Bacillus* isolates. The *B. wiedmannii* SP5-AB7 (Mann) extract demonstrated the highest pyocyanin inhibition (39.36% at 750 µg/mL). This is consistent with Mani et al. [48] who reported a maximal pyocyanin inhibition of 25% using 1 mL of crude extracts from three *Bacillus* strains. Musthafa et al. [46] reported ~40% inhibition at 1000 µg/mL, closely matching the inhibition achieved by *B. wiedmannii* SP5-AB7 (Mann) with *Bacillus* species from intertidal sediment. Furthermore, they noted enhanced inhibition (up to 86%) at 2000 µg/mL, suggesting that future studies should explore a broader concentration range to capture the full potential of these extracts.

Pyoverdine is a key siderophore that functions both as an iron-chelating molecule and a signalling molecule, by obtaining iron for the bacterial cells, and up-regulates the production of exotoxin A and endoprotease [49]. An overall significant decrease in pyoverdine inhibition was observed (*p* < 0.01) with extracts. Only five of the extracts inhibited pyoverdine production by 30–40%, compared to the eight extracts inhibiting pyocyanin production. Although both virulence factors are regulated through interconnected QS pathways, iron availability and the efficacy of iron sequestration can influence the regulatory balance. Under iron-limited conditions, the *las* system may be bypassed in favour of the *rhl* system which can subsequently reactivate the PQS system [50]. Iron-limiting conditions may shift QS dynamics, possibly accounting for the lower pyoverdine suppression observed, as disruptions in iron sequestration, such as inhibition of siderophore production, can lead to feedback activation of other QS pathways. The *B. thuringiensis* SP-AB2 (Mann) extract was the most potent of the five extracts with activity, demonstrating a concentration-dependent inhibition of pyoverdine production, with a maximum reduction of 38.43% at 1000 µg/mL and 32.63% inhibition of pyocyanin. Musthafa et al. [46] reported a dose-dependent decrease in pyoverdine production by *P. aeruginosa* treated with marine *Bacillus* species extracts (50–2000 µg/mL), although the extent of inhibition was not quantified. Pyoverdine is often overlooked in QSI studies, likely due to its modest standalone effect on virulence ([51]. Nonetheless, its inclusion provides valuable insight into the broader regulatory impact of QS inhibition and the potential of targeting iron acquisition pathways in anti-virulence strategies.

**Elastase and Protease**: Protease production is another key virulence factor facilitating host tissue invasion by *P. aeruginosa* and is predominantly regulated by the long-chain *las* QS system. The *las* QS system in *P. aeruginosa* is the primary regulator of elastase (LasB) production [48,52]. Three *Bacillus* species extracts derived from medium Mannitol demonstrated >50% elastase inhibition at 1000 µg/mL, highlighting mannitol’s suitability as an optimized production substrate for generating metabolites that target elastase expression. *Bacillus wiedmannii* SP5-AB7 (Mann) achieved 71.32% elastase inhibition at 1000 µg/mL, surpassing previously reported *Bacillus* extract effects. Musthafa et al. [46] observed a maximal elastase production decrease of 68% at 2000 µg/mL with a marine *Bacillus* species extract, while Mani et al. [48] obtained elastase inhibition of 37% following treatment with 1 mL of their crude *Bacillus* species extracts. The substantial inhibition of elastase by these mannitol-derived extracts suggests their potential as effective anti-virulence agents targeting the *las* QS system in *P. aeruginosa*, with further optimization of culture conditions possibly enhancing their therapeutic efficacy.

Four of the sponge-associated *Bacillus* spp. extracts demonstrated >50% inhibition of protease activity at 1000 µg/mL, consistent with Musthafa et al. [46], who reported protease inhibition levels of 37% and 65% at 1000 and 2000 µg/mL, respectively, using crude marine *Bacillus* species extracts. Mani et al. [48], however, observed more modest inhibition, ranging from 17 to 33% across three *Bacillus* spp. extracts. Among the tested isolates, *Bacillus pumilus* extract SP2-W6 (5294) exhibited strong protease inhibition (63.81% reduction at 1000 µg/mL), highlighting the influence of culture conditions on metabolite production and the potential of specific sponge-derived *Bacillus* strains as effective QS inhibitors targeting protease-mediated virulence in *P. aeruginosa*.

**Rhamnolipid:** Rhamnolipids, regulated by the Rhl system, are critical for swarming motility and early biofilm formation [53]. Six extracts inhibited rhamnolipid synthesis by over 50%. This level of inhibition exceeds the highest rhamnolipid suppression reported by Mani et al. [48], who observed comparable inhibition with their three *Bacillus* species extracts. Extracts obtained using Mannitol-enriched media proved to be the most effective, with *B. wiedmannii* SP5-AB7 (Mann) and *B. mobilis* SP2-AB7 (Mann) demonstrating 93.88% inhibition at 500 µg/mL and 86.03% inhibition at 250 µg/mL, respectively.

**Motility:** The motility of *P. aeruginosa* cells is essential for both host invasion and biofilm formation. While swimming and swarming motilities are linked to surface colonization, their regulation has yet to be conclusively attributed to a specific QS system. Instead, they appear to be influenced by a combination of external factors such as pili formation and rhamnolipid production [53,54]. The inherent variability of swarming assays, which are highly sensitive to environmental factors such as surface moisture and ambient humidity, pose challenges for reproducibility [55]. As a result, significant inhibition of swarming motility was consistently observed only at 1000 µg/mL for medium Mannitol-derived extracts and at ≥500 µg/mL for those from medium 5294.

In contrast to the findings by Gutiérrez-Barranquero et al. [38], who reported no swimming inhibition by *Bacillus* spp. extracts, 70% of the extracts in this study exhibited >30% inhibition of swimming motility, with *B. mobilis* SP2-AB7 (5294) achieving >50% inhibition. For swarming motility, 70% (7/10) of extracts inhibited swarming by >30%, and three demonstrated inhibition between 50 and 60%, results consistent with Gutiérrez-Barranquero et al. [38], who observed >50% inhibition in 60% of their extracts. Notably, *B. mobilis* SP2-AB7 (Mann) reduced swarming diameter from 39.5 mm (control) to 16 mm (59.5%), comparable to the 81.25% reduction observed by Rekha et al. [56] using *Cassia alata* extract. However, unlike Rekha et al. [56] who did not assess the duration of inhibition, *B. mobilis* SP2-AB7 (Mann) maintained significant motility suppression over 72 h. Notably, *B. mobilis* SP2-AB7 (5294) reduced swimming and swarming motility types by >50%, suggesting a multi-target mechanism.

**Biofilm:** Biofilm formation in *P. aeruginosa* is a multifaceted process regulated by various factors, including virulence determinant production, cellular motility, EPS synthesis, as well as QS-regulated small molecules and sRNAs [57,58]. Initial adhesion was reduced by ≥40% in half of the extracts. Three extracts demonstrated >65% inhibition with *B. cereus* SP1-AB4 (Mann) achieving the highest inhibition (74%). Gutiérrez-Barranquero et al. [38] observed that only one of their five *Bacillus* spp. extracts inhibited initial adhesion by ≥50%, while two extracts had <50% inhibition. Musthafa et al. [46] documented strong inhibition of *P. aeruginosa* initial adhesion (88% at 2000 µg/mL) using a marine *Bacillus* species extract. Similarly, Sayem et al. [59] achieved a 90% inhibition of initial adhesion of *Pseudomonas fluorescens* using an EPS preparation from sponge-associated *B. licheniformis*, underscoring the QSI potential of marine-derived *Bacillus* species. The relatively modest inhibition of initial adhesion observed in the present study may be partially attributed to the high rhamnolipid inhibition by several extracts. Previous studies have shown that early rhamnolipid suppression, prior to biofilm maturation, can paradoxically enhance initial biofilm formation [57], as *rhl*-deficient mutants often display increased surface adherence [46]. Only one extract, *B. thuringiensis* SP-AB2 (Mann), strongly inhibited mature biofilm production (86.28%). Gutiérrez-Barranquero et al. [38] similarly observed that only a single *Bacillus* species extract inhibited pre-formed, mature biofilm by >50%. These results underscore the difficulty in disrupting established biofilms and suggest differential metabolite action on early vs. mature biofilm stages, attributable in part to the complex EPS in mature biofilms [57].

**QS Pathway Stratification:** To clarify the mode of action of the extracts, phenotypic effects were mapped to their corresponding QS systems [60,61]. Given the semi-independent regulation of virulence traits across Las, Rhl, and PQS networks, the top three inhibitory extracts from each assay were selected for comparative analysis, with the aim of identifying trends related to bacterial isolate origin and fermentation medium (Table 6; Supplementary Table S10). This stratified approach revealed that sponge-associated *Bacillus* species exhibit diverse and broad-spectrum QSI activity, with some extracts targeting multiple QS-regulated phenotypes simultaneously. It also highlighted the strong influence of culture conditions on metabolite efficacy.

**Las System:** Considerable variability was observed in elastase and protease inhibition across extracts. Mannitol-derived extracts from isolates SP1-AB4, SP2-AB7 and SP5-AB7 were particularly effective against elastase, while 5294-derived extracts from SP1-AB2, SP2-AB7 and SP2-W6 exhibited strong protease inhibition. Among these, the *B. cereus* SP1-AB4 (Mann) extract stood out, demonstrating >50% inhibition of initial adhesion, elastase, and protease production. The enhanced performance of Mannitol-based extracts may be due to its role as a sugar alcohol elicitor, which can stimulate the production of QS-interfering secondary metabolites. In contrast, medium 5294, although rich in starch and glucose and conducive to higher biomass and general metabolite production, may not specifically induce QS-inhibitory compound synthesis.

**Rhl and PQS Systems:** Strong inhibitory activity was observed for traits associated with these systems. Rhamnolipid production was suppressed by >75% in several extracts while pyocyanin and pyoverdine production showed moderate inhibition (30–40%). *B. wiedmannii* SP5-AB7 (from both media) effectively suppressed rhamnolipid and pyocyanin production, suggesting robust interference with short-chain QS signalling. However, these extracts had limited impact on mature biofilm formation, indicating a likely restriction to early-stage QS phenotypes.

In contrast, *B. thuringiensis* SP-AB2 (5294) achieved the highest inhibition of mature biofilm formation (86.28%) but was less effective against rhamnolipid production, and only moderately inhibited pyocyanin (32.63%) and pyoverdine (38.48%). Similarly, *B. mobilis* SP2-AB7 (Mann) strongly inhibited rhamnolipid synthesis but was relatively ineffective against mature biofilms. In contrast, *B. thuringiensis* SP-AB2 (5294) achieved the highest inhibition of mature biofilm formation (86.28%) but was less effective against rhamnolipid production, and only moderately inhibited pyocyanin (32.63%) and pyoverdine (38.48%). Similarly, *B. mobilis* SP2-AB7 (Mann) strongly inhibited rhamnolipid synthesis but was relatively ineffective against mature biofilms. These patterns reflect selective interference with different stages of the QS cascade and underscore the complex crosstalk between Rhl and PQS systems.

**Motility and Initial Adhesion:** The regulation of swimming and swarming motility, along with initial surface adhesion, remains incompletely defined within the QS network and appears to be highly context dependent. *Bacillus mobilis* SP2-AB7 (5294) inhibited both swimming and swarming motility by over 50%, whereas its Mannitol-derived counterpart suppressed only swarming (59.49%) and had no significant effect on swimming. Neither extract was especially effective against biofilm formation. By contrast, the *B. cereus* SP1-AB4 (5294) significantly inhibited swarming (56.96%) and initial adhesion (69.39%), while the Mannitol-derived extract achieved the highest initial adhesion inhibition across all extracts (73.58%). These results indicate that medium composition can influence the biosynthesis of metabolites targeting early adhesion and motility.

**Cross-System Inhibition:** While individual QS systems regulate distinct virulence traits, their regulatory overlap complicates definitive attribution of inhibition to a single pathway. Among the extracts, *B. mobilis* SP2-AB7 (5294) demonstrated the most consistent and potent anti-virulence activity across systems (Table 6; Supplementary Table S10). Other strong performers included *B. wiedmannii* SP5-AB7 (Mann), *B. mobilis* SP2-AB7 (Mann), and *B. cereus* SP1-AB4 (from both media). These findings emphasize that isolate-specific traits and fermentation conditions critically shape QSI potential.

Although the inhibition levels observed in this study were moderate (~30–40% reduction in virulence factors), such partial attenuation can nonetheless have therapeutic relevance. Even incomplete suppression of QS–regulated traits may weaken pathogen virulence, limit host tissue damage, and enhance susceptibility to host defences or antibiotic treatment. This aligns with the anti-virulence or QQ therapeutic concept, which is not necessarily aimed at eradicating pathogens but at disarming them, thereby reducing selective pressure for resistance. Comparable levels of inhibition have also been reported in other crude extract-based studies (e.g., Musthafa et al. [46] and Mani et al. [48]), suggesting that these findings are consistent with the field. While such inhibition may not suffice as a stand-alone strategy, it could be clinically meaningful in combination therapies or when targeting early-stage virulence pathways. Future in vivo investigations will be essential to validate the translational significance of these effects.

**Mechanisms of Action and Chemical Profiles:** *Bacillus* species inhibit QS through both enzymatic and non-enzymatic mechanisms [62]. Previous studies have identified AHL-acylases and AiiA lactonases from marine *B. pumilus* and other strains [37,46]. In this study, the detection of *aiiA* genes in *B. thuringiensis* SP-AB2, *B. cereus* SP1-AB4, and *B. mobilis* SP2-AB7 suggests the potential for lactonase-mediated QSI. However, gene detection alone cannot confirm enzymatic activity. Conclusive evidence would require purified enzyme assays, kinetic studies, or direct quantification of AHL degradation.

Our data suggest that most extracts likely acted via non-enzymatic small molecules, similar to the OA10 isolate described by Singh et al. [40], which interfered with signal biosynthesis or reception rather than degradation. Although extracts were prepared at 40 °C, partial heat inactivation of enzymes remains possible. Prior reports have also shown that undefined small molecules from marine *Bacillus* species can inhibit QS [38,46], including cyclo-L-proline-L-tyrosine from a marine sediment-derived *B. cereus* strain D28 [63]. Other known QSI compounds such as furanones, coumarins, and subtilosin exhibit efficacy but are often limited by cytotoxicity [64]. In contrast, the extracts tested here were non-cytotoxic, reinforcing their therapeutic potential.

As the primary aim of this study was to identify and characterize sponge-associated *Bacillus* isolates with QSI activity, these findings represent an important first step. Future work incorporating targeted enzymatic assays will be essential to validate lactonase activity, establish causal links between *aiiA* and observed QSI effects, and determine whether enzymatic quorum quenching and small-molecule inhibition can act synergistically to enhance therapeutic efficacy.

GC-MS profiling revealed media-dependent metabolite diversity among the most active extracts. The *B. mobilis* SP2-AB7 (5294) extract was dominated by 1,2-benzenedicarboxylic acid, bis(2-methylpropyl) ester (54.02%) and n-hexadecanoic acid (5.95%), both known to bind to LasR and PqsR in *P. aeruginosa* and CviR in C. *violaceum* [65,66]. Despite containing only 11 identified compounds, this extract was the most potent, suggesting that high abundance and synergism of key QS-active molecules drive efficacy. The *B. mobilis* SP2-AB7 (Mann) extract contained high levels of malic acid (20.25%) and pyrrolo [1,2-a]pyrazine-1,4-dione, hexahydro-3-(2-methylpropyl)-, a cyclic dipeptide (cyclic dipeptide: cyclo-Leu-Pro). Malic acid has known QSI and anti-biofilm activity, especially when combined with other weak acids [67,68,69], while diketopiperazines like cyclo-(Leu-Pro) inhibit QS and biofilm formation across various bacterial species [70,71,72]. Similarly, *B. wiedmannii* SP5-AB7 (Mann) featured benzeneacetic acid (13.69%) and cyclo-(Leu-Pro), aligning with its strong inhibition of rhamnolipid and pyocyanin production. Benzeneacetic acid (phenylacetic acid) has been shown to inhibit QS-dependent virulence phenotypes in *P. aeruginosa* PAO1 [70].

*B. cereus* SP1-AB4 (Mann) was rich in eicosane (18.27%) and heinecosane (5.11%), alkanes with reported anti-biofilm activity [73,74,75], possibly explaining its high inhibition of initial adhesion inhibition (73.58% BFR; Supplementary Table S10). Its 5294-derived counterpart contained 1,2-benzenedicarboxylic acid and pentadecanoic acid, both linked to QS interference and membrane destabilization [65,76].

These findings demonstrate that *Bacillus* extracts from sponge symbionts possess broad QS inhibitory activity across the Las, Rhl, and PQS systems. Specific compounds such as 1,2-benzenedicarboxylic acid, cyclic dipeptides, and phenylacetic acid, likely act via non-enzymatic routes, supporting their development as stable, non-cytotoxic anti-virulence agents (Supplementary Table S11).

Moreover, several dominant compounds identified here have not been previously reported for bioactivity, offering exciting leads for future study (Supplementary Tables S3 and S4). The variability in extract efficacy likely reflects synergistic interactions among multiple metabolites rather than a single dominant agent. This opens avenues for enhancing bioactivity through extract blending (e.g., combining *B. mobilis* SP2-AB7 (5294) with *B. weidmannii* SP5-AB7 (Mann), co-culturing, or co-administration with conventional antimicrobials combat multidrug resistant *P. aeruginosa*.

An important limitation of this study is the difficulty in fully distinguishing between QSI and bactericidal effects. While our data indicate reduced QS–regulated phenotypes, the observed growth inhibition obtained with the cinnamaldehyde control complicated interpretation. To minimize this confounding factor, assays were performed at sub-inhibitory concentrations where possible, and detailed growth inhibition data are provided in Supplementary Figures S3–S8. More definitive mechanistic studies, including viability assays, mutant strain approaches, and direct quantification of signal molecule degradation, will be necessary to conclusively disentangle bactericidal activity from QSI. Given the semi-quantitative screening methods utilized in this study, future work will subject selected extracts to more rigorous quantitative analyses (e.g., HPLC or LC-MS–based assays) to strengthen the reliability and mechanistic interpretation of the findings. Future work should focus on elucidating mechanisms of action, whether by signal synthesis interference, receptor antagonism, or competitive binding. Integration of LC-MS/MS-based metabolomics, bioassay-guided fractionation, and molecular docking will be essential for validating these extracts as therapeutic leads.

## 4. Materials and Methods

### 4.1. Isolation of Sponge-Associated Bacteria

Seven intertidal marine sponges were collected along the KwaZulu-Natal coastline in South Africa (29°59′58″ S and 30°56′51″ E; Supplementary Figure S1). Sponge samples were collected in ziplock bags containing sea water, transported to the laboratory on ice and processed within 3 h of collection. Sponge tissues were rinsed with sterile distilled water and 1 g sample of each sponge tissue was homogenized in 9 mL of sterile sea water. The sponge homogenate was serially diluted and plated onto three selective media: Actinomycetes isolation agar (AIA, HiMedia, Thane, India), Glycerol-asparagine agar (GAA, HiMedia); Seawater yeast extract (SWYE) [77], and two non-selective media (Luria–Bertani agar and Enriched Anacker and Ordal’s agar) [78]. All media were made up using filtered, autoclaved seawater. Three antibiotics were incorporated into AIA, GAA and SWYE media: nystatin (0.05 mg/mL) and cycloheximide (0.02 mg/mL) to inhibit fungal growth, while nalidixic acid (0.02 mg/mL) was added to inhibit fast-growing bacteria [79].

Plates were incubated at room temperature for up to two months. Plates were examined daily and colonies picked weekly. Colonies representing different morphologies (colour, texture, shape and size) from diverse media plates were re-streaked to purity. Ninety-eight pure cultures were plated out on ISP2 agar [80], resuspended in 1 mL sterile distilled water and stored at −80 °C with 20% glycerol.

### 4.2. Quorum Sensing Inhibition Screening Using Chromobacterium violaceum

All 98 sponge-associated isolates were screened for QSI activity using a biosensor sandwich assay [81] with *Chromobacterium violaceum* strains CV026 (short-chain AHL biosensor) and VIR07 (long-chain AHL biosensor) [82,83]. Corresponding AHL over-producers used for the assay were *C. violaceum* ATCC 31532 [82] and ATCC 12472 [83], respectively.

Biosensor *C. violaceum* CV026 was streaked in a line on Luria–Bertani (LB) agar plates while the over-producer *C. violaceum* strain ATCC 31532 was then streaked in parallel, 15 mm apart. Test isolates were inoculated between the biosensor and the AHL donor strain, in parallel, at 7.5 mm. This allowed for the detection of QSI of short-chain AHLs (4–8 carbons in length). An identical assay was carried out with *C. violaceum* VIR07 and over-producer *C. violaceum* strain ATCC 12472 to test for the quenching of long-chain AHLs [83]. Plates were incubated at 28 °C for 24 h. QSI activity was assessed visually by the inhibition of violacein pigment production. *Bacillus cereus* ATCC 14579 was used as a QSI-positive control [81]. Results were rated on a scale of 0–4, where 0 indicated no inhibition and 4 indicated complete loss of pigment, equivalent to or greater than the *B. cereus* ATCC 14579 positive control.

### 4.3. 16S rRNA Gene-Based Identification of Bacterial Isolates

Genomic DNA isolation of 15 selected QS inhibitory bacterial isolates (Table 1) was carried out using the GeneJet Genomic DNA purification kit (Thermo Scientific, Waltham, MA, USA) for non-sporulating isolates and the ZR Fungal/Bacterial DNA MiniPrep DNA isolation kit (Zymo Research, Irvine, CA, USA) for sporulating isolates, according to manufacturers’ instructions. DNA was eluted in 100 μL of DNA elution buffer and stored at −20 °C until required.

16S rRNA gene amplification was performed for all 15 selected isolates using the following primer sets: F1: 5′-AGTTGATCCTGGCTCAG-3′ and R5: 5′-TACCTTGTTACGACTTCACCCA-3′ [84] and F1: 5′-AGAGTTTGATCITGGCTCAG-3′ and R5: 5′-ACGGITACCTTGTTACGACTT-3′ [85]. Amplification was then performed in a MJ MINI^TM^ Personal Thermal cycler (Bio-Rad, Hercules, CA, USA). PCR conditions included an initial denaturation at 95 °C for 5 min, followed by 35 cycles of 95 °C (30 s), 52 °C (1 min), and 72 °C (2 min), with a final extension at 72 °C for 5 min. Amplified products (1.5 kb) were then subjected to electrophoresis and visualized, using UV trans-illumination (Syngene, Cambridge, UK). Successfully amplified DNA was sequenced, sequences concatenated using DNAMAN Version 7 and subjected to identification using the NCBI-BLAST nucleotide database (core_nt and refseq_ma). Resulting sequences were deposited in the GenBank database (Table 1).

### 4.4. PCR Amplification of aiiA Lactonase Gene

Five isolates identified as *Bacillus* species were screened for the presence of the *aiiA* lactonase gene. Two primer sets previously described by Huma et al. [86] were used: (i) forward primer aiiAF-1: 5′-ACG TGG ATC CCG CAG GAT CCA TAT GAC AGT AAA GAA GCT T-3′ and reverse primer aiiAR-1: 5′-GCT GGT CGA CCG TCG ACT ATA TAT ATT CAG GGA A-3′, and (ii) forward primer aiiAF-2: 5′-CGG AAT TCA TGA CAG TAA AGA AGC TTT A-3′ and reverse primer aiiAR-2: 5′-CGC TCG AGT ATA TAT TCA GGG AAC ACTT-3′, with an anticipated amplimer size of 756 bp. A degenerate primer set previously described by Pan et al. [87]: AiiA1: 5′-ATGACAGTAAARAARCTTTATTTC-3′and AiiA2: 5′-TCACTATATATAYTCMGGGAACTC-3′ was also used for amplification of the *aiiA* gene. *Bacillus cereus* ATCC 14759 known to carry the *aiiA* lactonase gene was used as the positive control. Resulting amplicons were sequenced and aligned with known *Bacillus aiiA* sequences using the NCBI-BLAST nucleotide database and 753 bp DNA sequences were deposited in the GenBank database.

### 4.5. Chemical Characterization of Ethyl Acetate Culture Extracts

#### 4.5.1. Extract Preparation

Five *Bacillus* species isolates were selected for metabolite extraction. Each isolate was cultured in 5 mL of ISP2 broth for 2 d at 30 °C with shaking at 200 rpm. Thereafter, cultures were inoculated into 250 mL of medium Mannitol (20 g/L mannitol, 10 g/L yeast extract, and 1 g/L CaCO_3_) and medium 5924 (10 g/L soluble starch, 2 g/L yeast extract, 10 g/L glucose, 10 g/L glycerol, 2.5 g/L corn steep liquor, 2 g/L peptone, 1 g/L NaCl, 3 g/L CaCO_3_) broth [80]. Shake-flask fermentations were conducted in 500 mL Erlenmeyer flasks with shaking at 200 rpm at 30 °C for 7 d. Fermented broths were centrifuged at 7000 rpm for 15 min, to pellet cells, and supernatants were collected. Ethyl acetate extraction was performed according to Baltes et al. [88], with modifications. An equal volume (1:1) of ethyl acetate was added to each supernatant and then agitated at 30 °C for 1 h. The organic ethyl acetate layer was collected, and the remaining aqueous layer subjected to a second extraction of 1:1 volume ethyl acetate, with agitation at 30 °C overnight. The ethyl acetate layer was again collected, and the combined organic layers completely evaporated in a rotary evaporator (ILMVAC RObath, FisherScientific, Leicestershire, UK) at 40 °C. The dry extract was then resuspended in 4 mL methanol and added to a pre-weighed vial, after which the methanol was allowed to evaporate. Dried extracts were weighed and resuspended to a final concentration of 50 mg/mL using 10% dimethyl sulfoxide (DMSO).

#### 4.5.2. Fourier Transform Infrared (FTIR) Spectroscopy

Dried crude ethyl acetate extracts (*n* = 12; five medium Mannitol, five medium 5294 and two medium controls) were mixed with purified potassium bromide (KBr). The extract-KBr mixture was analyzed using a Bruker Alpha II Infrared Spectrometer (Karlsruhe, Germany). FTIR data was collected using ATR Diamond-1 Bounce (Bruker) with 24 sampling and background scans conducted in the range of 400–4000 cm^−1^. To smooth, baseline correct, and label peaks, data processing was conducted using the Opus Spectroscopy Software (version 8.0). Functional groups of the crude bacterial extracts were identified using IR spectra tables provided by Sigma-Aldrich (St. Louis, MO, USA).

#### 4.5.3. Gas Chromatography-Mass Spectrometry (GC-MS)

Volatile constituents making up the 10 crude ethyl acetate extracts were analyzed using a gas chromatograph (Shimadzu series AOC-20i, Kyoto, Japan) combined with a mass spectrometer (Shimadzu QP2010-SE, Japan with Zebron ZB-5MSplus capillary column 0.25 µm × 30 m (length) × 0.25 µm (film thickness)). Helium was used as the carrier gas. Run conditions included: flow rate of 0.68 mL/min, with column oven and injection temperatures maintained at 50 °C and 260 °C, respectively. Crude extracts solubilised in ethyl acetate (1 µL sample injection volume) were loaded and run with a 50-split ratio at holding times of 1- and 10 min. Analysis commenced after 3 min and ended after 32 min, with the spectra set at 20–1000 m/z to prevent interference by water molecules and other volatiles. Extracts obtained from the sterile fermentation medium (medium Mannitol and medium 5294) were also analyzed to eliminate medium-associated compounds. The recorded mass spectra of the 12 crude extracts were identified using the standard mass spectra from the National Institute of Standards and Technology (NIST05.LIB) libraries data provided by the Shimadzu GC-MS system software (2023 edition).

### 4.6. Qualitative QSI Agar Well-Overlay Screening

The QSI potential of the 10 *Bacillus* species extracts was assessed using a semi-quantitative overlay assay based on McLean et al. [89], with slight modifications. The volume of *C. violaceum* culture was increased from 50 µL to 100 µL, and *C. violaceum* ATCC 12472 was used instead of CV026. Molten LB soft agar (5 mL) was inoculated with 100 μL of an overnight, wild-type, pigmented biosensor *C. violaceum* ATCC 12472 culture. The agar-culture solution was then immediately poured over LB agar plates. Wells (6 mm in diameter) were punched into the plate using a sterile steel cork-borer and loaded with 10 µL (0.5 mg) and 20 µL (1 mg) of the 50 mg/mL extract. Extracts yielding unclear results were tested further at 2 mg. The QSI-positive control, (*Z*-)-4-bromo-5-(bromoethylene)-2(5*H*)-furanone was tested at 0.01 mg, with 10 µL and 20 µL of 10% DMSO as the negative control. Following incubation at 30 °C, the inhibition of pigment production, visible as opaque white zones around the wells, was indicative of QSI whereas clear zones around the wells indicated cell death [90].

### 4.7. Antimicrobial Testing of Bacterial Extracts Using the Agar-Well Diffusion Method

Ten sponge-associated *Bacillus* species extracts were selected and tested for their antimicrobial activity against *P. aeruginosa* ATCC 12472 using the agar-well diffusion method. An inoculum of *P. aeruginosa* ATTC 27853, equivalent to a 0.5 McFarland standard, was used to prepare bacterial lawns on Mueller-Hinton agar plates. Agar wells (8 mm diameter) were loaded with 5 and 10 mg of the 10 *Bacillus* species extracts (medium Mannitol and medium 5294), and plates were incubated at 37 °C for 24 h, after which they were examined for zones of inhibition [91].

### 4.8. Screening of Extracts Against P. aeruginosa Virulence Factor Production

*Pseudomonas* broth (PB 3 mL) was inoculated with 100 µL of an overnight *P. aeruginosa* ATCC 27853 culture standardized to an OD_600 nm_ of 1. *Pseudomonas aeruginosa* was then exposed to varying concentrations of the ethyl acetate extracts (250, 500, 750, and 1000 μg/mL) at 37 °C overnight with agitation, unless otherwise stated. Broth containing *P. aeruginosa*, but no extract, was used as a negative control while cinnamaldehyde, at concentrations corresponding to those of the bacterial extracts tested, was used as the positive control. The effect of identical volumes of 10% DMSO without extract was assessed for all assays to confirm that the solvent had no effect on virulence factor production (pyocyanin, pyoverdine, elastase, protease, rhamnolipid, motility and biofilm formation). All assays were carried out in triplicate.

Growth readings were taken at 600 nm using a microtiter plate reader (Promega Glomax Multi+ Detection System, Madison, WI, USA), where inhibition seen in conjunction with a growth inhibition of ≥40% was regarded as due to bacterial killing and not QSI. Where no specific calculation is given, reduction in virulence factor production was calculated as compared to the untreated sample:

$$\frac{{OD}_{\mathrm{Untreated}\;\mathrm{sample}}-{OD}_{\mathrm{Treated}\;\mathrm{sample}}}{{OD}_{\mathrm{Untreated}\;\mathrm{sample}}}\times 100=\mathrm{Percentage}\;\mathrm{inhibition}\;.$$

#### 4.8.1. Inhibition of Pyocyanin Production

Inhibition of pyocyanin was tested according to Essar et al. [92], with some modifications. Treated (250, 500, 750 and 1000 µg/mL) and untreated cells were centrifuged at 2500 rpm for 10 min, and supernatants extracted. Chloroform (1.5 mL) was then added to 3 mL of the supernatant and vortexed for 30 s. Following centrifugation and phase separation, 1 mL of the organic, chloroform layer was mixed with 1 mL of 0.2 M HCl and vortexed for 30 s. This mixture was then centrifuged at 7800 rpm for 10 min, the upper HCl layer removed and used to measure pyocyanin concentration. Absorbance was measured using a microtiter plate reader (Promega Glomax Multi+ Detection System) at 560 nm, using 0.2 M HCl as a blank. OD_560 nm_ was multiplied by 17.072 to obtain the pyocyanin concentration in μg/mL [92]. This value was further multiplied by a factor of 1.5, to account for the remaining organic chloroform/pyocyanin layer that remained unused [21]. Concentrations of pyocyanin from the treated and untreated samples were then compared.

#### 4.8.2. Inhibition of Pyoverdine Production

Pyoverdine was measured using a protocol described previously by Huerta et al. [93] with a modification of the volumes tested. Briefly, 0.04 mL of the aqueous phase from the pyocyanin assay was mixed with 1.96 mL of 0.05 M Tris hydrochloride buffer (pH 7.4). Fluorescence was determined by excitation at 365 nm and emission at 460 nm (Promega Glomax Multi+ Detection System), with values being given in relative fluorescence units (RFU). These readings were then divided by the respective OD_600 nm_ readings to account for the effect of growth on pyoverdine production. The percent pyoverdine inhibition was determined as follows:

$$\mathrm{Pyoverdine}\;\mathrm{inhibition}=\;\frac{\left(\mathrm{Untreated}\;RFU÷{OD}_{600nm}\right)-(\mathrm{Treated}RFU÷{OD}_{600nm})}{\mathrm{Untreated}RFU÷{OD}_{600nm}}.$$

#### 4.8.3. Inhibition of LasB Production

Elastase activity of extracts was determined using the Elastin Congo-red (ECR) assay as described by George et al. [94], with minor modifications in terms of the volumes used. Treated (250, 500 and 1000 µg/mL) and untreated samples were centrifuged at 12,000 rpm for 15 min. One mL of 0.5% elastin-Congo red (Sigma, St. Louis, MO, USA) in Tris-HCl (pH 7.4) was added to 200 μL of the cell-free supernatant and incubated at 37 °C for 24 h with agitation. Samples were vortexed and centrifuged at 1200 rpm for 15 min to remove insoluble ECR. The absorbance of the supernatants from both controls and treatments were then determined at 494 nm, using a Shimadzu 1800 UV spectrophotometer, as a measure of elastase B activity. The percentage change in absorbance was then calculated from the absorbance values.

#### 4.8.4. Qualitative Inhibition of Casein Hydrolysis

The effect of extracts (250, 500 and 1000 µg/mL) on casein proteolytic activity was assessed using an agar-well diffusion assay [78]. Treated and untreated samples (600 µL) were centrifuged three times at 12 000 rpm for 15 min to remove cells and 100 μL of the supernatant loaded into 9 mm wells within TYES agar plates enriched with 1% skim milk. This was performed in duplicate and incubated for 24 h at 37 °C. Appearance of zones of clearance around the wells was taken as a positive result, whilst no zone formation after the 24 h period was classified as negative for proteolytic ability. Zone diameters were measured and the percentage inhibition of caseinolytic activity determined as follows [95]:

$$\mathrm{Percentage}\;\mathrm{inhibition}=\left(\frac{\mathrm{Zone}\;\mathrm{diameter}\left(\mathrm{untreated}\right)-\mathrm{zone}\;\mathrm{diameter}(\mathrm{treated})}{\mathrm{zone}\;\mathrm{diameter}(\mathrm{untreated})}\right)\times 100.$$

#### 4.8.5. Quantitative Inhibition of Casein Hydrolysis

Casein hydrolysis was quantified using the azocasein assay [50] with modifications. Cell-free culture supernatants of treated (250, 500, 750 and 1000 µg/mL) and untreated samples were centrifuged at 12 000 rpm for 5 min. Protease activity was then determined using azocasein (Sigma, Berlin, Germany) as a substrate. The reaction was performed in phosphate-buffer solution (pH 7.0) with 50 μL of azocasein (30 mg/mL) and 25 μL of culture supernatant to a final volume of 750 μL. Reaction tubes were incubated at 37 °C for 1 h and subsequently stopped by adding 125 μL of 20% (*w*/*v*) trichloroacetic acid. Reaction mixtures were centrifuged at 12,000 rpm for 5 min, after which absorbance was measured at 450 nm using the Glomax Multi+ Detection System microtiter plate reader (Promega, Madison, WI, USA). A blank control was prepared using only azocasein and phosphate-buffer solution, which was incubated at the same conditions as the sample tubes [96]. Proteolytic activity was defined as the difference between the absorbance at 450 nm of the treatments and the blank. The proteolytic activity of the treated and untreated samples was then compared [96].

#### 4.8.6. Qualitative Inhibition of Rhamnolipid Production

Rhamnolipid production by treated (250, 500 and 1000 µg/mL) and untreated *P. aeruginosa* was detected using M9-glutamate minimal medium [97], with 200 µL of an overnight *P. aeruginosa* culture being spotted onto the plates containing 0.2 g cetyltrimethylammonium bromide and 5 mg methylene blue. After an overnight incubation at 37 °C, the diameter of the clear zone around the bacterial colony was measured as evidence of rhamnolipid production using a UV-transilluminator [97].

#### 4.8.7. Quantitative Inhibition of Rhamnolipid Production

Rhamnolipid inhibition was quantified following the protocol of Koch et al. [98] with adjustments in volumes only. Aliquots of the final treated (250, 500, 750 and 1000 µg/mL) and untreated supernatant (1 mL) were transferred to 1-dram glass vials and extracted twice with 1 mL of diethyl ether. The pooled organic fractions were evaporated to dryness and the resulting residue reconstituted in 200 μL of de-ionized water. In a 2 mL centrifuge tube, 75 μL of this extract was diluted into 675 μL of a solution of 0.19% (*w*/*v*) orcinol in 50% (*v*/*v*) concentrated H_2_SO_4_. Tubes were vortexed thoroughly to mix and incubated in an 80 °C heating block for 30 min. After briefly cooling to room temperature, 200 μL of the resulting yellow to yellow-orange solutions were transferred to clear 96-well microtiter plate wells, and the absorbance was measured at 421 nm (Multiskan Ascent plate reader, AEC Amersham, Madibeng, South Africa). Media background absorbance (measured from a ‘‘no bacteria’’ control) was subtracted, the resulting growth values normalized by dividing by the final OD_600 nm_. (*Z*-)-4-bromo-5-(bromoethylene)-2(5*H*)-furanone and not cinnamaldehyde, was used as a positive control at concentrations corresponding to 10% of the bacterial extracts tested.

#### 4.8.8. Inhibition of Swimming and Swarming Motility

The effect of extracts on swimming motility was conducted with swimming agar medium (10 g tryptone, 5 g NaCl and 0.3% bacteriological agar) [99]. Swim plates were prepared 24 h in advance and allowed to dry overnight within a laminar flow. Five microlitres of the treated bacterial suspension (250, 500 and 1000 µg/mL) was point-inoculated at the centre of the medium and plates incubated at 30 °C for 72 h. Swim diameters were read every 24 h to determine the effect of the extracts on swimming motility over time.

Swarming motility was assessed with LB agar overlaid with 5 mL swarming soft agar (1% peptone, 0.5% NaCl, 0.5% agar) supplemented with 0.5% D-glucose [100]. Five microlitres of treated *P. aeruginosa* bacterial suspension (250, 500 and 1000 µg/mL), was point-inoculated at the centre of the swarm medium. Plates were then incubated for 24 h at 37 °C followed by incubation at 30 °C and monitoring every 24 h over a 3 d period. The alteration in swimming and swarming migration was assessed by measuring the swim and swarm zones of the treated and untreated bacterial cells.

#### 4.8.9. Inhibition of Biofilm Production

Extracts (0.5, 1, 5 and 10 mg/mL) were tested against indicator organism *P. aeruginosa* ATTC 27853 for their effect on initial attachment and mature biofilm formation [101]. Sixteen hour-old cultures were used to prepare cell suspensions which were standardized to an OD_600 nm_ of 1. For the initial adhesion studies, extracts were added at 0.5, 1, 5 and 10 mg/mL to 90 μL TSB and 10 μL of standardized cell suspension and incubated for 24 h at 37 °C with agitation. For pre-formed (mature) biofilm detachment assays, 24 h biofilms were established following addition of 90 μL TSB and 10 μL of standardized cell suspension to microtiter plate wells, which were incubated at 37 °C for 24 h. Microtiter plates were washed three times with sterile deionised water and allowed to air-dry. Following air-drying, 90 μL TSB as well as extracts at the relevant, respective concentrations were added to wells and microtiter plates incubated for 24 h with agitation at 37 °C. The negative control contained only broth, while positive controls contained cell suspensions with no extracts added.

After incubation, planktonic cells were removed by turning the plate over and shaking out the liquid. Thereafter, the plate was washed three times with 250 μL sterile dH_2_0. Cells were fixed with 200 μL of methanol for 15 min and left to air dry. Staining was performed by adding 150 μL of 2% Hucker’s crystal violet for 5 min. Wells were gently rinsed under running water and allowed to air dry. Crystal violet was resolubilized with 150 μL of 33% (*v*/*v*) glacial acetic acid and the OD_600 nm_ values determined using microtiter plate reader (Promega Glomax Multi+ Detection System). A measure of efficacy, termed percentage reduction, was calculated from the blank, control, and treated absorbance values [102]:

$$\mathrm{Percentage}\;\mathrm{reduction}=\left(\frac{\left(C-B\right)-\left(T-B\right)}{C-B}\right)\times 100,$$

where B denotes the average absorbance per well for blank wells (no biofilm, no treatment), C denotes the average absorbance per well for control wells (biofilm, no treatment), and T denotes the average absorbance per well for treated wells (biofilm and treatment). Wells containing tryptic soy broth and *P. aeruginosa* ATCC 27853 but no extract were used as a growth control; cinnamaldehyde at 0.5, 1, 5 and 10 mg/mL was used as a positive control while 10% DMSO was used at identical volumes to confirm that it had no effect on biofilm formation.

### 4.9. Statistical Analyses

All experiments were performed in triplicate with a minimum of two experimental trials. Data were expressed as mean ±standard deviation (SD). Statistical significance was determined using one-way ANOVA followed by Tukey’s post hoc test. Following this, two-tailed *t*-tests were performed for each assay, examining the effects of medium Mannitol and medium 5294 extracts at each tested concentration. For all tests, a *p*-value of <0.05 considered statistically significant. Percentage inhibition was calculated using untreated controls as reference.

## 5. Conclusions

The growing understanding of QS in the pathogenesis of *P. aeruginosa*, as a key regulator of virulence in *Pseudomonas aeruginosa* and other clinically relevant pathogens has driven interest in anti-QS strategies that disarm rather than kill, thereby reducing selective pressure for resistance development. In this context, marine-derived *Bacillus* species, particularly those associated with sponges, represent a promising and underexplored source of anti-virulence agents.

This study demonstrates the QSI potential of sponge-associated *Bacillus* species against *P. aeruginosa*, with several extracts showing broad-spectrum anti-virulence activity. Most extracts were non-cytotoxic and acted via non-enzymatic mechanisms, involving diverse small molecules. Compounds such as 1,2-benzenedicarboxylic acid, cyclo-Leu-Pro (pyrrolo [1,2-a]pyrazine derivatives), benzeneacetic acid, and various alkanes and fatty acids emerged as promising leads. Some of these have previously been implicated in QSI and anti-biofilm activity, while others, not yet reported for their bioactivity, present exciting opportunities for further investigation. Medium composition strongly influenced metabolite profiles, indicating opportunities for optimization.

It is important to note, however, that these findings are based on five sponge-derived *Bacillus* isolates and should, therefore, be considered indicative rather than representative of the genus as a whole. While valuable in highlighting the potential of sponge-associated *Bacillus*, broader screening and comparative analyses, including terrestrial strains, will be necessary to establish the generality of these observations.

Taken together, these findings support the marine microbiome, and sponge-associated *Bacillus* in particular, as valuable sources of QSI compounds. Future work should focus on bioassay-guided fractionation, compound isolation, and mechanistic validation through LC-MS/MS and molecular docking. Co-culture strategies, extract blending, and synergy with antibiotics represent promising avenues to develop effective anti-virulence therapies against multidrug-resistant pathogens like *P. aeruginosa*.

## Figures and Tables

**Table 1:** Sandwich assay results for 15 selected sponge-associated bacterial isolates, using bacterial biosensor strains *Chromobacterium violaceum* CV026 and VIR07.

Isolate Code	CV026 (Short-Chain AHLs)	VIR07 (Long-Chain AHLs)
*Bacillus cereus* ATCC 14579	4	4
SP-AB2 (*Bacillus* sp.)	4	4
SP1-AB4 (*Bacillus* sp.)	4	3
SP1-V4	4	2
SP2-AB1	3	0
SP2-AB2	1	3
SP2-AB3	4	4
SP2-AB6	4	3
SP2-AB7 (*Bacillus* sp.)	3	+4
SP2-W3	1	2
SP2-W6 (*Bacillus* sp.)	2	2
SP3-AB22	1	+4
SP4-AB2	3	4
SP4-AB6	2	4
SP5-AB7 (*Bacillus* sp.)	1	4
SP6-AB5	3	3

Results depicted using a 0–4 number system: 0 = no discernible quenching activity as observed in biosensor control (violacein production as observed between biosensor and over-producer, with no test isolate in between) and 4 = result where complete quenching of the violacein pigment was observed as compared to the known *Bacillus cereus* ATCC 14579 quorum quenching control. +4 is quenching better than the *B. cereus* control.

**Table 2:** Identification of sponge-associated *Bacillus* species isolates using the NCBI-BLAST database.

Isolate Code	Nearest Type Strain (Accession Number)	Percentage Identity *	Sub-Group	Genbank Accession Number
SP-AB2	*Bacillus thuringiensis* (NR_114581.1)	99.85%	*B. cereus*	MH013306.1
SP1-AB4	*Bacillus cereus* (NR 115714.1)	99.93%	*B. cereus*	MH013307.1
SP2-AB7	*Bacillus mobilis* (NR_157731.1)	99.79%	*B. cereus*	MH013312.1
SP2-W6	*Bacillus pumilus* (NR_043242.1)	99.05%	*B. pumilus*	MH013314.1
SP5-AB7	*Bacillus wiedmannii* (NR_152692.1)	99.81%	*B. cereus*	MH013318.1

* Percentage identity match to the corresponding isolate using the NCBI-BLAST database.

**Table 3:** Effect of sponge-associated *Bacillus* species extracts on pyocyanin and pyoverdine production by *Pseudomonas aeruginosa* ATCC 27853. Percentage inhibition of pyocyanin and pyoverdine was evaluated for extracts obtained from cultures grown in medium Mannitol (Mann) and medium 5294 (5294) at concentrations ranging from 250 to 1000 µg/mL.

Extract Code	Percentage Pyocyanin Inhibition								Percentage Pyoverdine Inhibition							
	250 µg/mL **		500 µg/mL **		750 µg/mL **		1000 µg/mL **		250 µg/mL **		500 µg/mL **		750 µg/mL **		1000 µg/mL **	
	Mann *	5294 *	Mann **	5294 *	Mann *	5294 **	Mann **	5294 **	Mann *	5294 *	Mann **	5294 *	Mann *	5294 **	Mann **	5294 **
SP-AB2	17.80	17.99	23.02	26.58	29.57	28.24	32.63	36.21	12.05	9.47	26.19	14.61	23.13	18.52	38.43	26.89
SP1-AB4	25.66	30.09	32.72	29.05	24.75	30.60	37.57	32.37	30.15	31.33	31.43	28.26	31.32	29.80	29.37	23.96
SP2-AB7	23.10	19.83	28.31	17.58	25.09	16.81	31.54	19.70	10.55	23.82	6.39	19.02	21.45	18.87	18.02	30.92
SP2-W6	−0.73 ^**#**^	3.37	16.67	3.59	15.93	5.85	8.06	36.76	7.15	2.51	4.64	4.77	9.80	5.63	27.91	7.55
SP5-AB7	30.46	18.44	35.33	21.40	39.36	23.69	32.69	30.97	8.72	31.56	2.69	34.86	3.90	33.40	8.17	29.33
Cinnamaldehyde	9.79		12.89		37.34 ^$^		79.53 ^$^		−41.39		−57.07		−205.22 ^$^		−932.37 ^$^	
10% DMSO	17.43		−6.13 ^**#**^		10.79		0.59		13.33		15.30		12.65		18.09	

* Values carry a significance of *p* < 0.05. ** Values carry a significance of *p* < 0.01. ^**#**^ Negative values indicate an increase in pyocyanin production. ^$^ Pyocyanin/pyoverdine inhibition because of ≥40% growth inhibition and, therefore, not regarded as quorum sensing inhibition.

**Table 4:** Effect of sponge-associated *Bacillus* species extracts on elastase and protease production by *Pseudomonas aeruginosa* ATCC 27853. Percentage inhibition of elastase and protease production was assessed for extracts obtained from cultures grown in medium Mannitol (Mann) and medium 5294 (5294) at concentrations ranging from 250 to 1000 µg/mL.

Extract Code	Percentage Elastase Inhibition						Percentage Protease Inhibition (Azocasein Assay)							
	250 µg/mL *		500 µg/mL **		1000 µg/mL **		250 µg/mL **		500 µg/mL **		750 µg/mL **		1000 µg/mL **	
	Mann **	5294	Mann *	5294	Mann *	5294 *	Mann	5294 *	Mann	5294 *	Mann *	5294 **	Mann *	5294 **
SP-AB2	17.85	−4.17 ^**#**^	26.04	−0.93 **^#^**	25.66	2.35	13.85	38.55	4.96	34.47	7.08	40.72	9.19	53.51
SP1-AB4	16.63	−3.5 ^**#**^	20.74	25.16	50.32	11.39	38.89	12.47	43.69	16.35	50.07	16.01	41.02	15.66
SP2-AB7	30.92	21.63	38.75	40.17	51.76	46.01	−1.88 **^#^**	23.06	31.67	39.75	30.08	42.11	42.36	59.93
SP2-W6	14.95	−8.20 ^**#**^	43.08	−1.26 **^#^**	5.09	34.41	8.33	49.73	7.59	50.69	16.56	59.17	28.47	63.81
SP5-AB7	15.18	12.43	19.40	35.46	71.32	43.86	35.05	9.30	31.28	15.06	34.36	43.22	38.77	46.90
Cinnamaldehyde	38.30		42.67		67.45 ^$^		22.38		95.23		109.72 ^$^		111.91 ^$^	
10% DMSO	7.51		7.28		12.81		3.80		6.33		10.13		12.66	

* Values carry a significance of *p* < 0.05. ** Values carry a significance of *p* < 0.01. ^**#**^ Negative values indicate an increase in elastase production. ^$^ Elastase/protease inhibition because of ≥40% growth inhibition and, therefore, not regarded as quorum sensing inhibition.

**Table 5:** Effect of sponge-associated *Bacillus* species extracts on rhamnolipid production by *Pseudomonas aeruginosa* ATCC 27853. Percentage inhibition of rhamnolipid production was evaluated for extracts derived from cultures grown in medium Mannitol (Mann) and medium 5294 (5294) at concentrations ranging from 250 to 1000 µg/mL.

Extract Code	Percentage Rhamnolipid Inhibition (Orcinol Assay)							
	250 µg/mL		500 µg/mL *		750 µg/mL **		1000 µg/mL **	
	Mann	5294	Mann	5294 **	Mann	5294 *	Mann	5294
SP-AB2	5.54	11.92	14.18	44.65	21.08	74.22	21.30	14.69
SP1-AB4	12.22	45.17	17.41	50.30	17.44	54.68	9.02	56.81
SP2-AB7	86.03	47.53	79.47	50.95	80.71	51.27	85.73	51.49
SP2-W6	−40.52 **^#^**	−14.99 **^#^**	−34.96 **^#^**	14.17	3.14	4.23	21.80	−24.67 **^#^**
SP5-AB7	89.31	36.25	93.88	36.72	93.07	59.74	88.44	79.96
Furanone	26.96		23.90		24.37		28.90	
10% DMSO	26.90		28.39		25.75		20.88	

* Values carry a significance of *p* < 0.05. ** Values carry a significance of *p* < 0.01. ^**#**^ Negative values indicate an increase in rhamnolipid production.

**Table 6:** Percentage inhibition of virulence factors at 1000 µg/mL by 10 sponge-associated *Bacillus* species extracts fermented in medium Mannitol (Mann) and medium 5294 (5294), respectively.

Isolate Code	Percentage Virulence Factor Inhibition at 1000 µg/mL								
	Pyocyanin **	Pyoverdine **	Elastase LasB **	Protease (Casein Agar)	Protease (Azocasein) **	Rhamnolipid (CTAB agar)	Rhamnolipid (Orcinol) **	Swimming **	Swarming **
SP-AB2 (Mann)	32.63	38.43	25.66 *	10.50 *	9.19 *	54.73	21.30	6.72 *	13.29 *
SP-AB2 (5294)	36.21	26.89	2.35	21.44	53.51	39.64 *	14.69 *	37.35	42.41 *
SP1-AB4 (Mann)	37.57	29.37 *	50.32	30.39	41.02 *	16.71 *	9.02 *	37.35	5.7 *
SP1-AB4 (5294)	32.37	23.96 *	11.39 *	14.15 *	15.66 *	42.06	56.81	18.28 *	51.9 *
SP2-AB7 (Mann)	31.54	18.02 *	51.76	18.46 *	42.36	46.89	85.73	18.28 *	8.86 *
SP2-AB7 (5294)	19.70	30.92	46.01	17.96	59.93	39.64 *	51.49	37.94 *	29.11 *
SP2-W6 (Mann)	8.06 *	27.91	5.09 *	24.42 *	28.47	9.46 *	21.80	37.94 *	11.39 *
SP2-W6 (5294)	36.76	7.55	34.41	22.93 *	63.81	10.67 *	−24.67 *	28.24 *	45.57
SP5-AB7 (Mann)	32.69 *	8.17	71.32	21.44 *	38.77	67.41 *	88.44 *	28.24 *	29.11
SP5-AB7 (5294)	30.97	29.33 *	43.86	25.91	46.90	42.06 *	79.96	50.59 *	24.05
Cinnamaldehyde	79.53 ^$^	−932.37 ^$^	67.45 ^$^	54.26 ^$^	111.91 ^$^	54.13	28.90 ^**#**^	44.37 ^$^	−8.23 **^$^**
10% DMSO	0.59	18.09	12.81	6.52	12.66	−1.40	20.88	20.91	−49.37

* Higher virulence factor inhibition observed at a lower treatment concentration. ** Mean inhibition values at this concentration carry a significance of *p* < 0.01. ^$^ Inhibition because of ≥40% growth inhibition and, therefore, not regarded as quorum sensing inhibition. ^**#**^ Furanone and not cinnamaldehyde, used as positive control for this assay.

**Table 7:** Percentage biofilm reduction (%BFR) of sponge-associated *Bacillus* species extracts on initial adhesion and mature biofilm formation of *Pseudomonas aeruginosa* ATCC 27853. Testing was conducted with Mannitol (Mann) and 5294 (5294) media extracts at concentrations of 0.5, 1, 5, and 10 mg/mL.

Extract Code	Percentage Initial Adhesion Inhibition (%BFR)								Percentage Mature Biofilm Inhibition (%BFR)							
	0.5 mg/mL		1 mg/mL *		5 mg/mL **		10 mg/mL **		0.5 mg/mL		1 mg/mL *		5 mg/mL **		10 mg/mL **	
	Mann	5294 *	Mann	5294 **	Mann	5294	Mann	5294	Mann	5294 **	Mann *	5294 **	Mann	5294 **	Mann	5294 **
SP-AB2	−17.73 **^#^**	3.50	−17.92 **^#^**	13.82	78.77 ^$^	20.80	88.77 ^$^	40.96	35.96	11.78	40.43	12.11	40.67	12.29	86.28	12.33
SP1-AB4	2.84	6.97	31.13	16.54	58.89	69.39	73.58	81.41 ^$^	12.04	11.69	12.25	14.53	12.34	15.34	13.26	17.82
SP2-AB7	1.27	4.99	2.94	4.03	2.93	2.78	8.32	4.35	7.49	11.18	10.87	11.21	11.14	12.48	13.81	12.55
SP2-W6	−2.97 **^#^**	11.09	−3.16 **^#^**	19.39	−3.22 **^#^**	41.11	−2.36 **^#^**	41.84	10.06	12.56	9.75	13.63	7.53	14.24	7.97	14.74
SP5-AB7	21.26	19.54	31.43	17.59	66.51	14.47	71.70 ^$^	1.13	8.57	11.26	9.86	11.71	10.20	12.20	15.71	12.54
Cinnamaldehyde	−22.50 **^#^**		−21.12 **^#^**		70.25 ^$^		70.19 ^$^		38.85		38.55		45.39		46.43 ^$^	
10% DMSO	−23.36 **^#^**		−21.12 **^#^**		−18.47 **^#^**		−18.52 **^#^**		14.63		15.48		18.16		21.03	

* Values carry a significance of *p* < 0.05. ** Values carry a significance of *p* < 0.01. ^$^ Initial adhesion inhibition is due to ≥40% growth inhibition, and therefore not regarded as quorum sensing inhibition. ^**#**^ Negative values indicate an increase in initial adhesion. red = growth inhibition.

## Data Availability

Dataset available on reasonable request from the authors.

## Supplementary Materials

The following supporting information can be downloaded at https://www.mdpi.com/article/10.3390/antibiotics14101035/s1. Figure S1. Seven South African intertidal marine sponges; Figure S2. Growth of five selected *Bacillus* species isolates on GYM medium; Figure S3.1–S3.6. Pyocyanin, pyoverdine and growth inhibition following treatment with 10 sponge-associated *Bacillus* species extracts; Figure S4.1–S4.6. Elastase and growth inhibition following treatment with sponge-associated *Bacillus* species extracts; Figure S5.1–S5.6. Quantitative protease and growth inhibition following treatment with sponge-associated *Bacillus* species extracts; Figure S6.1–S6.6. Quantitative rhamnolipid and growth inhibition following treatment with sponge-associated *Bacillus* species extracts; Figure S7. Effect of crude sponge-associated *Bacillus* species medium Mannitol extracts on *Pseudomonas aeruginosa* ATCC 27583 (A) bacterial growth and (B) mature biofilm as quantified by crystal violet staining in a microtiter plate assay; Figure S8. Effect of crude sponge-associated *Bacillus* species medium 5294 extracts on *Pseudomonas aeruginosa* ATCC 27583 (A) bacterial growth and (B) mature biofilm as quantified by crystal violet staining in a microtiter plate assay; Table S1. Fourier transform infrared (FTIR) spectroscopy analysis of five sponge-associated *Bacillus* species medium Mannitol extracts and the medium Mannitol fermentation control; Table S2. Fourier transform infrared (FTIR) spectroscopy analysis of five sponge-associated *Bacillus* species medium 5294 extracts and the medium 5294 fermentation control; Table S3. Gas chromatography-mass spectrometry (GC-MS) analysis of 5 sponge-associated *Bacillus* species medium Mannitol extracts and the medium Mannitol fermentation control; Table S4. Gas chromatography-mass spectrometry (GC-MS) analysis of 5 sponge-associated *Bacillus* species medium 5294 extracts and the medium 5294 fermentation control; Table S5. Agar overlay assay results for 10 sponge-associated *Bacillus* species extracts, using bacterial biosensor *Chromobacterium violaceum* ATCC 12472; Table S6. Qualitative protease inhibition following treatment with sponge-associated *Bacillus* species extracts; Table S7. Qualitative rhamnolipid inhibition following treatment with sponge-associated *Bacillus* species extracts; Table S8. Inhibition of swimming motility following treatment with sponge-associated *Bacillus* species extracts; Table S9. Inhibition of swarming motility following treatment with sponge-associated *Bacillus* species extracts; Table S10. Summary of the highest observed inhibition of virulence phenotypes regulated by QS in *Pseudomonas aeruginosa* ATCC 27853 by sponge-associated *Bacillus* species isolates obtained at varying treatment concentrations; Table S11. Summary of notable compounds identified *via* GC-MS together with mechanism of action, *Pseudomonas aeruginosa* QS system/s, targeted and effect of phenotype.
