# Supplementary material for: Quorum Sensing Inhibition by Sponge-Associated Bacillus Species: Suppressing Pseudomonas aeruginosa Virulence Factors
Source: Antibiotics (Basel). 2025 Oct 16;14(10):1035. doi: 10.3390/antibiotics14101035 (PMC12562084; doi:10.3390/antibiotics14101035)
Supplement: Supplementary file 1 [file antibiotics-14-01035-s001.zip › antibiotics-3630286-supplementary.pdf]

Supplementary Material

Sponge-Associated *Bacillus* Species as Quorum Sensing Inhibitors: Disrupting *Pseudomonas aeruginosa* Virulence Factor Production

Carrie Shelouise Jacobs[1], Ryan Naicker[1], Hafizah Yousuf Chenia[1,2*]

[1] Discipline of Microbiology (Westville Campus), School of Life Sciences, University of KwaZulu-Natal, Private Bag X54001, Durban, South Africa
[2] Department of Microbiology, Stellenbosch university, Stellenbosch Campus, Private Bag X1, South Africa

Correspondence:
Hafizah Yousuf Chenia
Cheniah@ukzn.ac.za/ hchenia@sun.ac.za

Keywords: Sponge-bacteria, *Bacillus*, quorum sensing inhibition, *Pseudomonas*, anti-virulence

Sponge samples used for bacterial isolation

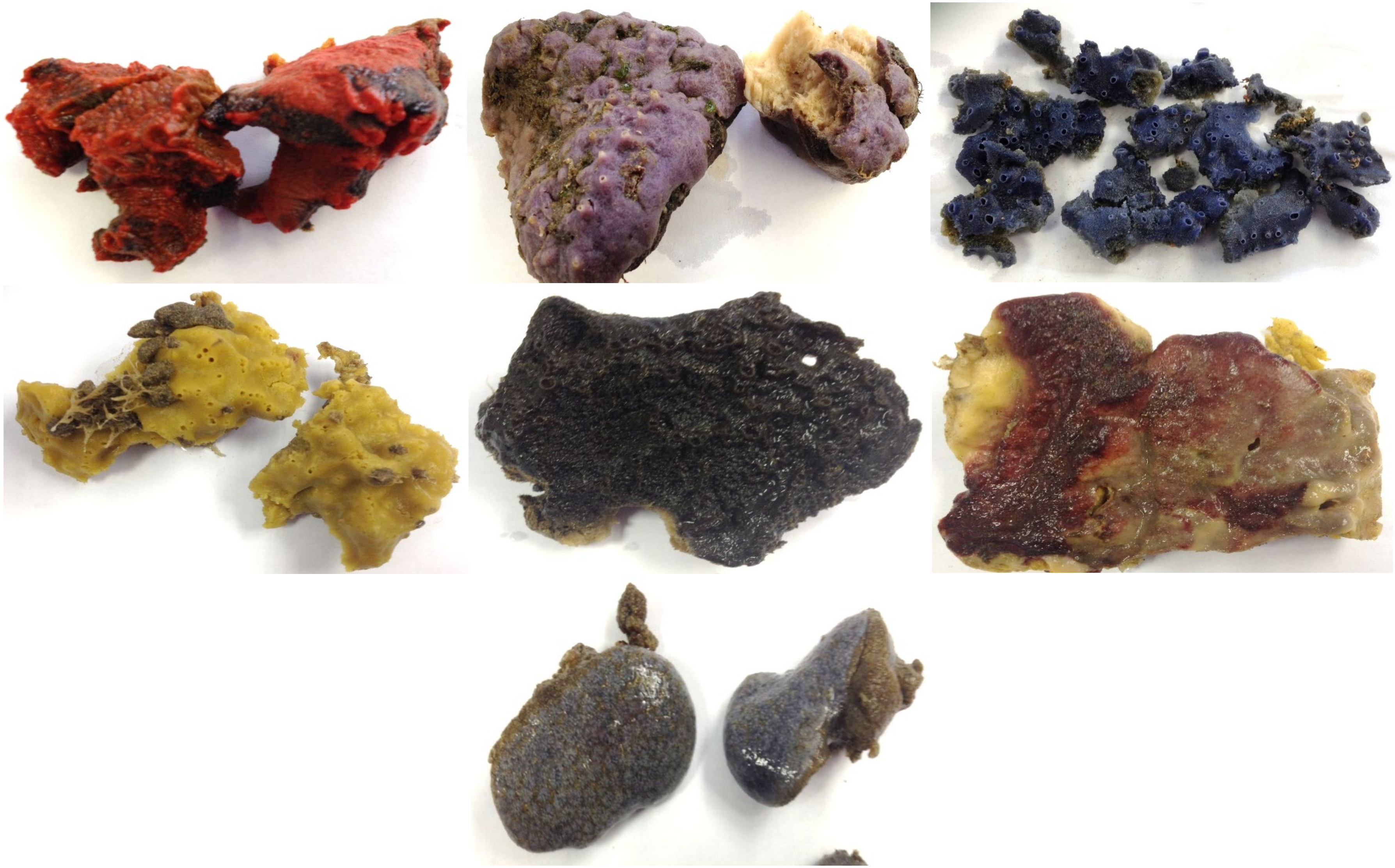

Figure S1. Seven South African intertidal marine sponges collected off the KwaZulu-Natal coast (29°59’58”S and 30°56’51”E) for isolation of sponge-associated bacteria. From top left to right: SP1, SP2, SP3, SP4, SP5, SP6, and SP7.

Bacterial isolates selected for study

A **B** **C** **D** **E**

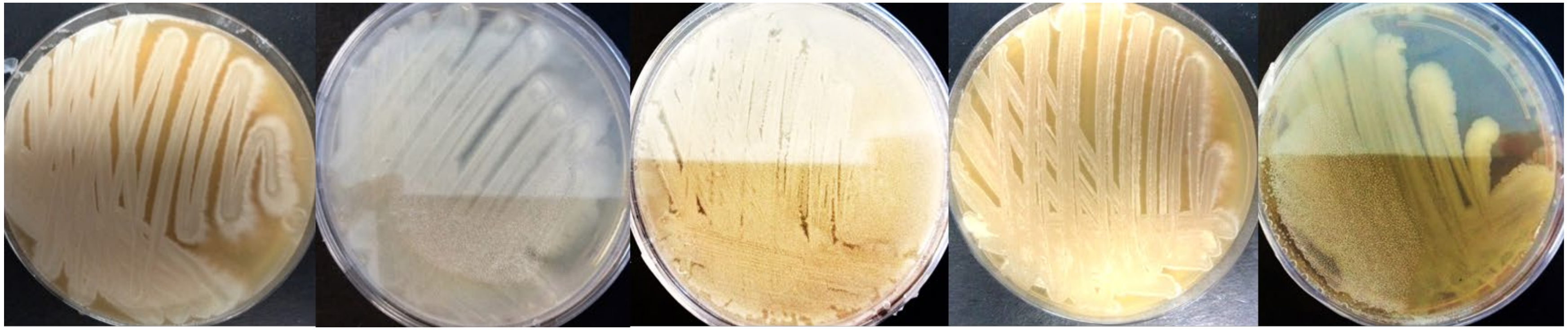

Figure S2. Growth of five selected *Bacillus* species isolates on GYM medium. (**A**) *Bacillus thuringiensis* SP-AB2, (**B**) *Bacillus cereus* SP1-AB4, (**C**) *Bacillus pumilus* SP2-W6, (**D**) *Bacillus mobilis* SP2-AB7 and (**E**) *Bacillus wiedmannii* SP5-AB7.

Table S1. Fourier transform infrared (FTIR) spectroscopy analysis of five sponge-associated *Bacillus* species medium Mannitol extracts and the medium Mannitol fermentation control.

Absorption range (cm^{-1})	medium Mannitol spectra peaks							
	**SP-AB2**	**SP1-AB4**	**SP2-AB7**	**SP2-W6**	**SP5-AB7**	**medium Mannitol control**	**Functional group**	**Compound class**
**3200 – 3550**	3350.97	3244.46	3355.85	3360.53	3217.35	*	O-H Stretching	Alcohol
		3200.43		3110.59	3106.81	3206.85	O-H Stretching	Alcohol
**2840 – 3000**	*	2921.62	*	2973.68	*	2924.66	C-H Stretching	Alkane
	2925.02	2852.12	2923.55	2924.92	2925.12	2859.32	C-H Stretching	Alkane
	*		2854.00	*	*		C-H Stretching	Alkane
**2550 – 2600**	2643.75						S-H Stretching	Thiol
**2330 – 2360**		2343.58	2326.35	2399.48			O=C=O Stretching	Carbon dioxide
**2100 – 2145**	2118.22	2120.87	2120.68	2118.59	2106.77		C≡C Stretching	Alkyne
	2089.99	2103.30	2092.34	2095.30			C≡C Stretching	Alkyne
**1650 – 2000**	1877.23	1987.61			1857.56		C-H Bending (overtone)	Aromatic compound
		1881.03					C-H Bending (overtone)	
**1680 – 1725**	1706.05	*	1705.36	1705.83	1709.44	1725.30	C=O Stretching	Ester/Ketone
**1626 – 1662**	1673.36	1656.49	1657.80	1656.44	1639.44	1667.70	C=C Stretching	Alkene
**1390 – 1310**		1320.84					O-H Bending	Phenol
**1375 – 1460**	1452.95	1451.61	1452.34	1449.94	1450.90	1450.64	C-H Bending (scissoring)	Alkane
	1416.84			1417.24	1416.57	1379.33	C-H Bending (scissoring)	Alkane
**1360 – 1380**	1376.67	1374.10	1374.06	1373.63	1378.00		CH2 and CH3 deformation	Alkane
**1250 – 1310**			1276.27			1274.65	C-O Stretching	Aromatic Ester
**1000 – 1250**	1232.09	*	1172.47	1234.76	1230.86	*	C-O Stretching	Ether
	1126.46	1193.71	1086.88	1173.11	1123.86	1125.88	C-O Stretching	Ether
	1094.87	1077.49	1063.47	1088.69	*	1075.81	C-O Stretching	Ether
	1042.63	1017.83	1011.37	1008.63	1004.38		C-O Stretching	Ether
**910 -990**	925.36	927.42	925.49	904.89	942.34	977.25	C-H Bending (Out-of-Plane)/C-H wagging	Alkene/Alkyl chains
**880 ± 20**		872.40	901.55				C-H Bending (Out-of-Plane)	Aromatic compound (1,2,4-trisubstituted)
**810 ± 20**	821.30			820.21	821.81		C-H Bending	Aromatic compound (1,4-disubstituted)
**750 ± 20**	744.54			758.34	744.21	739.11	C=C Bending (disubstituted; cis)	Aromatic compound (monosubstituted)
**665 -730**	698.14	707.22	718.54	702.36	699.44		C=C Bending (disubstituted; cis)	Alkene
**515 – 690**						659.49	C-Br Stretching	Halo Compound

Key: (*) absorption peaks (**cm**$^{-1}$) extrapolated from FTIR graphs.

Table S2. Fourier transform infrared (FTIR) spectroscopy analysis of five sponge-associated *Bacillus* species medium 5294 extracts and the medium 5294 fermentation control.

Absorption range (cm^{-1})	medium 5294 spectra peaks							
	**AB2-SP**	**SP1-AB4**	**SP2-AB7**	**SP2-W6**	**SP5-SP7**	**medium 5294 control**	**Functional group**	**Compound class**
**3300 -3400**	3342.98		3342.86		3343.01	3337.24	N-H Stretching (Symmetric)	Primary amine
**3200 - 3300**	3221.66		3208.07		3205.39	3210.19	N-H Stretching (Asymmetric)	Primary amine
**2840 - 3000**	*	2920.95	2933.82	2922	2933.03	2934.63	C-H Stretching	Alkane
	2930.84	2854.1	*	2 855.49	*	2877.36	C-H Stretching	Alkane
	*						C-H Stretching	Alkane
**1680 - 1725**	1715.55			1740.8	*	1724.41	C=O Stretching	Ester/Ketone
**± 1650**	1654.64						C=O Stretching (Associated)	Primary amide
**1580 - 1650**	*		1603.09	1641.81	1588.59	1608.33	N-H Bending	Amine
**1375 - 1460**	1422.47	1454.34	1447.97	1455.17	1450.7	1449.11	C-H Bending (Scissoring)	Alkane
**1360 - 1380**	*	1367.91	*	1370.07	*		CH2 and CH3 deformation	Alkane
**1000 - 1250**	1223.88	1220.09	1226.66	*	1224.47	1233.97	C-N Stretching/C-O Stretching	Amine/Ether
	1153.46	1102.63	1156.59	1163.41	1155.75	1159.77	C-N Stretching/C-O Stretching	Amine/Ether
	1092.73	*	*	*	1106.83	1104.68	C-N Stretching/C-O Stretching	Amine/Ether
	1038.41	*	1043.53		1042.48	1037.39	C-N Stretching/C-O Stretching	Amine/Ether
**750 ± 20**	763.13			720.42			C-H Bending (Out-of-Plane)	Aromatic compound (monosubstituted)
**500 – 690**	581.67		580.74		585.38	581.43	C-Br Stretching/C-I Stretching	Halo Compound
	521.53		518.52		525.50	521.62	C-Br Stretching/C-I Stretching	Halo Compound

Key: (*) absorption peaks (cm^{-1}) extrapolated from FTIR graphs.

Table S3. Gas chromatography-mass spectrometry (GC-MS) analysis of 5 sponge-associated *Bacillus* species medium Mannitol extracts and the medium Mannitol fermentation control.

Compounds (n=300)*	Classification	medium Mannitol extracts											
		AB2-SP (n=80)		SP1-AB4 (n=46)		SP2-AB7 (n=68)		SP2-W6 (n=101)		SP5-AB7 (n=81)		Medium Mannitol Control (n= 20)	
		RT (min)	%Area	RT (min)	%Area	RT (min)	%Area	RT (min)	%Area	RT (min)	%Area	RT (min)	%Area
1,2-Dihydro-8-hydroxylinalool [a]	Alcohol (n=16)	15.554	0.13										
1,3,5-Pentanetriol, 3-methyl- [d]								11.512	0.12				
1-Dodecanol, 2-octyl- [c]						31.810	0.19						
1-Hentetracontanol [a]		16.117	0.19										
1-Heptanol, 2-propyl- [a]		13.800	0.14										
1R,2R,3S,5R)-(-)-2,3-Pinanediol [a]		15.605	0.11										
3-Hexanol, 3,5-dimethyl- [d]								16.020	0.16				
5,7-Dodecadiyn-1,12-diol [d]								21.400	0.69				
								21.805	0.06				
5,9-Dimethyl-3-decanol [d]								12.182	0.25				
Cyclohexanol, 2-methyl-3-(1-methylethenyl)-, (1.alpha.,2.alpha.,3.alpha.)- [b]				23.780	0.05								
n-Heptadecanol-1 [c]						19.257	0.21						
n-Pentadecanol		13.658	0.19			13.665	0.18						
n-Nonadecanol-1 [e]										19.259	0.86		
n-Tridecan-1-ol [e]										13.665	0.37		
Tridecanol, 2-ethyl-2-methyl-		15.414	0.53			15.429	0.40						
Z-11-Pentadecenol [e]										23.862	0.30		
1,2,4-Butanetriol [c]	Alcohol(polyol) (n=1)					5.486	2.27						
2-Methyl-oct-2-enedial [e]	Aldehyde (n=3)									13.935	0.52		
Cinnamaldehyde, .alpha.-pentyl- [d]								14.160	0.43				
E-14-Hexadecenal [b]				13.665	0.54								
Aspidospermidin-17-ol, 1-acetyl-19,21-epoxy-15,16-dimethoxy-	Alkaloid (n=2)	15.565	0.12							16.010	0.21		
Cevane-3,4,6,7,14,15,16,20-octol, 4,9-epoxy-, 6,7-diacetate 3-(2-hydroxy-2-methylbutanoate) 15-(2-methylbutanoate), [3.beta.(S),4.alpha.,6.alpha.,7.alpha.,15alpha.,] [c]						13.439	0.77						
						13.871	0.54						
2,3-Dimethyldodecane [c]	Alkane (n= 44)					16.121	0.12						
2-Methylhexacosane		17.505	0.24	21.256	0.38	16.781	0.17			15.428	0.42		
		14.343	0.52							14.349	0.97		
3,3-Dimethylnonadecane [b]				17.160	0.34								
4-Methyldocosane [c]						14.940	0.30						
5-Butyl-5-ethylpentadecane [c]						16.902	0.18						
Cyclohexane, 1,2,3,5-tetraisopropyl- [e]										31.805	0.40		
Decane, 2,3,8-trimethyl [e]										16.324	0.21		

Dodecane, 2,6,11-trimethyl-							17.088	0.53	17.181	0.29		
Dodecane, 2-methyl-6-propyl- [f]											19.013	0.81
Dodecane, 4-methyl- [e]									15.181	0.75		
Dodecane, 5,8-diethyl- [a]	16.485	0.20										
Dodecylcyclohexane [e]									18.830	0.48		
Dotetracontane [b]			17.975	2.03								
Eicosane	15.946	0.79	15.176	0.71	15.185	0.45	25.415	0.26	15.962	1.48		
	19.376	0.85	15.953	2.13	17.382	0.92			17.380	2.54		
	22.168	0.79	19.371	5.47	19.402	0.76			19.397	2.54		
	26.115	0.71	26.108	3.80	22.206	0.53			22.197	1.88		
	31.745	0.52	29.425	3.45								
			31.722	2.71								
Heneicosane			17.365	5.11			19.002	0.50				
Heptadecane	14.715	1.82	14.720	4.38	14.726	1.52	14.545	0.45	14.722	4.46		
			15.876	5.19	15.886	1.20	15.658	0.22				
Heptadecane, 2,6,10,15-tetramethyl-	13.358	0.23	13.734	0.80					15.886	3.60		
	13.725	0.63	22.169	4.42					12.708	0.35		
	15.870	1.42							13.363	0.24		
	17.363	1.19							13.734	1.99		
Heptadecane, 2-methyl-	14.419	0.23	14.415	0.80	14.427	0.28			14.426	0.53		
			15.417	0.40								
Heptadecane, 3-methyl-	15.508	0.41	15.510	0.49					15.521	0.36		
Heptadecane, 4-methyl-	15.338	0.36			15.350	0.31			15.346	0.25		
Heptadecane, 7-methyl- [b]			21.007	0.19								
Heptadecane, 9-hexyl- [e]									16.905	0.44		
Hexacontane [b]			20.345	1.06								
Hexadecane [c]					13.732	0.65						
Hexadecane, 2,6,10,14-tetramethyl- [c]					15.961	0.65						
Hexadecane, 4-methyl-	14.283	0.35	14.282	0.29	14.287	0.22			14.288	0.65		
					16.676	0.27						
Nonadecane, 4-methyl- [a]	18.420	0.41										
Nonadecane, 9-methyl-									16.439	0.61		
Nonane, 5-butyl- [b]			16.429	0.63								
Octadecane, 2-methyl- [e]									16.775	0.31		
Octadecane, 3-methyl-	16.888	0.27	16.890	0.84								
Octadecane, 4-methyl-			16.660	0.28					14.944	0.27		
									16.683	0.36		
Octadecane, 5-methyl	18.080	0.16	14.225	0.17					26.157	1.25		
	16.556	0.12										
Octadecane, 6-methyl- [c]					15.230	0.16						
Pentadecane, 2,6,10,14-tetramethyl- [c]					14.183	0.54						
Pentadecane, 2,6,10-trimethyl-	14.174	0.72	14.176	0.96	14.183	0.54			14.180	1.99		
Pentadecane, 3-methyl- [e]									13.436	0.17		
Pentadecane, 8-hexyl- [c]					14.354	0.47						
Tetracontane, 3,5,24-trimethyl- [d]							14.017	0.12				
Tetradecane, 4,11-dimethyl-			15.338	0.45					14.060	0.82		

						17.168	**0.21**						
**Tetradecane, 4-ethyl-**				15.261	**0.79**					15.265	**0.33**		
**Tetradecane, 4-methyl-** [a]		16.438	**0.42**										
**Tetradecane, 5-methyl-**		14.218	**0.20**			15.273	**0.29**			14.225	**0.52**		
		15.259	**0.35**										
**Tetratetracontane** [b]				16.761	**0.42**								
**2,6,10,14-Tetramethyl-7-(3-methylpent-4-enylidene) pentadecane**		16.042	**0.27**							25.908	**0.26**		
**2,6,10-Trimethylundeca-1,3-diene** [e]	Alkene (n=4)									23.944	**0.52**		
**2-Hexadecene, 3,7,11,15-tetramethyl-, [R-[R*,R*-(E)]]-** [b]				18.445	**0.86**								
**5-Methyl-Z-5-docosene** [b]				15.665	**0.08**								
**Ethanol, 2-(pentyloxy)-, acetate** [d]	Alkoxy acetate (n=1)							9.819	**0.19**				
**3,5-Dodecadiyne, 2-methyl-** [d]								19.480	**0.16**				
**Trans-3-Undecene-1,5-diyne**	Alkyne (n=2)							17.577	**0.14**			18.921	**0.86**
								17.940	**0.38**				
								20.601	**0.17**				
**Formamide, N,N-dibutyl-** [d]	Amide (n=1)							10.460	**0.24**				
**2,6-Octadien-1-amine, 3,7-dimethyl-** [b]	Amine (n=2)			23.755	**0.02**								
**2-Propen-1-amine, N,N-dipropyl-** [c]						11.369	**0.22**						
**Norvaline, 3-hydroxy-** [c]						7.495	**1.01**						
**Isovaline, 3-hydroxy-**	Amino acid (n=2)	6.240	**0.35**			6.303	**22.52**						
		6.371	**0.14**										
**Arginine, N(2)-p-toluenesulfonyl-, t-butyl ester** [a]	Amino acid derivative (n=2)	18.475	**0.07**										
**Beta.-Alanine, n-propargyloxycarbonyl-, heptyl ester** [e]										15.225	**0.19**		
**2,3-Anhydro-d-galactosan** [e]	Anhydro sugar (n= 1)									13.965	**0.66**		
										18.890	**0.18**		
**4-Chloro-2-methylbenzyl alcohol** [e]	Aromatic alcohol (n=2)									14.465	**0.17**		
**Benzeneethanol,.alpha.,.alpha.,.beta.-trimethyl-** [d]								13.753	**0.27**				
**Benzaldehyde, 3,4-dimethoxy-2-nitro-** [f]	Aromatic aldehyde (n=1)											18.626	**1.13**
**3-Amino-3-(4-isopropoxy-phenyl)-propionic acid** [c]	Aromatic amino acid (n=1)					13.385	**0.74**						
**Benzeneacetic acid**	Aromatic carboxylic acid (n=2)	10.598	**58.73**					10.313	**8.78**	10,346	**13.69**		
**p-Undecyloxybenzoic acid** [e]										14.020	**0.13**		
**1,4-Dicyano-2-(5-hexenyl)benzene** [d]	Aromatic compound (n=1)							19.896	**0.16**				
**Benzoic acid, 2-hydroxy-, 2-methylbutyl ester** [d]	Aromatic ester (n=8)							13.444	**0.18**				
**Benzoic acid, 2-hydroxy-, phenylmethyl ester** [d]								17.008	**0.44**				
**Cyclohexanecarboxylic acid, 4-butyl-, 4-propylphenyl ester** [c]						14.110	**0.10**						

Hydroxy(4-benzyloxy-3-chlorophenyl)acetic acid,methyl ester [a]		14.888	0.88										
n-Hexyl-4-hydroxybenzoate [e]										13.855	0.45		
n-Octyl-4-hydroxybenzoate [e]										13.805	1.14		
Octanoic acid, 3,5-difluorophenyl ester [e]										16.515	0.50		
Isoamyl salicylate [d]								13.027	0.06				
N-[4-Chloro-2-nitrophenyl]piperidine [f]	Aryl amine (n=1)											18.570	0.65
l-(+)-Ascorbic acid 2,6-dihexadecanoate [d]	Ascorbate ester (n=1)							17.695	0.81	18.745	1.64		
2-Phenyl-l-p-toluenesulfonylaziridine [a]	Aziridine derivative (n=1)	14.571	0.59										
Benzoic acid [a]	Benzoic acid derivatives (n=1)	9.373	0.34										
2,5-Hexanedione, 3,4-dihydroxy-3,4-dimethyl- [c]	Beta-hydroxy diketone (n=1)					6.433	15.38						
2,2,6-Trimethyl-bicyclo[4.1.0]hept-1-yl)-methanol [a]	Bicyclic alcohol (n=1)	15.050	0.25										
2,2'-Bifuran, octahydro- [d]	Bicyclic ether (n=1)							12.579	0.04				
1-Chloro-3-iodotricyclo[3.3.1.0(3,7)]nonan-9-one [d]								17.211	0.36				
6-Hydroxy-7-methyl-9-oxabicyclo[3.3.1]nonan-2-one [a]	Bicyclic ketone (n=3)	16.205	0.08					14.925	0.22				
Bicyclo[4.2.0]octan-7-one, 8,8-dichloro-1-methyl- [d]								15.055	0.15				
								15.144	0.20				
								15.282	0.15				
Trans-6-methylbicyclo[4.4.0]decan-1-ol-3-one [c]						14.485	0.30						
2-Methyl-4,5-tetramethylene-5-ethyl-2-oxazoline [c]	Bicyclic oxazoline (n=1)					13.790	0.10						
(R-(R*,R*))-4-(1,5-Dimethylhexyl)-1-cyclohexenecarboxylic acid [b]				15.585	0.33								
Cyclopropaneacetic acid, 2-hexyl-	Carboxylic acid (n=3)			12.496	6.90	12.493	1.46			12.485	1.50		
Dodecanoic acid		13.521	0.26	13.525	0.64					13.521	0.34		
Propanol, 3-(1,3,5-trimethyl-2,6-dioxocyclohexyl) [c]	Cyclic ketone (n=1)					16.470	0.10						
Cyclohexanemethanol, .alpha.,.alpha.,4-trimethyl- [d]	Cycloalcohol ((n=3)							14.711	0.50				
d-Menthol [d]								13.820	0.12				
1,1,3,6-Tetramethyl-2-(3,6,10,13,14-pentamethyl-3-ethyl-Pentadecyl)cyclohexane [c]	Cycloalkane (n=2)					15.080	0.35						
Tridecane, 6-cyclohexyl- [b]				21.050	0.09								
2-Butyne-1,4-diamine, N,N,N',N'-tetraethyl- [c]	Diamine (n=1)					18.830	0.12						

**Malic Acid [c]**	Dicarboxylic acid (n=2)					7.929<br>8.110<br>8.318	**8.84**<br>**8.13**<br>**3.28**						
**Methylmalonic acid [a]**		8.624	**0.82**										
**Oxalic acid, decyl 3,5-difluorophenyl ester**	Dicarboxylic acids and derivatives (n=1)	18.196	**0.32**			26.176	**0.27**						
**1,1-Cyclopropanedicarboxylic acid, 2-ethenyl-, diethyl ester [e]**										13.845	**0.54**		
**Fumaric acid, 2-chloropropyl pentadecyl ester [e]**										16.116	**0.32**		
**Fumaric acid, decyl 2-ethylhexyl ester [c]**	Diester (n=5)					15.000	**0.36**						
**Hexadecanoic acid, 1,4-butanediyl ester [c]**						16.394	**0.31**						
**Undecanedioic acid, 4-oxo-, dimethyl ester [b]**				19.460	**0.03**								
**2-Methoxycarbonylmethyl-4-hydroxyl-5-methyl-2,3-dihydrofuran [c]**	Dihydrofuran derivative (n=1)					15.630	**0.05**						
**2,4-Hexanedione, 3,5-dimethyl-1-phenyl [d]**								20.979	**0.60**				
**3-Octene-2,6-dione, 5,5,7-trimethyl-, (E)- [d]**	Diketone (n=3)							11.952	**0.06**				
**5,10-Tetradecanedione [c]**						26.156	**0.20**						
**1-Hexadecanol, acetate [c]**						14.844	**0.38**						
**2-(5-Methyl-5-vinyltetrahydrofuran-2-yl)propan-2-yl 2-methylbutanoate [b]**				17.825	**0.17**								
**2,2,4-Trimethyl-1,3-pentanediol diisobutyrate [d]**								10.608<br>11.650<br>11.765	**0.10**<br>**0.05**<br>**0.08**				
**2,2-Dimethylpropanoic acid, 4-hexadecyl ester [e]**										20.359	**0.38**		
**2,5-Octadecadiynoic acid, methyl ester**		14.505	**0.16**					14.855	**0.18**				
**2H-Benzazepine-2-carboxylic acid, 1,3,4,5-tetrahydro-5-methyl-1-oxo-3-spirocyclohexane-, methyl ester [f]**	Ester (n=59)											18.260	**0.93**
**3-Chloropropionic acid, 4-hexadecyl ester [c]**						14.576	**0.26**						
**4-Chlorobutyric acid, cyclohexylmethyl ester [c]**						16.058	**0.42**						
**5-Chloropentanoic acid, 2-butyl ester [e]**										13.895	**0.59**		
**5-methylhexanoic Acid, 2,2,2-trifluoroethyl ester [e]**										31.825	**0.30**		
**7-Methyl-Z-tetradecen-1-ol acetate [b]**				20.520	**0.19**								
**9,12-Octadecadienoic acid, methyl ester [d]**								23.137	**0.93**				
**Acetic acid, 4-methoxy-1,4,4a,5,8,8a-hexahydronaphthalen-1-yl ester [a]**		18.150<br>18.295	**0.22**<br>**0.17**										

Acetic acid, chloro-, hexadecyl ester [b]			15.798	1.08								
Adipic acid, 2-butoxyethyl heptadecyl ester [a]	13.875	0.03										
Benzeneacetic acid, 2-butyl ester [a]	12.923	0.26										
Benzeneacetic acid, 3-tetradecyl ester [a]	13.423<br>14.660<br>16.297	0.28<br>0.43<br>0.20										
Benzeneacetic acid, 4-pentadecyl ester [a]	13.990	0.55										
Benzeneacetic acid, 4-tridecyl ester [a]	13.825	0.08										
Benzeneacetic acid, 6-ethyl-3-octyl ester [a]	15.174	1.04										
Benzeneacetic acid, ethyl ester [a]	13.185	4.26										
Bromoacetic acid, 2-pentadecyl ester [e]									14.844	0.52		
Chloroacetic acid, pentadecyl ester [c]					15.803	0.94						
Cyclohexaneacetic acid, 2-oxo-, methyl ester [e]									16.055	0.34		
Cyclohexanecarboxylic acid, hexyl ester [e]									14.579	0.63		
Decanoic acid, 2-propenyl ester [a]	14.995	0.32										
Dibutyl adipate [d]							14.808	0.35				
Dimethylmalonic acid, monochloride, decyl ester [a]	32.410	0.24										
Dodecanoic acid, isooctyl ester							21.659	1.24			21.650	2.42
Ethyl 3-oxoheptanoate [a]	8.750	0.13										
Formic acid, 4-chloro-3-methylbut-2-enyl ester [d]							16.066	0.18				
Glutaric acid, 3,5-difluorophenyl isobutyl ester [d]							17.515	0.20				
Glutaric acid, di(isobutyl) ester [d]							13.347	2.90				
Glutaric acid, isobutyl undecyl ester [f]											13.351	1.10
Heptadecafluorononanoic acid, tetradecyl ester [a]	14.817	0.26										
Heptanoic acid, octyl ester [a]	18.282	0.21										
Hexanedioic acid, bis(2-methylpropyl) ester							12.833<br>14.408<br>19.728	0.13<br>6.06<br>0.13			14.392	6.84
i-Propyl 12-methyl-tridecanoate [a]	16.193	0.13										
Isopropyl myristate					16.203	0.44	15.962	0.30	16.196	0.57		
Isopropyl palmitate [d]							19.526	0.30				
Methoxyacetic acid, 4-tridecyl ester [a]	17.151	0.44										
Methyl 14-methyl-eicosanoate	17.830	0.26							17.846	0.28		
Methyl 3,5-tetradecadiynoate [d]							18.630	0.96				
Nona-2,3-dienoic acid, ethyl ester [b]			17.005	0.14								
Oxalic acid, 3,5-difluorophenyl tetradecyl ester [d]							15.739	0.23				

**Pentanoic acid, 2,2,4-trimethyl-3-carboxyisopropyl, isobutyl ester** [d]								13.481	**0.36**				
**Pentanoic acid, 2,4-dimethyl-3-oxo-, methyl ester** [d]								11.194	**0.41**				
**Pentyl phenylacetate** [e]										10.680	**0.25**		
**Phenylacetic acid, 2-chlorophenyl ester** [a]		16.612	**0.94**										
**Phenylacetic acid, 2-ethoxyethyl ester** [a]		13.246	**2.30**										
**Phenylacetic acid, 2-ethylhexyl ester** [a]		14.780	**0.25**										
**Phenylacetic acid, 4-cyanophenyl ester** [a]		30.779	**1.01**										
**Propanoic acid, 2-methyl-, 2,2-dimethyl-1-(2-hydroxy-1-methylethyl)propyl ester** [d]								11.025	**5.94**				
**Propanoic acid, 2-methyl-, 3-hydroxy-2,4,4-trimethylpentyl ester** [d]								11.288	**6.83**				
**Succinic acid, 2-heptyl isobutyl ester** [d]								12.300	**1.50**				
**Trifluoroacetic acid, pentadecyl ester** [d]								15.579	**0.21**				
**Hexadecane, 1-methoxy-13-methyl-** [a]		16.243	**0.12**										
**Hexane, 1-(hexyloxy)-4-methyl-** [b]				16.505	**0.20**								
**Eicosyl acetate** [d]	Fatty acid ester (n=1)							25.857	**0.26**				
**Streptovitacin** [a]	Glutaramide derivative (n=1)	15.685	**0.13**										
**Glycerol 1,2-diacetate** [d]	Glycerol derivative (n=1)							10.801	**0.07**				
**3-Chloropropionic acid, octadecyl ester** [e]										14.895	**0.18**		
**Acetic acid, chloro-, hexadecyl ester** [e]										15.804	**1.52**		
**Eicosyl pentafluoropropionate** [e]	Halide ester (n=5)									17.521	**0.41**		
**Phenylacetic acid, 2-bromo-4-fluorophenyl ester** [a]		17.257<br>30.559	**0.33**<br>**0.35**										
**Trichloroacetic acid, 4-hexadecyl ester** [b]				14.565	**0.27**								
**2-Bromotetradecane** [b]				14.349<br>18.546	**0.46**<br>**0.85**								
**Cyclopropane, 1,1-dichloro-2,2,3-triethyl-** [f]	Haloalkane (n=5)											16.869<br>18.480	**0.87**<br>**0.91**
**Hexadecane, 1-iodo-**		16.768	**0.47**			15.522	**0.39**						
**Pentane, 1,1,1,5-tetrachloro-** [d]								20.836	**0.18**				
**Tetrapentacontane, 1,54-dibromo-** [e]										21.288	**0.28**		
**(4Z)-5-Chloro-3,4-dimethyl-2,4-heptadiene** [d]								18.755	**0.42**				
**1-Heptene, 5,7,7,7-tetrachloro-** [d]	Haloalkene (n=4)							19.155	**0.22**				
**7-Heptadecene, 1-chloro-** [d]								20.401	**0.11**				
**cis-1-Chloro-9-octadecene**		15.790	**0.70**	19.241	**0.16**								
**4-Heptadecyne, 1-chloro-** [d]	Haloalkyne (n=3)							15.227	**0.08**				

6-Heptadecyne, 1-chloro- [d]								16.820	0.38				
Chloro-4-decyne [d]								19.065	0.26				
2,3-Diazabicyclo[3.2.0]hept-2-ene, 1,6,6,-trifluoro-4-spirocyclopropane [e]	Heterocyclic compound (n= 1)									14.670	0.16		
Pyrrolo[1,2-a]pyrazine-1,4-dione, hexahydro-3-(2-methylpropyl)-	Heterocyclic compound (diketopiperazine) (n=1)					16.548	0.89	17.818	1.02	16.569	0.80		
						18.090	2.10	18.224	4.42	18.089	4.66		
						18.440	2.76			18.445	6.64		
2-Methyl-6-phenyl-5,6-dihydro-4H-1,3-oxazine [d]	Heterocyclic organic compound (oxazine)(n=1)							12.960	0.09				
2,4-Imidazolidinedione, 5-(2-methylpropyl)-, (S)-								13.644	0.22			13.700	0.67
5-Isopropyl-2,4-imidazolidinedione [d]								13.700	0.12				
5-Methylenehydantoin [c]	Hydantoin (n=3)					10.329	0.24						
						11.110	0.43						
						11.167	0.33						
						11.298	0.27						
2-(4-Chlorophenoxy)-N'-(2-propoxybenzylidene)acethydrazide [e]	Hydrazide organic compound (n= 1)									18.020	0.52		
Bicyclo[4.1.0]heptane-7-carbohydrazide, N2-(3-allyl-2-hydroxybenzylideno)- [f]	Hydrazone (n=1)											14.925	0.65
Cyclohexanone, 3-methyl-, (2,4-dinitrophenyl)hydrazone [d]	Hydrazone derivative (n=1)							19.655	0.04				
1H-Indole, 3-methyl- [a]	Indole (n=1)	11.785	0.13										
Benzenepropanoic acid, .alpha.-oxo- [a]	Keto acid (n=1)	14.076	0.51										
1-Penten-3-one, 1-(2,6,6-trimethyl-2-cyclohexen-1-yl)-, (E)- [d]	Ketone (n=5)							12.795	0.12				
2-Butanone, 4-(2,6,6-trimethyl-2-cyclohexen-1-ylidene)- [a]		14.050	0.42										
4-(2,4-Dimethylcyclohexyl)butan-2-one [a]		16.145	0.07										
5,9-Dimethyl-2-(1-methylethyl)cyclodecane-1,4-dione		32.485	0.18							32.655	3.56		
9-Octadecanone [c]						14.060	0.13						
4-O-Methyl-2,3-O-benzal-d-mannosan [a]	Monosaccharide derivative (n=1)	15.661	0.30										
Propanedinitrile, bicyclo[3.3.1]non-9-ylidene- [d]	Nitrile (n=2)							15.095	0.15				
								15.428	0.15				
								15.881	0.18				
								21.065	0.16				
Propionitrile, 3-[1-[4-[1-(2-cyanoethoxy)cyclohexyl]buta-1,3-diynyl]cyclohexyloxy]- [d]								16.887	0.59				
								18.922	0.59				
Acetic acid, 2-(1-buten-3-yl)-2-nitro-, ethyl ester [a]	Nitro ester (n=2)	32.360	0.32										
Nitric acid, decyl ester [e]										13.995	0.51		
5-Azido-desoxythymidine [e]	Nucleoside analogues									17.480	0.04		

**1,5-Diacetyl-3,7-bis(3-chloropropionyl)-octahydro-1,3,5,7-tetraazocine [c]**						15.695	**0.12**						
**1,6-Octadiene, 3-ethoxy-3,7-dimethyl- [d]**								12.652	**0.24**				
**1-Cyclohexyl-3-nitropentan-1-ol [e]**										17.470	**0.18**		
**2H-Pyran-2-carboxylic acid, 3,6-dihydro-6-methoxy-, [4-(1-methylethyl)cyclohexyl]methyl ester [c]**						13.995	**0.35**						
**2(1H)-Naphthalenone, 5-[2-(3-furanyl)ethyl]octahydro-1,5,6,8a-tetramethyl-, [1R-(1.alpha.,4a.beta.,5.beta.,6.alpha.,8a.beta.)]- [e]**										14.652	**0.33**		
**4(1H)-Isobenzofuranone, hexahydro-3a,7a-dimethyl-, cis-(.+/-.)- [b]**				18.485	**0.19**								
**4-(3-Acetylaminopropyl)cyclohexanol [e]**	Organic compound (n=13)									10.530	**1.44**		
**6H-Furo[2',3':4,5]oxazolo[3,2-a]pyrimidin-6-one, 2,3,3a,9a-tetrahydro-3-hydroxy-2-(hydroxymethyl)-, [2R-(2.alpha.,3.beta.,3a.beta.,9a.beta.)] [a]**		23.900	**0.02**										
**Acetyl methyl carbinol dimer [c]**						8.925	**0.13**						
**Benzoic acid, 9-[2-(adamantan-2-yliden-methoxymethyl)-phenyl]-6-oxo-6H-xanthen-3-yl ester [d]**								15.817	**0.19**				
**Benzoic alcohol, 3-fluoro-4-methoxy-, acetate [c]**						15.665	**0.12**						
**Propanedinitrile, cyclooctylidene- [a]**		16.335	**0.12**										
**trans-(2-(4-Cyanophenyl)-5-pyrimidinyl)-4-hexylcyclohexane-1-carboxylate [e]**										14.495	**0.47**		
**Sulfurous acid, decyl 2-propyl ester [b]**				17.511	**0.33**								
**Sulfurous acid, pentyl undecyl ester [c]**	Organosulphur compound (n= 3)					16.450	**0.23**						
**Thiourea, N-(4-fluorophenyl)-N'-(2-methyl-2-propenyl)- [e]**										18.645	**0.11**		
**1,2,4-Oxadiazole, 3-(1,3-benzodioxol-5-yl)-5-(2-ethoxyphenyl)- [e]**	Oxadiazole derivative									31.735	**0.29**		
**6-tert-Butyl-2,4-dimethylphenol [d]**	Phenol (n=1)							11.439	**0.18**				
**Phosphonic acid, isopropyl-, ethyl (2-methoxy-1-methylethyl) ester [a]**	Phosphonic ester (n=1)	14.460	**0.18**										
**1,2-Benzenedicarboxylic acid, bis(2-methylpropyl) ester**								16.684	**19.80**			16.614	**57.04**
**Bis-(3,5,5-trimethylhexyl) phthalate [a]**		32.455	**0.12**										
**Bis(tridecyl) phthalate**	Phthalate ester (n=15)	18.538	**0.32**					13.560	**0.22**				
**Diisooctyl phthalate**				32.556<br>32.605	**4.83**<br>**14.80**	32.550	**1.25**	30.650	**5.74**				

Isophthalic acid, 2-methylbutyl octyl ester [c]						32.465	2.19						
Phthalic acid, 2-ethylhexyl 2-nitrophenyl ester [c]						32.635	2.08						
Phthalic acid, 3,5-dimethylphenyl 2-ethylhexyl ester [c]						32.599	0.80						
Phthalic acid, 4,4-dimethylpent-2-yl butyl ester						18.578	0.49			18.566	1.53		
										32.703	7.02		
Phthalic acid, butyl 4-nitrophenyl ester [f]												18.216	1.08
Phthalic acid, di-(-)-menthyl ester [a]		32.511	0.93										
Phthalic acid, di(2-ethylcyclohexyl) ester [e]										32.565	4.79		
Phthalic acid, di(6-methylhept-2-yl) ester [b]				32.530	5.98								
Phthalic acid, dodecyl octyl ester [b]				32.475	11.14								
Phthalic acid, hept-4-yl isobutyl ester [d]								17.344	0.22				
Terephthalic acid, dodecyl 2-ethylhexyl ester [a]		32.430	0.20										
Piperazine, 1-hex-2-ynyl-4-methyl- [e]	Piperazine derivative (n= 1)									26.180	0.73		
5,5,10,10-Tetrachlorotricyclo[7.1.0.0(4,6)]decane [d]	Polycyclic organic compound (n=1)							24.503	0.12				
5-Pregnen-3.beta.,9.alpha.-diol-20-one [e]	Pregnane steroid (n=2)									16.105	0.06		
Pregn-5-ene-3,8,11,12,14,20-hexol, (3.beta.,11.alpha.,12.beta.,14.beta.,20R)- [e]										16.094	0.06		
6H-Purine-6-thione, 9-amino-1,9-dihydro [c]	Purinethione (n=1)					32.560	0.45						
2-(2-Hydroxy-2-methyl-3-oxobutyl)-3,5,6-trimethylpyrazine [c]	Pyrazine (n=1)					13.529	0.72						
3,6-Epoxy-2H,8H-pyrimido[6,1-b][1,3]oxazocine-8,10(9H)-dione, 3,4,5,6-tetrahydro-4,5-dihydroxy-, [3R-(3.alpha.,4.alpha.,5.alpha.,6.alpha.)] [b]	Pyrimidine derivative (n=1)			18.780	0.02								
(R)-(-)-4-Methylhexanoic acid [d]								7.524	0.50				
Eicosanoic acid [d]								20.068	0.61				
n-Hexadecanoic acid		18.729	0.51	18.727	0.98							18.394	2.73
Nonanoic acid [d]								14.286	0.16				
Octadecanoic acid [d]								24.269	0.70				
Pentadecanoic acid						18.775	1.09	16.148	0.42	16.372	0.50		
								16.279	1.78				
								18.467	2.56				
Tetradecanoic acid						15.557	0.47	15.350	0.35	15.552	0.21		
Tridecanoic acid								13.945	0.19				

**Longifolene-I2** [d]	Sesquiterpene (n=1)							15.501	**0.24**				
**3.beta.-Hydroxyguaia-4(15),10(14),11(13)-trien-6,12-olide 8-(.alpha.,.beta.-dihydroxybutyrate)** [a]	Sesquiterpenoid (n=1)	13.865	**0.03**										
**1-Oxa-7,8-diazaspiro[4.4]nona-2,6-diene-6-carboxylic acid, 9,9-dimethyl-4-oxo-2-phenyl-, methyl ester** [f]	Spiro compound (n=1)											19.223	**0.67**
**9,10-Secoergosta-7,10(19),22-triene-3,5,6-triol, (3.beta.)-** [c]	Steroid derivative (n=4)					15.310	**0.03**						
**9,19-Cyclolanostan-3-ol, 24,24-epoxymethano-, acetate** [c]						15.675	**0.04**						
**Androstan-11-one, (5.alpha.)-** [e]										32.930	**0.31**		
**Cholest-22-ene-21-ol, 3,5-dehydro-6-methoxy-, pivalate** [e]										32.666	**1.18**		
**6.beta.,6.beta.-Dibromo-6,7-methylenetestosterone** [d]	Steroid hormone (n=1)							17.403	**0.42**				
**Bicyclo[4.1.0]octane, 7,7-dichloro-1-methyl-** [f]												21.391	**0.64**
**Butane, 1-(2,2-dichloro-3,3-dimethylcyclopropyl)-** [d]	Substituted cycloalkane (n=2)							21.954<br>18.845<br>19.399<br>20.716<br>21.500<br>22.307	**0.07**<br>**0.29**<br>**0.23**<br>**0.22**<br>**0.23**<br>**0.10**				
**Propane, 1-(2,2-dichloro-1,3,3-trimethylcyclopropyl)-** [f]												21.210	**0.65**
**1-Octadecanesulphonyl chloride**	Sulphonic acids and derivatives (n= 1)	17.977	**0.35**	18.600	**0.02**					15.001<br>17.680<br>17.964<br>23.825	**0.27**<br>**0.27**<br>**0.52**<br>**0.28**		
**6-Tetradecanesulfonic acid, butyl ester** [c]	Sulphonic ester (n=1)					14.660	**0.23**						
**Methyl tetrahydroionol** [e]	Terpenoid alcohol (n= 1)									25.930	**0.10**		
**2-Octanethiol, thiolacetate** [c]	Thioester (n=1)					13.942	**0.42**						
**1-[3-(1-Adamantyl)-1-methylpropylidene]thiosemicarbazide** [d]	Thiosemicarbazide (n=1)							14.984	**0.43**				
**N1-(tert-Butyl)-2-(3-pyridylmethylidene)hydrazine-1-carbothioamide** [a]	Thiosemicarbazone (n=1)	16.085	**0.04**										
**13-Tetradece-11-yn-1-ol** [d]	Unsaturated alcohol (n=1)							14.476	**0.41**				
**2-Dodecenoic acid** [a]	Unsaturated fatty acid (n=4)	15.095	**0.04**										
**5,8,11,14-Eicosatetraynoic acid** [d]								17.287	**0.35**				
**6-Octadecenoic acid, (Z)-**		23.839	**0.45**					23.545	**0.20**				
**cis-10-Heptadecenoic acid**								23.453	**3.07**			23.272	**4.87**

**Percentage of compounds exclusive to specific isolates**	**61.25%** (49/80)	**56.52%** (26/46)	**70.59%** (48/68)	**76.14%** (67/88)	**61.54%** (48/78)	**55.00%** (11/19)

[a] compounds exclusive to AB2-SP, [b] compounds exclusive to SP1-AB4, [c] compounds exclusive to SP2-AB7, [d] compounds exclusive to SP2-W6, [e] compounds exclusive to SP5-AB7, [f] compounds exclusive to Medium control.

* excludes compounds exclusively produced by the Mannitol medium control.

RT- retention time

Major compounds of AB2-SP, SP1-AB4, SP2-AB7, SP2-W6 and SP5-AB7 highlighted in blue, yellow, green, pink and grey, respectively (%Area ≥1.5).

Table S4. Gas chromatography-mass spectrometry (GC-MS) analysis of 5 sponge-associated *Bacillus* species medium 5294 extracts and the medium 5294 fermentation control.

Compounds (n=195)*	Classification	medium 5294 extracts											
		AB2-SP (n=97)		SP1-AB4 (n=94)		SP2-AB7 (n=11)		SP2-W6 (n=11)		SP5-AB7 (n=43)		medium 5294 Control (n= 14)	
		RT (min)	%Area	RT (min)	%Area	RT (min)	%Area	RT (min)	%Area	RT (min)	%Area	RT (min)	%Area
**1-Decanol, 5,9-dimethyl-** [b]				17.120	**0.66**								
**1,7-Octanediol, 3,7-dimethyl-** [e]										12.180	**0.28**		
**1-Pentanol, 5-(methylenecyclopropyl)-** [d]								12.346	**1.20**				
**2-Methyl-tridecane-2,12-diol**		15.765	**0.70**	15.780	**0.94**								
**3,3-Diethoxy-1-propanol** [a]		10.910	**0.14**										
**3-Hexanol, 2,4-dimethyl-** [c]						10.175	**5.28**						
**3-Octen-2-ol, (Z)-** [e]										14.906	**0.27**		
**5,7-Dodecadiyn-1,12-diol**		14.999	**0.20**	17.836	**0.48**								
		17.846	**0.44**	17.995	**1.55**								
		22.501	**0.40**	21.116	**0.91**								
	Alcohol (n=15)			14.418	**0.26**								
				18.155	**1.46**								
**7-Heptadecanol, 7-methyl-** [b]				14.748	**0.54**								
				19.769	**0.28**								
**Cyclohexane-1,2-diol, 4-(bicyclo[2.2.1]hept-2-yl)-** [a]		14.663	**0.63**										
**Exo-2,7,7-trimethylbicyclo[2.2.1]heptan-2-ol** [b]				15.355	**0.27**								
**n-Nonadecanol-1** [a]		15.511	**0.26**										
**n-Tridecan-1-ol** [a]		11.332	**0.03**										
**Phenol, 4-(1,1-dimethylpropyl)-** [a]		11.661	**0.09**										
**Z,Z-8,10-Hexadecadien-1-ol**		23.285	**0.20**	17.594	**0.75**								
**1,1-Diformyl-8,8-dichloroocta-1,3,5,7-tetraene** [b]				21.635	**0.75**								
**2-Decenal, (E)-** [b]				9.925	**0.08**								
**2-Tridecenal, (E)-** [a]	Aldehyde (n=6)	9.927	**0.06**										
**Butanal** [e]										8.354	**0.44**		
**Cinnamaldehyde, alpha-pentyl-**		14.113	**0.37**	14.108	**0.20**								
**E-15-Heptadecenal** [f]												13.627	**0.74**
**Butyl aldoxime, 2-methyl-, syn-** [e]	Aldoxime (n=1)									6.930	**0.86**		
**2-Methylhexacosane**		15.665	**0.20**	15.647	**0.26**								
**Decane, 2,3,5,8-tetramethyl** [a]		14.522	**0.71**										
**Dodecane, 2,6,11-trimethyl-** [a]		17.003	**0.68**										
**Eicosane**	Alkane (n=8)	25.181	**0.18**	25.175	**0.22**								
**Heptadecane** [b]				15.579	**0.25**								
**Heptadecane, 2,6,10,15-tetramethyl-**		15.590	**0.24**	14.491	**0.31**								

Hexadecane, 5-butyl- [f]												17.329	0.46
Oxetane, 2-(1,1-dimethylethyl)-3-methyl- [e]										18.345	2.88		
1,3,7-Cyclodecatriene, 1,7-dimethyl-, (Z,E,E,)- [e]	Alkene (n=3)									17.355	0.22		
1-Decene, 2,4-dimethyl- [e]										21.505	0.22		
Z,Z,Z-1,4,6,9-Nonadecatetraene [a]		17.602	0.54										
Ethanol, 2-(pentyloxy)-, acetate [a]	Alkoxy acetate (n=2)	13.214	1.20										
Isopentyloxyethyl acetate		13.982	0.44	13.008	0.18								
Trans-3-Undecene-1,5-diyne	Alkyne (n=1)	19.530	0.17	24.500	0.10								
Acetamide, N-(2-phenylethyl)- [a]		12.894	0.11										
Acetamide, N,N'-carbonylbis [a]		11.841	0.07										
Butanamide [e]	Amide (n=4)									8.790	1.30		
										8.865	0.92		
Propanamide, 2-methyl- [e]										6.219	3.65		
										5.620	0.52		
Arginine, N(2)-p-toluenesulfonyl-, t-butyl ester [e]	Amino acid derivative (n=1)									20.590	0.60		
7,9-Di-tert-butyl-1-oxaspiro(4,5)deca-6,9-diene-2,8-dione	Antioxidant (n=1)	17.306	0.58	17.313	0.74								
Benzeneacetic acid	Aromatic carboxylic acid (n=1)			10.143	1.24	10.285	3.29	10.249	3.96	10.436	2.79		
Isoamyl salicylate [a]	Aromatic ester (n=1)	12.986	0.05										
Cyclooctylidene-(2-phenylaziridin-1-yl)amine	Aziridine (n=1)	16.223	0.36	16.216	0.73								
Benzofuran, 7-(2,4-dinitrophenoxy)-2,3-dihydro-2,2-dimethyl- [b]	Benzofuran (n=1)			19.030	0.35								
Benzoic acid [b]	Benzoic acid derivatives (n=1)			9.175	0.09								
5-Chloro-2-nitrobenzyl alcohol [a]	Benzyl alcohol (n=1)	24.545	0.06										
Benzonitrile, 3-benzyloxy- [a]	Benzyl ether (n=1)	18.948	3.20										
9,9-Dimethoxybicyclo[3.3.1]nona-2,4-dione [b]	Bicyclic ketone (n=2)			15.489	0.99								
Bicyclo[4.2.0]octan-7-one, 8,8-dichloro-1-methyl-		15.086	0.28	14.861	0.32								
		15.368	0.19	15.082	0.29								
		16.140	0.64										
		14.870	0.21										
Tricyclo[3.1.0.0(2,4)]hexane, 3,3,6,6-tetracyclopropyl- [a]	Bridged bicyclic compound (n=1)	19.363	0.33										
4-Dihexylcarbamoyl-butyric acid [b]	Carbamic acid (n=1)			11.733	0.04								
Bicyclo[10.1.0]trideca-4,8-diene-13-carboxamide, N-(3-chlorophenyl)- [e]	Carboxamide (n=1)									18.555	1.16		
										18.851	0.28		
2-(1-Cyclohexyl-1H-tetrazol-5-ylsulfanyl)-propionic acid [b]	Carboxylic acid (n=1)			22.155	0.48								
1,3-Dioxolane-4-methanol, 2-ethyl- [a]	Cyclic acetal(dioxolane) (n=1)	10.418	0.17										

**1,3-Dioxolane-4-methanol, 2,2-dimethyl-, acetate [a]**	Cyclic ether acetate (n=1)	10.520	**0.03**										
**Cyclohexanemethanol, .alpha,alpha.,4-trimethyl- [b]**	Cycloalcohol (n=1)			14.663	**0.54**								
**1,3-Cyclopentadiene, 1,3-bis(1-methylethyl)- [b]**	Cycloalkene (n=1)			12.317	**0.09**								
**2,4-Bis(diazo)adamantane**	Diazo compound (n=1)	20.589	**0.47**	19.855	**0.42**								
**3-Octene-2,6-dione, 5,5,7-trimethyl-, (E)- [a]**	Diketone (n=1)	11.910	**0.04**										
**2,3-Dioxabicyclo[2.2.1]heptane [e]**	Dioxabicycloalkane (n=1)									14.280	**0.22**		
**(-)Isolongifolol, acetate [a]**		8.963	**0.59**										
**Adipic acid, isobutyl 2-methoxyethyl ester**		19.602	**0.32**	19.589	**0.45**								
**Ethyl 9-decenoate [e]**										6.335	**0.05**		
**l-(+)-Ascorbic acid 2,6-dihexadecanoate [f]**												18.665	**0.99**
**(S)-(-)-1,2,4-Butanetriol, 4-acetate**		9.057	**1.78**	9.065	**0.10**			9.105	**0.77**	9.471	**0.49**		
**1,2,3-Propanetriol, 1-acetate [a]**		9.755	**0.14**										
**2,2,4-Trimethyl-1,3-pentanediol diisobutyrate [b]**				10.344<br>11.610<br>12.140	**0.09**<br>**0.04**<br>**0.09**								
**2-Methyl-5-oxohexanethioic acid, S-t-butyl ester [a]**		11.150	**0.12**										
**4-Pentenoic acid, 2-acetyl-, ethyl ester [e]**										11.155	**0.08**		
**9,12-Octadecadienoic acid, methyl ester [a]**		22.914	**0.86**										
**Acetic acid, 4a-methyldecahydronaphthalen-1-yl ester [a]**	Ester (n=47)	12.318	**0.12**										
**Acetic acid, butoxyhydroxy-, butyl ester**						12.306	**1.10**	12.306	**1.03**				
**Adipic acid, butyl 2-ethylhexyl ester [a]**		19.779	**0.34**										
**Benzoic acid, 2-hydroxy-, phenylmethyl ester**		16.925	**0.50**	16.929<br>16.929	**0.57**<br>**0.57**								
**Butanedioic acid, bis(2-methylpropyl) ester [e]**										13.296	**1.62**		
**Butanoic acid, 2-[(phenylmethoxy)imino]-, trimethylsilyl ester [e]**										20.650	**0.42**		
**Butanoic acid, 4-(1,1-dimethylethoxy)-3-hydroxy-, methyl ester, (R)- [a]**		10.138	**0.38**										
**Butyl isocyanatoacetate [e]**										12.248	**0.34**		
**Butyric acid, 2,2-dimethyl-, vinyl ester [a]**		10.346<br>11.611	**0.05**<br>**0.07**										
**Butyric acid, 3-tetradecyl ester [b]**				11.465	**0.11**								

**Carbonic acid, allyl pentadecyl ester [a]**		17.123	**0.54**										
**Cyclohexanecarboxylic acid, 2-hydroxy-, ethyl ester**		14.270	**0.13**	14.265	**0.09**								
**Decanoic acid, hexyl ester [d]**								16.226	**0.66**				
**Dibutyl adipate**		14.754	**0.59**			14.423	**15.12**	14.430	**18.14**				
**Dodecanoic acid, isooctyl ester**		21.495	**1.55**	21.490	**1.78**							22.059	**2.85**
**Glutaric acid, 2-methoxyethyl pentadecyl ester [b]**				13.705	**0.43**								
**Glutaric acid, butyl 5-methylhex-2-yl ester [a]**		17.424	**0.23**										
**Glutaric acid, di(isobutyl) ester**		13.305	**3.27**	13.304	**3.31**							13.472	**2.40**
**Glycerol 1,2-diacetate $$ 1,2,3-Propanetriol, 1,2-diacetate [a]**		10.753	**0.03**										
**Hexadecanoic acid, methyl ester [b]**				17.418	**0.31**								
**Hexanedioic acid, bis(2-methylpropyl) ester**		14.363	**7.06**	12.798	**0.11**					14.352	**5.60**	14.529	**11.29**
				14.363	**7.43**								
**Isopropyl myristate**		15.894	**0.36**	15.883	**0.37**							16.164	**0.53**
**Isopropyl palmitate [a]**		19.400	**0.37**										
**Methyl 3,5-tetradecadiynoate [a]**		20.354	**0.26**										
**Octadecanoic acid, 9,10-epoxy-18-(trimethylsiloxy)-,methyl ester, cis [b]**				18.510	**2.22**								
**Octanoic acid, 2-ethylhexyl ester [b]**				14.953	**0.97**								
**Oxalic acid, 2-ethylhexyl octadecyl ester [b]**				30.310	**0.25**								
**Pentanedioic acid, bis(1-methylpropyl) ester**						13.359	**4.55**	13.361	**5.64**				
**Pentanoic acid, 2,2,4-trimethyl-3-carboxyisopropyl, isobutyl ester**		13.440	**0.44**	13.437	**0.44**					10.335	**0.57**		
**Pentanoic acid, 2,2,4-trimethyl-3-hydroxy-, isobutyl ester**						11.021	**3.53**	11.020	**2.09**				
						11.281	**4.14**						
**Pentanoic acid, 2,4-dimethyl-3-oxo-, methyl ester [d]**								11.280	**2.80**				
**Propanoic acid, 2-methyl-, 2,2-dimethyl-1-(2-hydroxy-1-methylethyl)propyl ester**		10.977	**1.88**	10.977	**1.51**					10.991	**3.24**		
		12.142	**0.15**										
**Propanoic acid, 2-methyl-, 3-hydroxy-2,4,4-trimethylpentyl ester**		11.235	**2.00**	11.234	**1.55**					11.245	**3.13**		
**Propanoic acid, 2-methyl-, nonyl ester [a]**		11.475	**0.60**										
**Succinic acid, 2-heptyl isobutyl ester [b]**				12.256	**0.57**								
**Succinic acid, 3,3-dimethylbut-2-yl isobutyl ester [a]**		12.257	**0.68**										
**Propane, 1,1'-[ethylidenebis(oxy)]bis- [b]**	Ether (n=1)			9.970	**0.22**								
**Eicosyl acetate [b]**	Fatty acid ester (n=1)			25.607	**0.28**								
**Decanamide, n-pentyl- [a]**	Fatty acid amide (n=1)	24.595	**0.12**										

1-Tetradecyl acetate [b]	Fatty acid ester (n=1)			11.330	0.15								
3,4-Dihydroxy-5-methyl-dihydrofuran-2-one [e]	Furanone (n=1)									6.815	0.93		
2-Bromotetradecane [b]				17.001	0.70								
Butane, 1,1,2-tribromo- [e]										27.110	0.16		
Butane, 2-iodo-3-methyl-				16.304	0.91								
Cyclopropane, (3-chloropropyl)methylene-		22.648	0.23	16.130	0.72							18.760	0.62
Cyclopropane, 1,1-dichloro-2,2,3-triethyl-	Haloalkane (n=7)	15.041	0.19	15.030	0.18								
				22.479	0.41								
				23.580	0.08								
Cyclopropane, 1,1-dichloro-2,2-diethyl-3,3-dimethyl-		21.110	0.77	19.358	0.58								
				19.495	0.29								
Propane, 1-(2,2-dichloro-1,3,3-trimethylcyclopropyl)- [b]				19.087	1.26								
				20.941	0.61								
(2E,4E)-5-Chloro-3,4-dimethyl-2,4-heptadiene				18.644	0.67					18.185	1.40		
(4Z)-5-Chloro-3,4-dimethyl-2,4-heptadiene [e]										18.035	0.50		
1-Heptene, 5,7,7,7-tetrachloro- [a]	Haloalkene (n=6)	18.734	0.46										
2,11-Dodecadiene, 4-chloro- [b]				15.164	0.12								
7-Heptadecene, 1-chloro- [a]		20.250	0.36										
Adamantane-1-(3,3-dichloropropyn-1-yl) [a]		18.458	0.53										
1-Chloro-4-decyne [a]	Haloalkyne (n=1)	16.045	0.44										
2H-Pyran, 2-(bromomethyl)tetrahydro- [a]	Heterocyclic compound (n=1)	8.890	0.09										
Pyrrolo[1,2-a]pyrazine-1,4-dione, hexahydro-3-(2-methylpropyl)-	Heterocyclic compound (diketopiperazine) (n=1)	16.300	1.13							18.688	1.40		
		17.727	0.85										
		18.066	2.20										
		14.422	0.56										
2,4-Imidazolidinedione, 5-(2-methylpropyl)-, (S)-	Hydantoin (n=3)	13.753	3.82	13.660	0.98							13.757	1.75
2,4-Imidazolidinedione, 5-methyl-		11.528	0.59	11.405	0.18								
5-Isopropyl-2,4-imidazolidinedione		12.705	0.89	12.644	0.27								
Cyclopropane-1-carbohydrazide, 2-phenyl-N2-(3-methylcyclohexylideno)- [b]	Hydrazide (n=1)			17.779	0.65								
N-Benzylsuccinamic acid N'-[(2-hydroxy-1-naphthyl)methylene]hydrazide [e]	Hydrazine (n=1)									20.490	0.30		
Ethanone, 1-(2-chlorophenyl)-, 2,4-dinitrophenylhydrazone [b]	Hydrazone (n=1)			22.700	0.14								
5-Amino-3,4-dimethyl-isoxazole [e]	Isoxazole derivative (n=1)									14.660	0.97		
2,5-Dimethyl-4-hydroxy-3-hexanone [b]	Ketol (n=1)			15.965	0.54								
3-Isopropyl-5-methylhexan-2-one [e]	Ketone (n=7)									14.500	1.45		

**Heptadecane-2,4-dione** [e]										14.595	**0.97**		
**1-Penten-3-one, 1-(2,6,6-trimethyl-2-cyclohexen-1-yl)-, (E)-** [b]				12.746	**0.08**								
**3-Nonen-2-one, 3-ethyl-** [b]				13.145	**0.06**								
**3-Pentadecanone** [a]		15.960	**0.54**										
**9-Oxa-bicyclo[3.3.1]nona-3,6-dien-2-one** [e]										12.565	**0.96**		
**Cycloheptanone, 2-(3-buten-1-yl)-** [b]				30.260	**0.12**								
**Propanedinitrile, bicyclo[3.3.1]non-9-ylidene-**	Nitrile (n=2)	15.225	**0.16**	15.217	**0.15**								
		20.846	**0.42**	16.034	**0.27**								
**Propionitrile, 3-[1-[4-[1-(2-cyanoethoxy)cyclohexyl]buta-1,3-diynyl]cyclohexyloxy]-**		16.810	**0.83**	16.814	**1.04**								
		19.875	**0.23**	20.219	**0.40**								
		20.755	**0.24**	22.640	**0.16**								
		20.930	**0.47**										
		18.160	**0.86**										
**2-Nitrohept-2-en-1-ol** [a]	Nitroalkene (n=1)	14.926	**0.62**										
**2-(3,3-Dimethyl-but-1-ynyl)-1,1,3-trimethyl-cyclopropane** [c]	Organic compound (n=1)					16.550	**0.32**						
**Germacycloundecane, 1,1-diethyl-** [b]	Organogermanium compound (n=1)			26.009	**0.04**								
**2-(Dimethyl(chloromethyl)silyloxy)tridecane** [b]	Organosilicon (n=3)			20.725	**0.13**								
**bis[(4Z)-Dec-4-en-1-yloxy](dimethyl)silane** [b]				22.139	**0.40**								
**1-Methyl-1-(10-undecenyl)oxy-1-silacyclobutane** [a]		21.895	**0.23**										
**1,2,5-Oxadiazol-3-amine, 4-(phenylmethoxy)-** [b]	Oxadiazole (n=2)			18.845	**2.08**								
**1,3,4-Oxadiazole-2(3H)-thione, 3-(4-morpholylmethyl)-5-phenoxymethyl-** [f]												13,83	**1.17**
**Oxazolidin-2-one, N-[(E)-butenoyl]-** [a]	Oxazolidinone (n=1)	12.571	**0.19**										
**1,2-Benzenedicarboxylic acid, bis(2-methylpropyl) ester**	Phthalate ester (n=5)	16.611	**21.46**	16.633	**21.92**	16.712	**54.02**	16.745	**61.93**	16.614	**17.37**	16.838	**66.45**
		17.252	**0.29**	17.245	**0.38**								
**Bis(tridecyl) phthalate**		13.517	**0.22**	13.512	**0.14**								
**Diisooctyl phthalate**				29.931	**1.29**							31.250	**7.55**
**Phthalic acid, di(2-propylpentyl) ester** [a]		30.904	**1.97**										
		30.942	**1.36**										
**Phthalic acid, di(6-methylhept-2-yl) ester** [b]				29.885	**1.73**								
**5,5,10,10-Tetrachlorotricyclo[7.1.0.0(4,6)]decane**	Polycyclic organic compound (n=1)	19.276	**0.46**	21.346	**0.60**								
		24.281	**0.09**	21.781	**0.93**								
		24.834	**0.17**	24.300	**0.16**								
**4-(2,3-Dimethoxybenzylidene)-3-methyl-1-(4-nitrophenyl)-2-pyrazolin-5-one** [b]	Pyrazolone (n=1)			20.350	**0.31**								

**5-Pyrimidinecarboxylic acid, hexahydro-5-(1-methylethyl)-2,4,6-trioxo- [e]**	Pyrimidine derivative (n=1)									14.725	**0.14**
**4-Methyloctanoic acid**		9.795	**0.15**							7.572	**0.53**
**Octadecanoic acid**		24.024	**0.55**	24.045	**0.82**						
**Octanoic acid [b]**				8.988	**0.10**						
**(R)-(-)-4-Methylhexanoic acid [a]**		7.482	**0.09**								
**7-Nonynoic acid [e]**										16.149	**0.24**
**n-Hexadecanoic acid [c]**						18.601	**5.95**				
**Pentadecanoic acid**	Saturated fatty acid (n=10)	13.912	**0.15**	12.925	**0.06**						
		18.332	**2.77**	13.977	**0.13**						
				16.076	**0.23**						
				18.347	**3.78**						
**Propionic acid, 3-tetrazol-1-yl- [e]**										7.430	**0.31**
**Tetradecanoic acid**		15.291	**0.27**	15.294	**0.43**						
**Tridecanoic acid [b]**				13.908	**0.25**						
**Alloaromadendrene [a]**	Sesquiterpene (n=1)	15.444	**0.26**								
**2-Ethyl(dimethyl)silyloxytetradecane**	Silyl ether (n=2)					24.175	**0.27**				
**Isotridecyl alcohol, trimethylsilyl derivative [a]**		24.965	**0.24**								
**Spiro[bicyclo[2.2.2]oct-5-ene-2,2'-(1',3'-dioxolane)], 4,4',5'-trimethyl- [e]**	Spiro compound (n=1)									15.870	**0.40**
**Spiro[androst-5-ene-17,1'-cyclobutan]-2'-one, 3-hydroxy-, (3.beta.,17.beta.)- [e]**	Spirocyclic steroid (n=1)									32.485	**39.29**
**1-Oxaspiro[2.5]octan-4-one, 2-(4-chlorophenyl)-5,5-dimethyl- [a]**	Spiroketone (n=1)	21.347	**0.42**								
**1,1'-Biphenyl, 2,2',5,5'-tetramethyl-**	Substituted biphenyl (n=1)	14.217	**0.18**	14.211	**0.10**						
**Butane, 1-(2,2-dichloro-3,3-dimethylcyclopropyl)-**	Substituted cycloalkane (n=3)	19.081	**1.07**	19.278	**0.53**						
		19.165	**0.34**	18.715	**0.42**						
		20.460	**0.48**	18.941	**0.60**						
		20.696	**0.50**	20.030	**0.28**						
		21.255	**0.98**	20.446	**0.52**						
		21.651	**0.38**	20.672	**0.55**						
		21.787	**0.57**	21.248	**1.18**						
		21.985	**0.48**	21.910	**0.33**						
		22.135	**0.73**	21.970	**0.46**						
		22.322	**0.43**	22.331	**0.56**						
		24.480	**0.06**	24.576	**0.11**						
				25.385	**0.09**						
				26.064	**0.06**						
				26.388	**0.03**						
				26.453	**0.08**						

**Butane, 1-(2,2-dichloro-3-ethylcyclopropyl)- [a]**		20.065	**0.24**										
**2,4-Monoethylidene-l-xylitol**	Sugar alcohol (n=1)					9.990	**2.43**						
**2H-Pyran, tetrahydro-2-methyl-**	Tetrahydropyran (n=2)							13.413	**1.20**				
**4-Chloro-3-n-butyltetrahydropyran [a]**		15.175	**0.13**										
**Thiazole, 5-ethyl-2-methyl- [e]**	Thiazole derivative (n=1)									14.775	**0.40**		
**1,3,5-Triazine-2,4-diamine, N(2)-ethyl-6-[[6-(phenylmethoxy)-3-pyridazinyl]oxy]- [e]**	Triazine derivative (n=1)									20.685	**0.12**		
**8,9-Dichlorotricyclo[4.3.1.0(2,5)]dec-7-ene [b]**	Tricyclic hydrocarbon (n=1)			24.530	**0.04**								
**5,8,11,14-Eicosatetraynoic acid**		17.201<br>17.481<br>18.530<br>18.642	**0.33**<br>**0.26**<br>**1.13**<br>**0.48**	17.190<br>17.484	**0.33**<br>**0.28**								
**6-Octadecenoic acid, (Z)- [f]**	Unsaturated fatty acid (n=6)											23.717	**2.51**
**9,12-Octadecadienoic acid (Z,Z)- [b]**				22.920	**0.99**								
**cis-10-Heptadecenoic acid [b]**				23.215	**2.59**								
**cis-Vaccenic acid [a]**		23.188	**1.79**										
**11-(2-Cyclopenten-1-yl)undecanoic acid, (+)- [d]**								16.340	**0.58**				
**Percentage of compounds exclusive to specific isolates**		**54.67%** (52/96)		**49.43%** (44/89)		**36.36%** (4/11)		**41.67%** (5/12)		**76.19%** (32/42)		**38.46%** (5/13)	

[a] compounds exclusive to AB2-SP, [b] compounds exclusive to SP1-AB4, [c] compounds exclusive to SP2-AB7, [d] compounds exclusive to SP2-W6, [e] compounds exclusive to SP5-AB7, [f] compounds exclusive to Medium control.

* excludes compounds exclusively produced by the Mannitol medium control.

RT- retention time

Major compounds of AB2-SP, SP1-AB4, SP2-AB7, SP2-W6 and SP5-AB7 highlighted in blue, yellow, green, pink and grey, respectively (%Area ≥1.5).

Qualitative agar-overlay assay for violacein inhibition

Table S5. Agar overlay assay results for 10 sponge-associated *Bacillus* species extracts, using bacterial biosensor *Chromobacterium violaceum* ATCC 12472.

Extract Code	Isolate source	Zone of violacein inhibition medium Mannitol (mm)*			Zone of violacein inhibition medium 5294 (mm)*		
		0.5 mg	1 mg	2 mg$	0.5 mg	1 mg	2 mg$
**SP-AB2**	*Bacillus thuringiensis*	0	8	14	0	0	0
**SP1-AB4**	*Bacillus cereus*	7	9	nt#	0	0	nt#
**SP2-AB7**	*Bacillus mobilis*	0	0	nt#	7	8	nt#
**SP2-W6**	*Bacillus pumilus*	7	9	nt#	0	8	nt#
**SP5-AB7**	*Bacillus wiedmannii*	0	8	nt#	0	0	nt#
**Furanone**!!	-	8	11				

*Values indicate the diameter of quenching, including the 6 mm wells.

$Concentration of 2 mg/mL only tested for those extracts which displayed putative activity at 1 mg/mL.

#nt – Not tested at this concentration.

!!Furanone, the positive quorum sensing inhibition control was tested at 0.005 and 0.01 mg, due to its bactericidal activity.

Pyocyanin, pyoverdine and growth inhibition following treatment with 10 sponge-associated *Bacillus* species extracts

A **B**

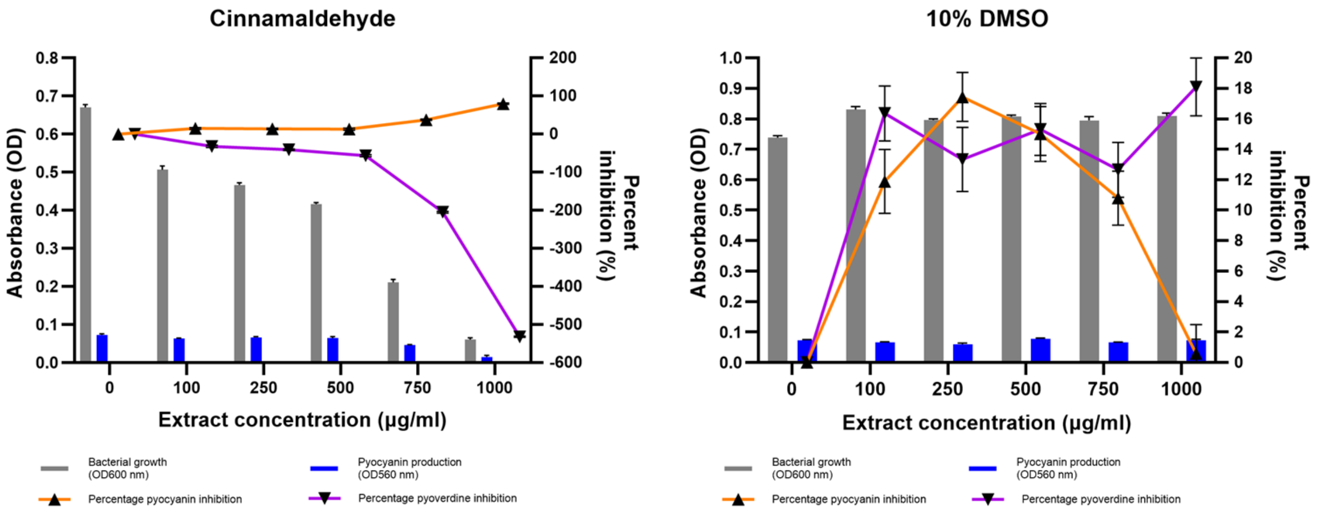

Figure S3.1. Inhibition of pyocyanin and pyoverdine using cinnamaldehyde and 10% DMSO**.** Positive control cinnamaldehyde (**A**) resulted in ≥40% *Pseudomonas aeruginos*a growth inhibition from 750 µg/mL, contributing to the decrease in pyocyanin production and is not indicative of QSI. A steep increase in pyoverdine was also observed. Solvent 10% DMSO (**B**) had a negligible effect on cell growth, as well as pyocyanin and pyoverdine production ($p > 0.05$). From 500 µg/ml, a reduction in percent pyocyanin inhibition was observed, which was unclear since minimal to no effect was observed for bacterial growth or pigment production. Data reflects average results ±SD following experiments in triplicate on three separate occasions.

A **B**

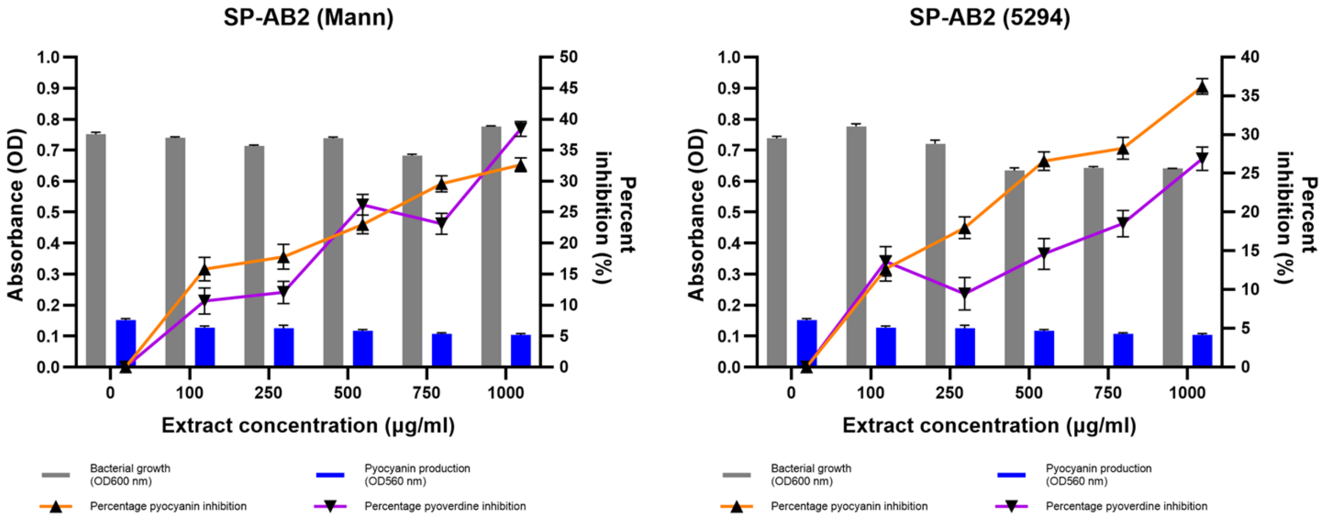

Figure S3.2. Inhibition of pyocyanin and pyoverdine using sponge-associated bacterial extracts**.** Effect of *Bacillus thuringiensis* SP-AB2 extracts on *Pseudomonas aeruginosa* growth, pyocyanin and pyoverdine production. (**A**) SP-AB2 (Mann) and (**B**) SP-AB2 (5294). Data reflects average results ±SD following experiments in triplicate on three separate occasions. Growth inhibition for both extracts was ≤40%.

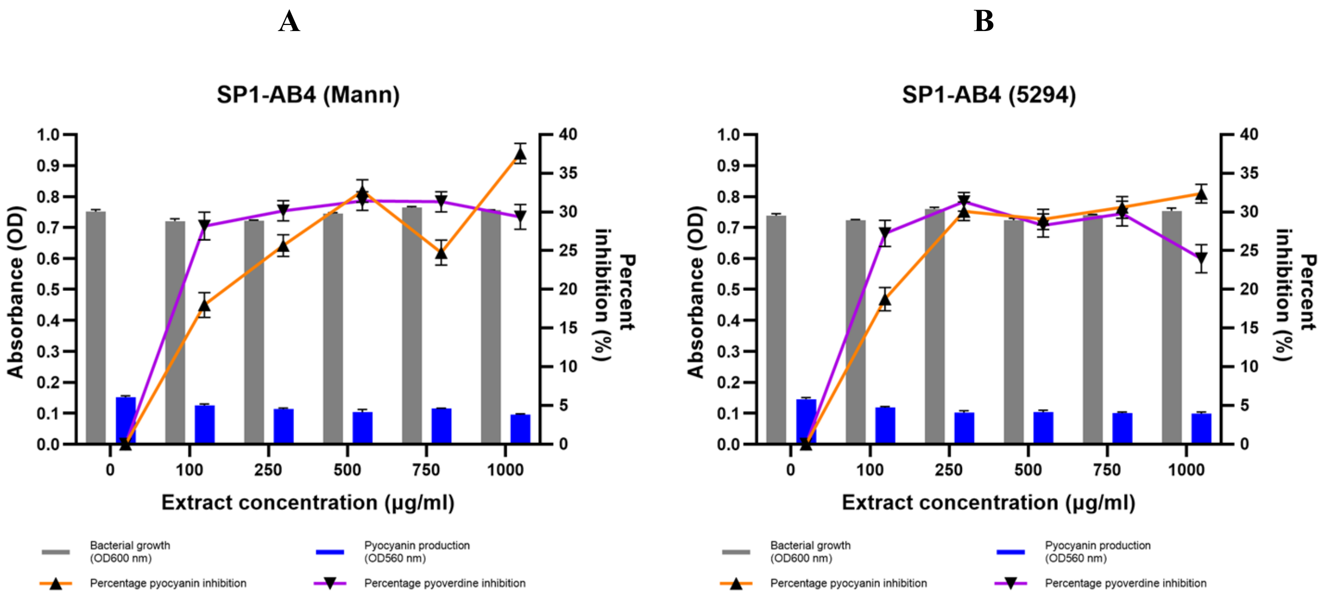

Figure S3.3. Inhibition of pyocyanin and pyoverdine using sponge-associated bacterial extracts**.** Effect of *Bacillus cereus* SP1-AB4 extracts on *Pseudomonas aeruginosa* growth, pyocyanin and pyoverdine production. (**A**) SP1-AB4 (Mann) and (**B**) SP1-AB4 (5294). Data reflects average results ±SD following experiments in triplicate on three separate occasions. Growth inhibition for both extracts was ≤40%.

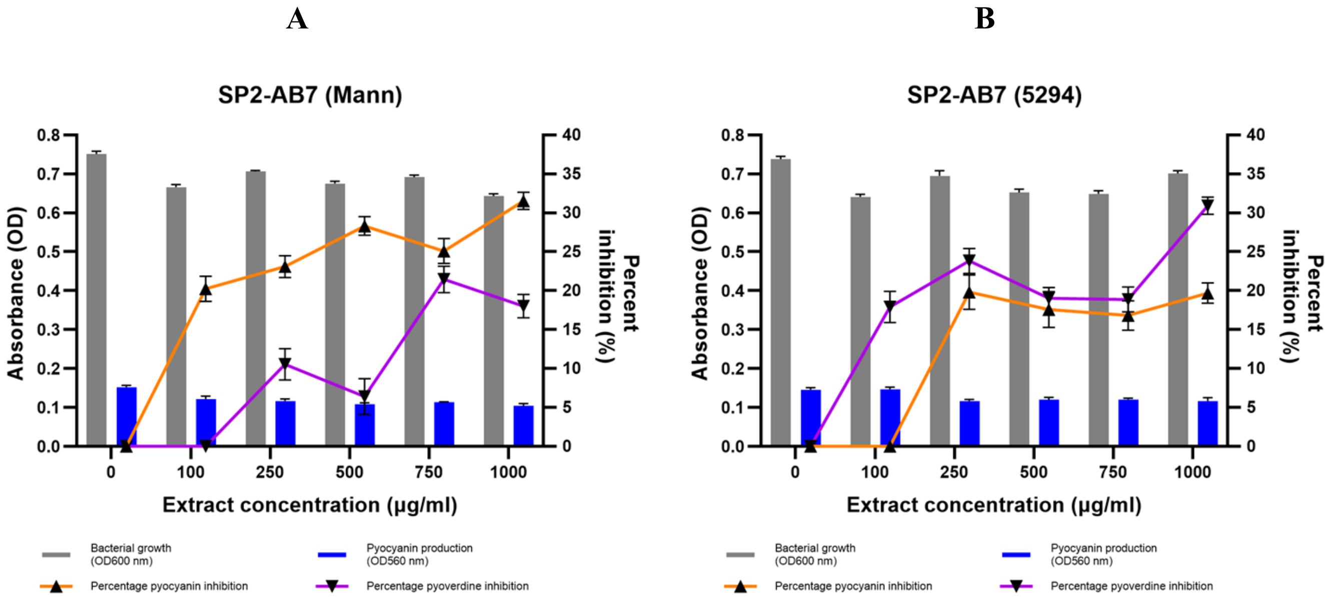

Figure 3.4. Inhibition of pyocyanin and pyoverdine using sponge-associated bacterial extracts**.** Effect of *Bacillus mobilis* SP2-AB7 extracts on *Pseudomonas aeruginosa* growth, pyocyanin and pyoverdine production. (**A**) SP2-AB7 (Mann) and (**B**) SP2-AB7 (5294). Data reflects average results ±SD following experiments in triplicate on three separate occasions. Growth inhibition for both extracts was ≤40%.

A B

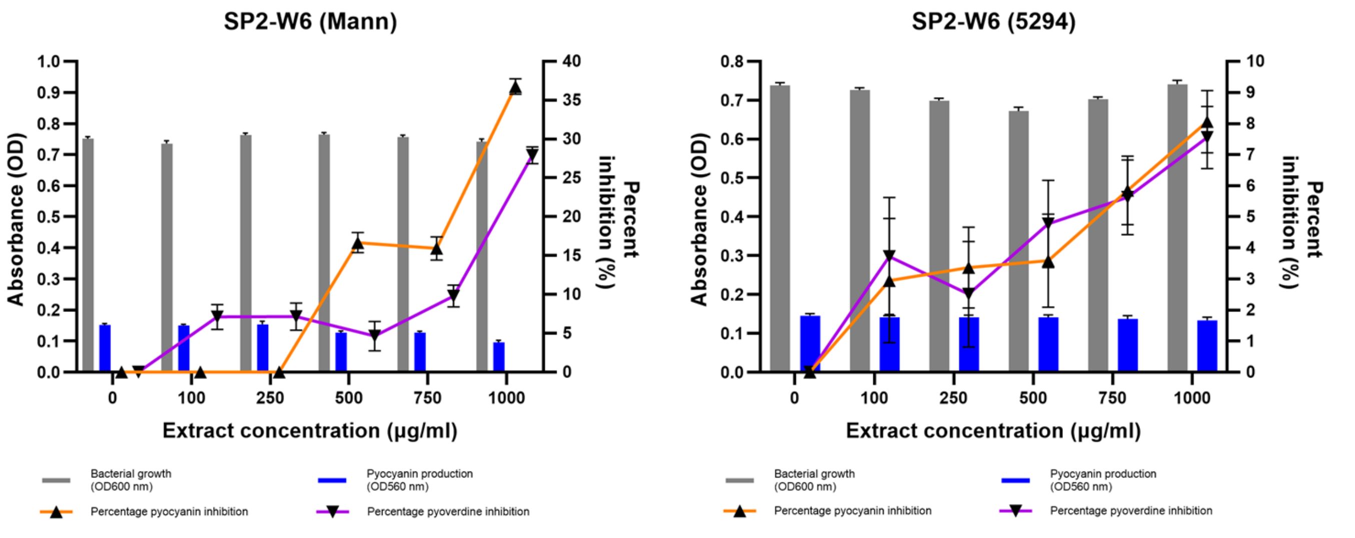

Figure S3.5. Inhibition of pyocyanin and pyoverdine using sponge-associated bacterial extracts**.** Effect of *Bacillus pumilus* SP2-W6 extracts on *Pseudomonas aeruginosa* growth, pyocyanin and pyoverdine production. (**A**) SP2-W6 (Mann) and (**B**) SP2-W6 (5294). Data reflects average results ±SD following experiments in triplicate on three separate occasions. Growth inhibition for both extracts was ≤40%.

A B

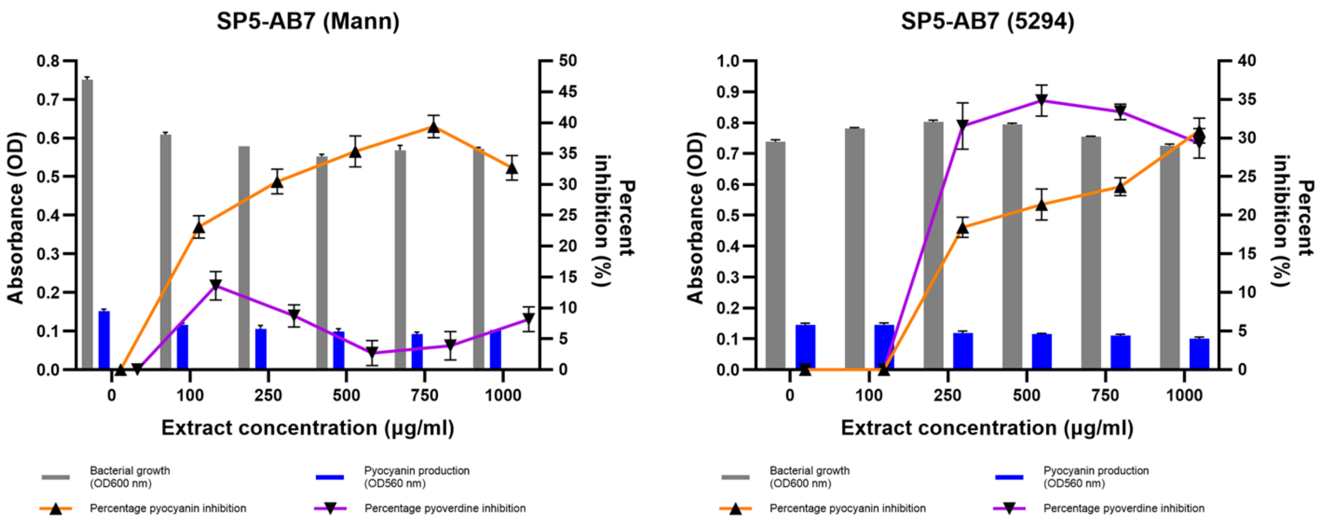

Figure S3.6. Inhibition of pyocyanin and pyoverdine using sponge-associated bacterial extracts**.** Effect of *Bacillus wiedmannii* SP5-AB7 extracts on *Pseudomonas aeruginosa* growth, pyocyanin and pyoverdine production. (**A**) SP5-AB7 (Mann) and (**B**) SP5-AB7 (5294). Data reflects average results ±SD following experiments in triplicate on three separate occasions. Growth inhibition for both extracts was ≤40%.

Elastase and growth inhibition following treatment with sponge-associated *Bacillus* species extracts

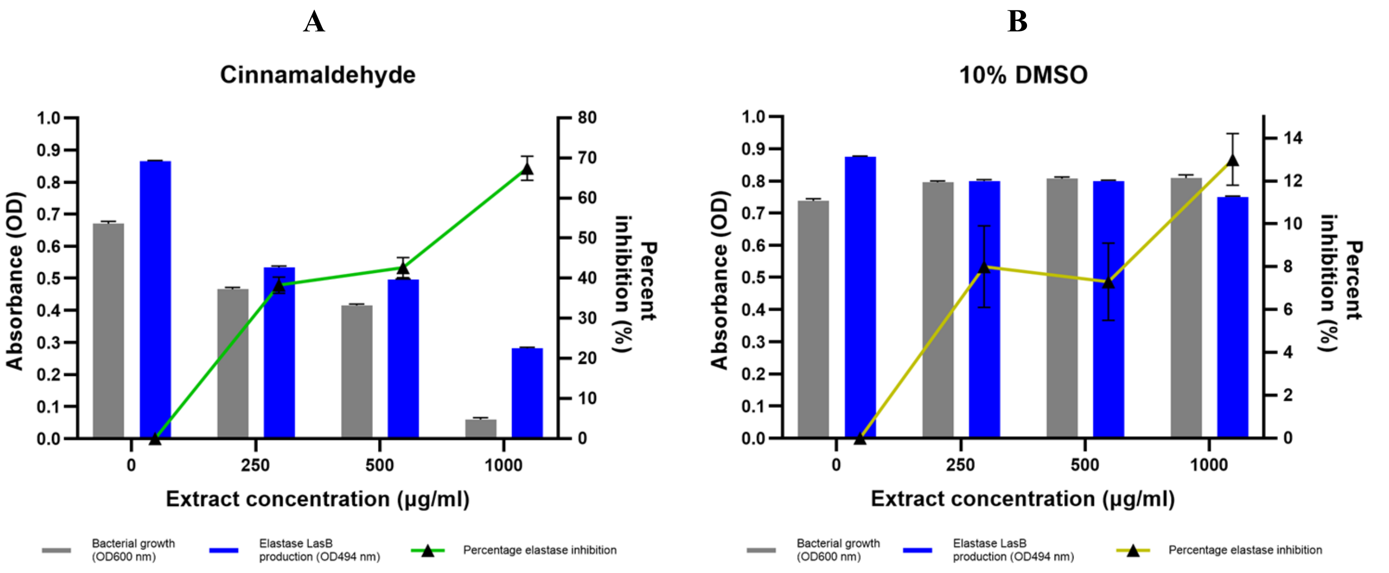

Figure S4.1. Inhibition of elastase LasB using cinnamaldehyde and 10% DMSO**.** Positive control cinnamaldehyde (**A**) resulted in cytotoxic effects from 750 µg/mL, to which the decrease in elastase may be attributed. Solvent 10% DMSO (**B**) resulted in an increase in cell growth but had a negligible effect on elastase production (p > 0.05). Data reflects average results ±SD following experiments in triplicate on three separate occasions.

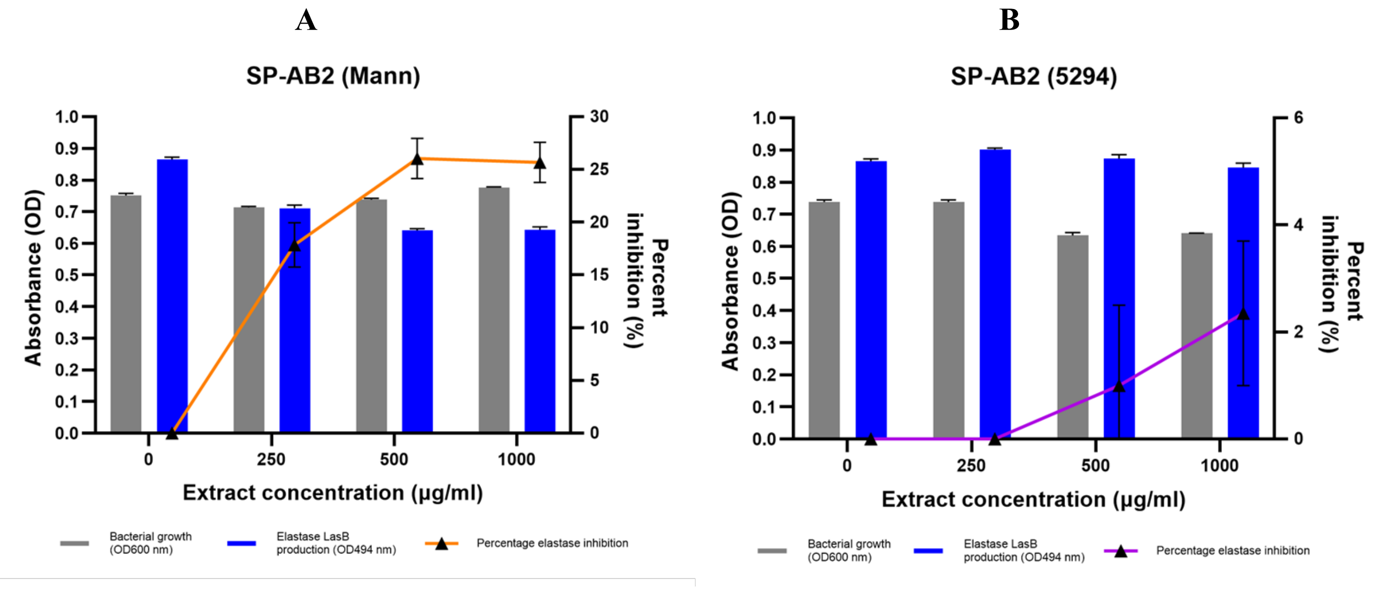

Figure S4.2. Inhibition of elastase LasB using sponge-associated bacterial extracts**.** Effect of *Bacillus thuringiensis* SP-AB2 extracts on *Pseudomonas aeruginosa* growth and elastase production. (**A**) SP-AB2 (Mann) and (**B**) SP-AB2 (5294). Data reflects average results ±SD following experiments in triplicate on three separate occasions. Growth inhibition for both extracts was ≤40%.

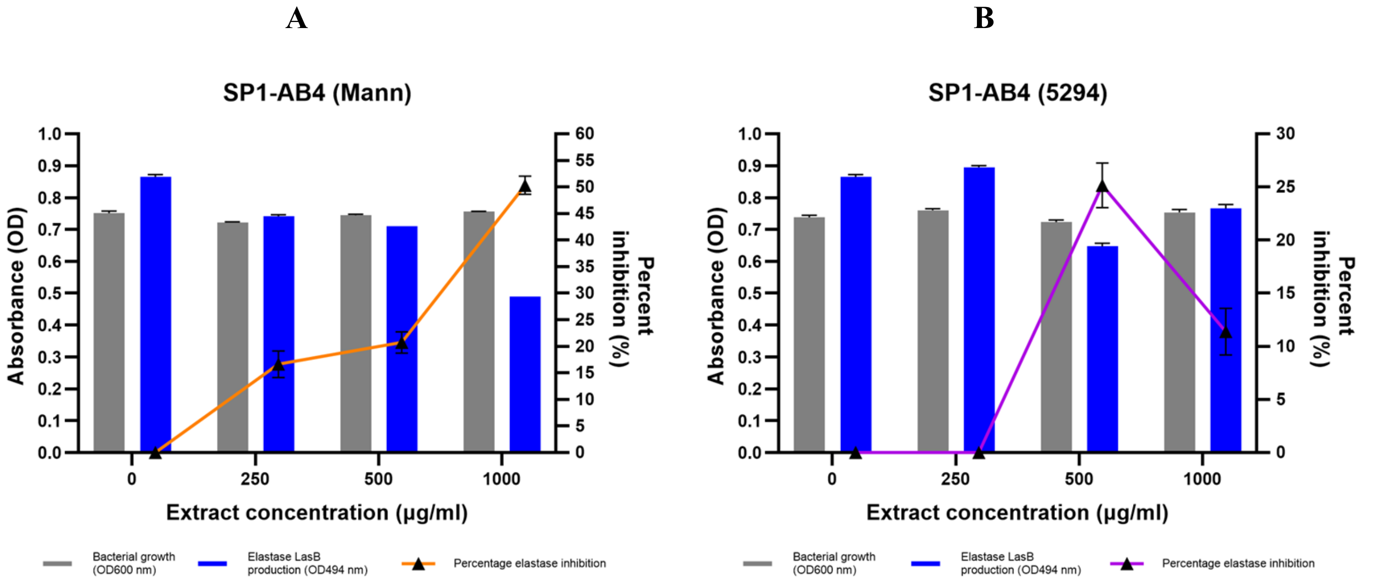

Figure S4.3. Inhibition of elastase LasB using sponge-associated bacterial extracts**.** Effect of *Bacillus cereus* SP1-AB4 extracts on *Pseudomonas aeruginosa* growth and elastase production. (**A**) SP1-AB4 (Mann) and (**B**) SP1-AB4 (5294). Data reflects average results ±SD following experiments in triplicate on three separate occasions. Growth inhibition for both extracts was ≤40%.

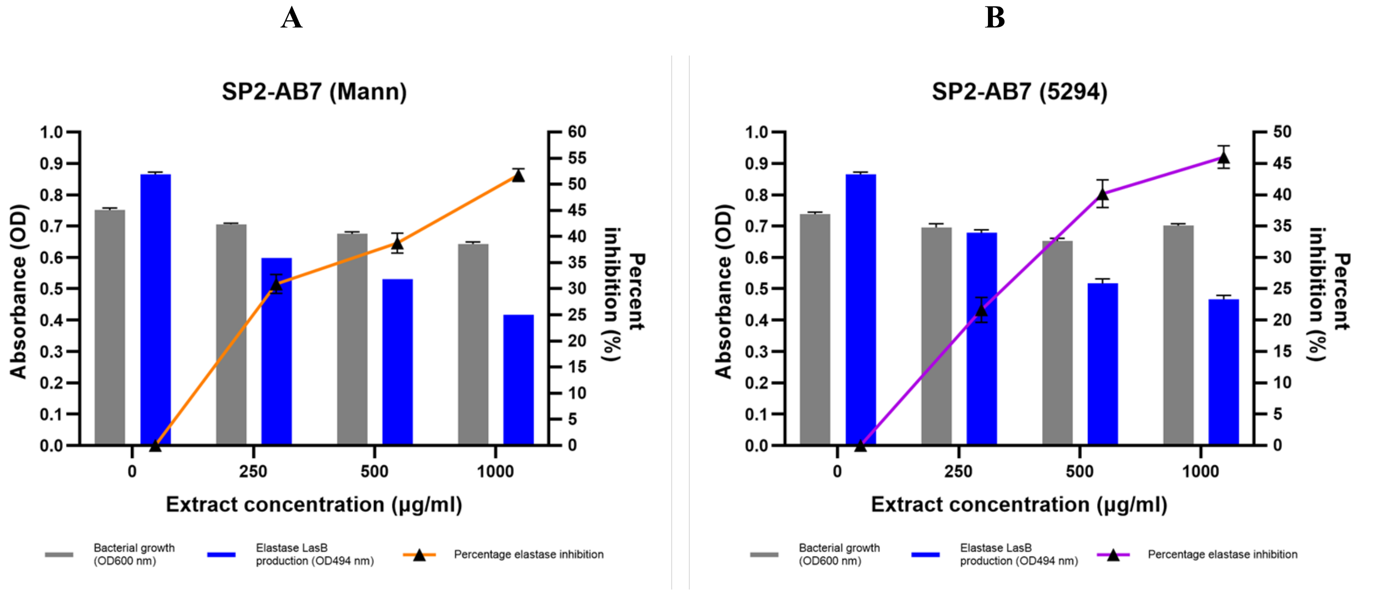

Figure S4.4. Inhibition of elastase LasB using sponge-associated bacterial extracts**.** Effect of *Bacillus mobilis* SP2-AB7 extracts on *Pseudomonas aeruginosa* growth and elastase production. (**A**) SP2-AB7 (Mann) and (**B**) SP2-AB7 (5294). Data reflects average results ±SD following experiments in triplicate on three separate occasions. Growth inhibition for both extracts was ≤40%.

A **B**

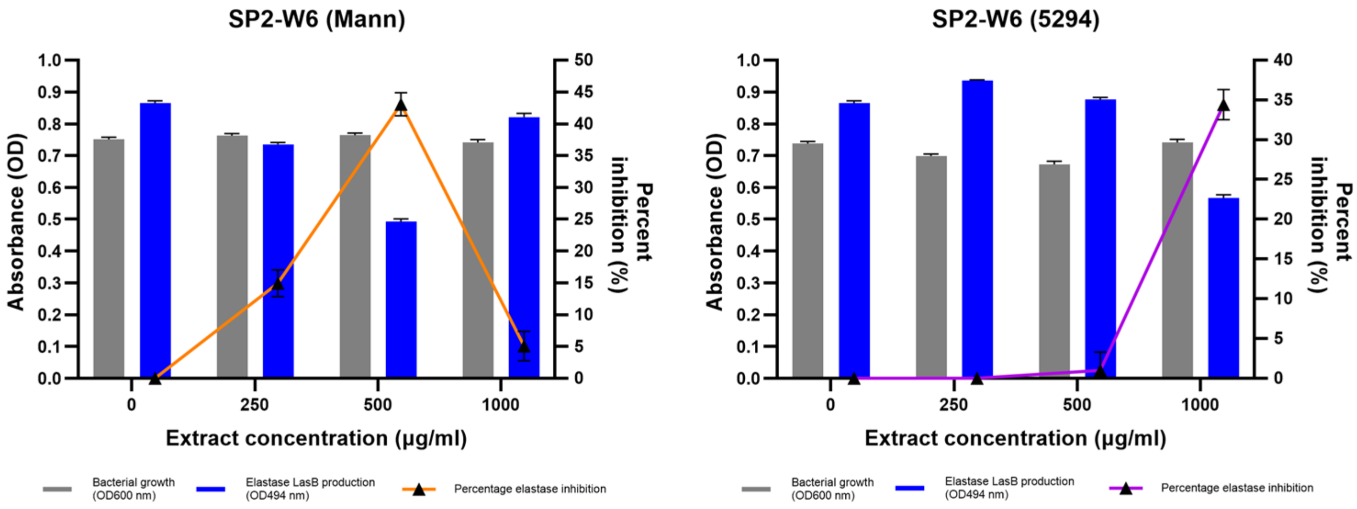

Figure S4.5. Inhibition of elastase LasB using sponge-associated bacterial extracts**.** Effect of *Bacillus pumilus* SP2-W6 extracts on *Pseudomonas aeruginosa* growth and elastase production. (**A**) SP2-W6 (Mann) and (**B**) SP2-W6 (5294). Data reflects average results ±SD following experiments in triplicate on two or three separate occasions. Growth inhibition for both extracts was ≤40%.

A **B**

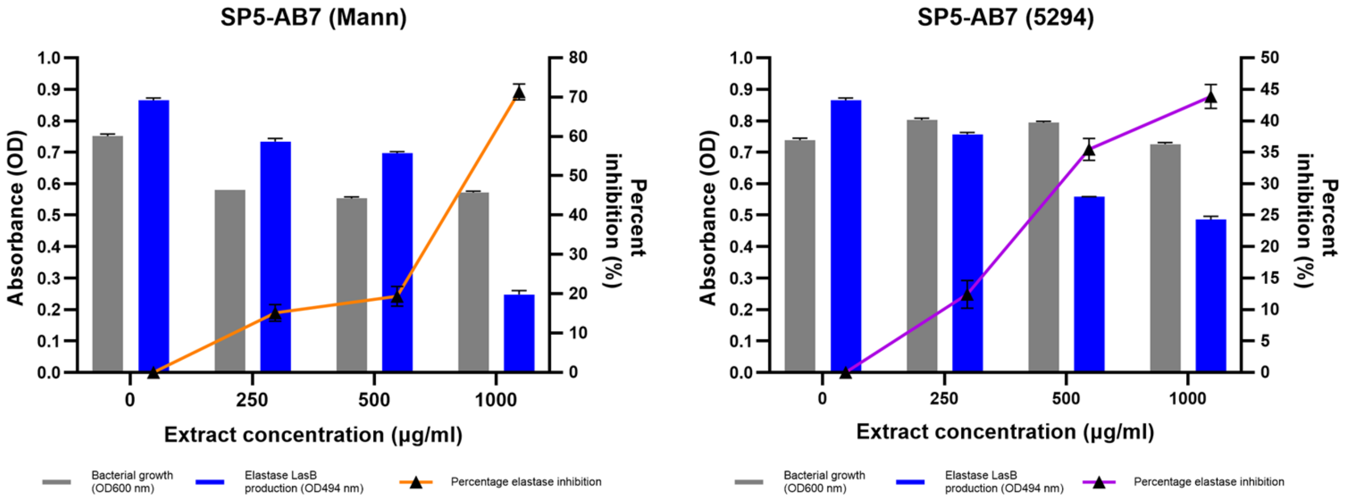

Figure S4.6. Inhibition of elastase LasB using sponge-associated bacterial extracts**.** Effect of *Bacillus wiedmannii* SP5-AB7 extracts on *Pseudomonas aeruginosa* growth and elastase production. (**A**) SP5-AB7 (Mann) and (**B**) SP5-AB7 (5294). Data reflects average results ±SD following experiments in triplicate on two or three separate occasions. Growth inhibition for both extracts was ≤40%.

Qualitative protease inhibition following treatment with sponge-associated *Bacillus* species extracts

Table S6. Qualitative protease inhibition following treatment with sponge-associated *Bacillus* species extracts. Zones of hydrolysis on casein agar plates following 24 h incubation at 37 °C are given with the calculated percentage inhibition for each extract treatment indicated.

Extract code	Untreated	250 µg/mL		500 µg/mL		1000 µg/mL	
	Zone of hydrolysis (mm) ± SD*	Zone of hydrolysis (mm) ± SD*	Protease inhibition (%)**	Zone of hydrolysis (mm) ± SD*	Protease inhibition (%)**	Zone of hydrolysis (mm) ± SD*	Protease inhibition (%)**
**SP-AB2 (Mannitol)**	25.14 ± 0.71	22.25 ± 1.06	11.50	23.50 ± 1.32	6.52	22.50 ± 1.29	10.50
**SP-AB2 (5294)**	25.14 ± 0.71	20.00 ± 0.00	20.45	20.75 ± 0.35	17.46	19.75 ± 0.35	21.44
**SP1-AB4 (Mannitol)**	25.14 ± 0.71	19.50 ± 0.71	22.43	19.00 ± 0.00	24.42	17.50 ± 0.71	30.39
**SP1-AB4 (5294)**	25.14 ± 0.71	21.00 ± 2.00	16.47	21.88 ± 1.93	12.99	21.58 ± 1.59	14.15
**SP2-AB7 (Mannitol)**	25.14 ± 0.71	20.13 ± 0.18	19.95	21.75 ± 0.35	13.48	20.50 ± 0.50	18.46
**SP2-AB7 (5294)**	25.14 ± 0.71	21.38 ± 0.18	14.98	21.88 ± 0.53	12.99	20.63 ± 0.18	17.96
**SP2-W6 (Mannitol)**	25.14 ± 0.71	21.00 ± 1.41	16.47	18.00 ± 0.71	28.40	19.00 ± 0.00	24.42
**SP2-W6 (5294)**	25.14 ± 0.71	19.50 ± 0.00	22.43	18.50 ± 0.71	26.41	19.38 ± 0.53	22.93
**SP5-AB7 (Mannitol)**	25.14 ± 0.71	17.38 ± 0.53	30.89	18.75 ± 0.35	25.42	19.75 ± 0.35	21.44
**SP5-AB7 (5294)**	25.14 ± 0.71	19.13 ± 0.18	23.93	19.13 ± 0.18	23.93	18.63 ± 0.53	25.91
**Cinnamaldehyde$**	25.14 ± 0.71	20.50 ± 0.71	18.46	18.00 ± 0.00	28.40	11.50 ± 0.71	54.26
**10% DMSO**	25.14 ± 0.71	23.50 ± 0.71	6.52	23.00 ± 1.41	8.51	23.50 ± 0.71	6.52

*Average of casein hydrolysis zones ± standard deviation.

**Protease activity is expressed based on the diameter of the hydrolysis zone. QS inhibitory compounds should reduce protease activity of *P. aeruginosa* ATCC 27853, and thus, in their presence, the hydrolysis zone should be of smaller dimensions than in the untreated control (<25.14 mm).

$Protease inhibition for cinnamaldehyde at 1000 µg/mL is due to *P. aeruginosa* cell death. All other extracts tested had a growth inhibitory effect of <40%.

Quantitative protease and growth inhibitions following treatment with sponge-associated *Bacillus* species extracts

A B

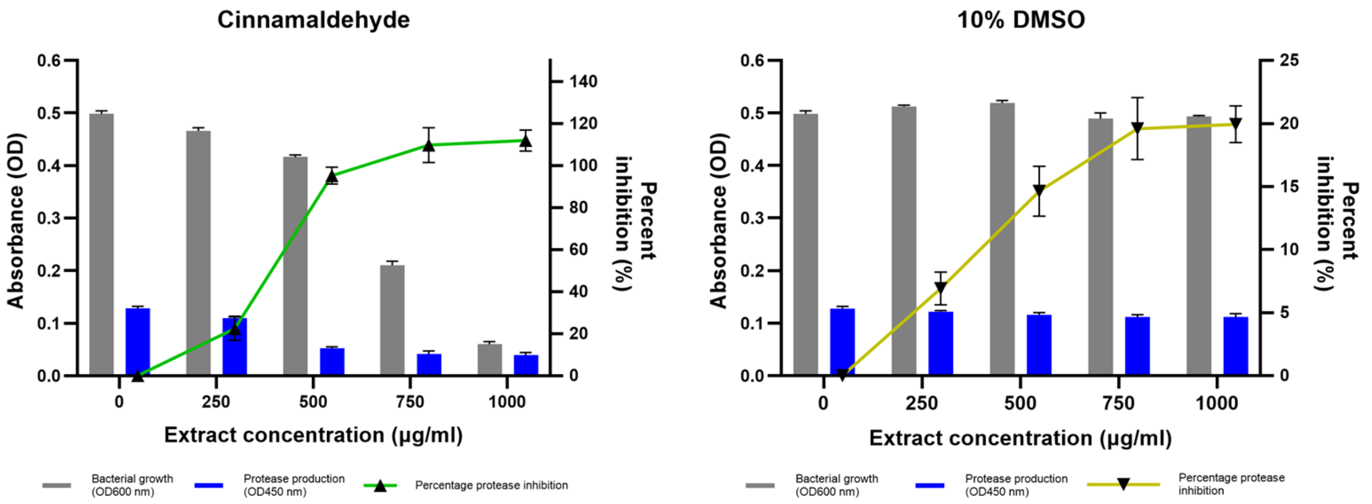

Figure S5.1. Inhibition of protease using cinnamaldehyde and 10% DMSO**.** Positive control cinnamaldehyde (**A**) resulted in ≥40% *Pseudomonas aeruginosa* growth inhibition from 750 µg/mL, contributing to the decrease in protease production and is not indicative of QSI. Solvent 10% DMSO (**B**) had a negligible effect on cell growth, however a significant effect on protease production ($p < 0.05$) which needs to be taken into consideration. Data reflects average results ±SD following experiments in triplicate on two or three separate occasions.

A B

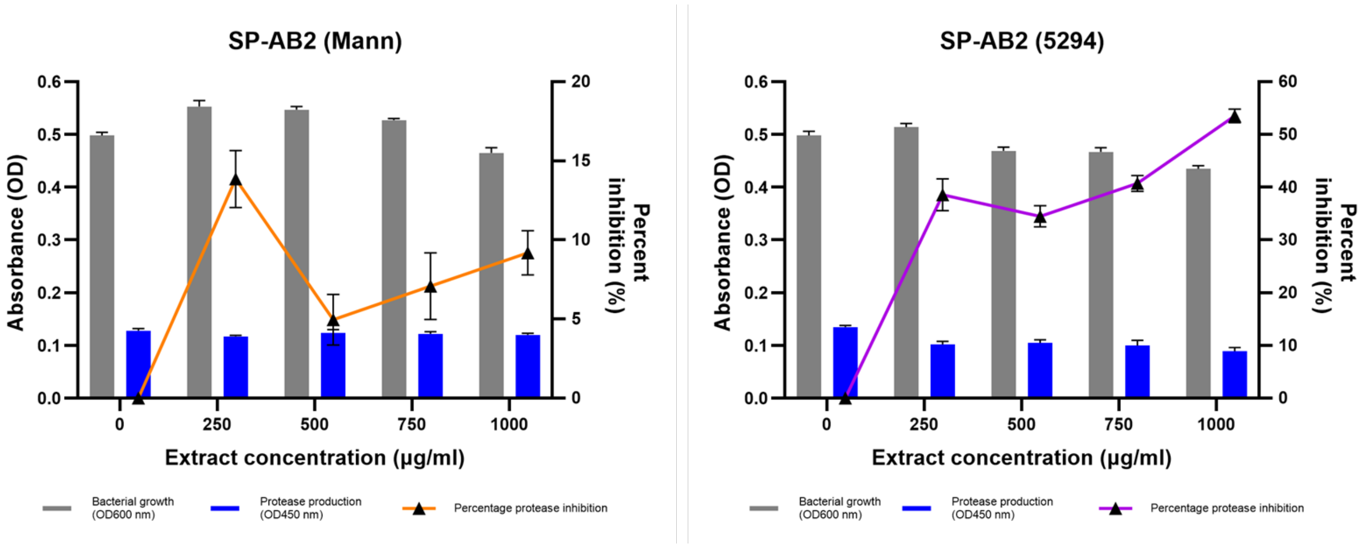

Figure S5.2. Inhibition of protease using sponge-associated bacterial extracts**.** Effect of *Bacillus thuringiensis* SP-AB2 extracts on *Pseudomonas aeruginosa* growth and protease production. (**A**) SP-AB2 (Mann) and (**B**) SP-AB2 (5294). Data reflects average results ±SD following experiments in triplicate on two or three separate occasions. Growth inhibition for both extracts was ≤40%.

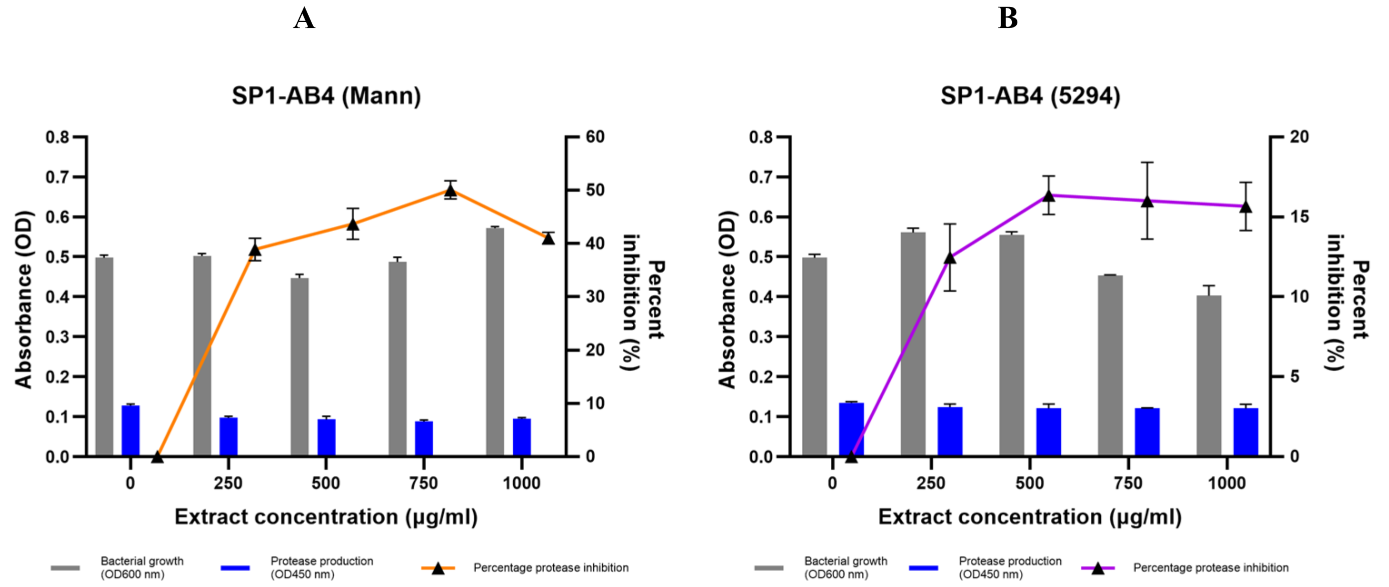

Figure S5.3. Inhibition of protease using sponge-associated bacterial extracts**.** Effect of *Bacillus cereus* SP1-AB4 extracts on *Pseudomonas aeruginosa* growth and protease production. (**A**) SP1-AB4 (Mann) and (**B**) SP1-AB4 (5294). Data reflects average results ±SD following experiments in triplicate on two or three separate occasions. Growth inhibition for both extracts was ≤40%.

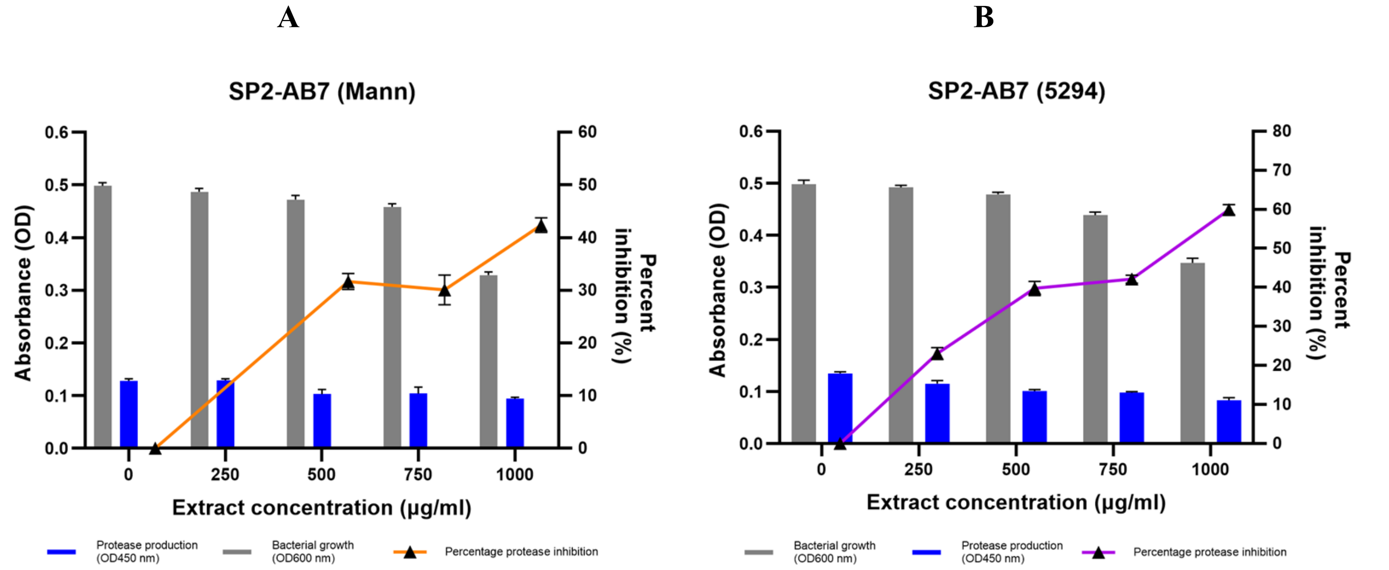

Figure S5.4. Inhibition of protease using sponge-associated bacterial extracts**.** Effect of *Bacillus mobilis* SP2-AB7 extracts on *Pseudomonas aeruginosa* growth and protease production. (**A**) SP2-AB7 (Mann) and (**B**) SP2-AB7 (5294). Data reflects average results ±SD following experiments in triplicate on two or three separate occasions. Growth inhibition for both extracts was ≤40%.

A B

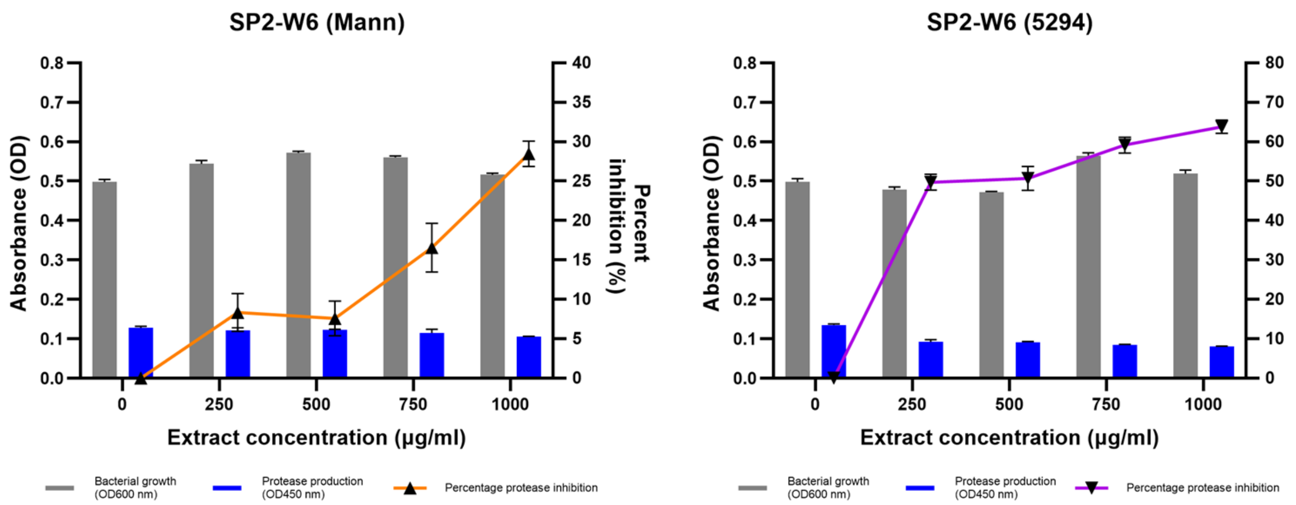

Figure S5.5. Inhibition of protease using sponge-associated bacterial extracts**.** Effect of *Bacillus pumilus* SP2-W6 extracts on *Pseudomonas aeruginosa* growth and protease production. (**A**) SP2-W6 (Mann) and (**B**) SP2-W6 (5294). Data reflects average results ±SD following experiments in triplicate on two or three separate occasions. Growth inhibition for both extracts was ≤40%.

A B

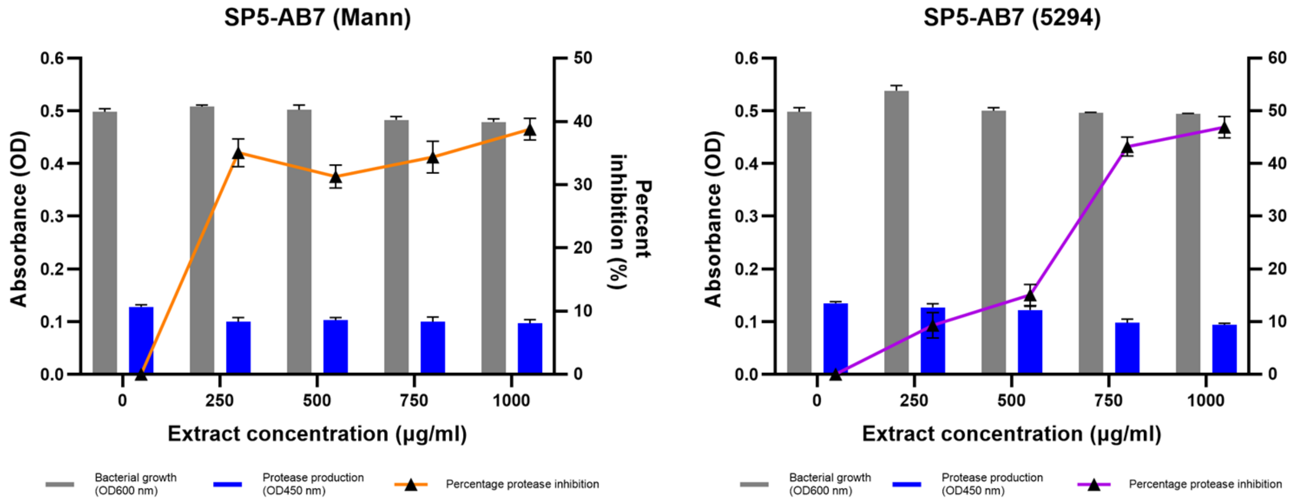

Figure S5.6. Inhibition of protease using sponge-associated bacterial extracts**.** Effect of *Bacillus wiedmannii* SP5-AB7 extracts on *Pseudomonas aeruginosa* growth and protease production. (**A**) SP5-AB7 (Mann) and (**B**) SP5-AB7 (5294). Data reflects average results ±SD following experiments in triplicate on two or three separate occasions. Growth inhibition for both extracts was ≤40%.

Qualitative rhamnolipid inhibitions following treatment with sponge-associated *Bacillus* species extracts

Table S7. Qualitative rhamnolipid inhibition following treatment with sponge-associated *Bacillus* species extracts. Zones of rhamnolipid production on CTAB agar plates following 24 h incubation at 37 °C are given with percentage inhibition for each extract treatment indicated.

	Untreated	250 µg/mL		500 µg/mL		1000 µg/mL	
**Extract code**	**Rhamnolipid production (mm)** ± SD***	**Rhamnolipid production (mm)** ± SD***	**Rhamnolipid inhibition (%)**	**Rhamnolipid production (mm)** ± SD***	**Rhamnolipid inhibition (%)**	**Rhamnolipid production (mm)** ± SD***	**Rhamnolipid inhibition (%)**
**SP-AB2 (Mannitol)**	20.71 ± 0.35	11.50 ± 4.24	44.47	10.00 ± 1.41	51.71	9.38 ± 1.24	54.73
**SP-AB2 (5294)**	20.71 ± 0.35	10.13 ± 2.65	51.11	10.25 ± 5.30	50.51	12.50 ± 2.12	39.64
**SP1-AB4 (Mannitol)**	20.71 ± 0.35	16.13 ± 0.88	22.14	16.50 ± 0.71	20.33	17.25 ± 0.35	16.71
**SP1-AB4 (5294)**	20.71 ± 0.35	12.88 ± 4.77	37.83	13.13 ± 3.71	36.62	12.00 ± 3.54	42.06
**SP2-AB7 (Mannitol)**	20.71 ± 0.35	12.00 ± 5.66	42.06	12.38 ± 4.42	40.25	11.00 ± 4.95	46.89
**SP2-AB7 (5294)**	20.71 ± 0.35	12.00 ± 4.95	42.06	12.50 ± 4.24	39.64	12.50 ± 4.95	39.64
**SP2-W6 (Mannitol)**	20.71 ± 0.35	18.50 ± 2.12	10.67	19.75 ± 0.35	4.64	18.75 ± 1.77	9.46
**SP2-W6 (5294)**	20.71 ± 0.35	15.50 ± 5.66	25.16	18.00 ± 3.54	13.09	18.50 ± 1.41	10.67
**SP5-AB7 (Mannitol)**	20.71 ± 0.35	10.75 ± 2.47	48.09	6.50 ± 1.41	68.61	6.75 ± 2.47	67.41
**SP5-AB7 (5294)**	20.71 ± 0.35	12.00 ± 2.83	42.06	10.00 ± 1.41	51.71	12.00 ± 1.41	42.06
**Cinnamaldehyde$**	20.71 ± 0.35	19.25 ± 0.35	7.05	13.50 ± 0.71	34.81	9.50 ± 0.71	54.13
**10% DMSO**	20.71 ± 0.35	20.75 ± 0.35	-0.19#	20.75 ± 0.35	-0.19#	21.00 ± 0.00	-1.40#

*All readings of rhamnolipid production are given ± standard deviation.

**Rhamnolipid production is expressed based on the diameter of the rhamnolipid hydrolysis zone – bacterial growth diameter. QS inhibitory compounds should reduce rhamnolipid production of *P. aeruginosa* ATCC 27853, and thus, in their presence, the hydrolysis zone should be of smaller dimensions than in the untreated control (<20.71 mm).

$Rhamnolipid inhibition for cinnamaldehyde at 1000 µg/mL is due to *P. aeruginosa* cell death. All other extracts tested had a growth inhibitory effect of <40% at all concentrations tested.

Negative values are indicative of an increase in rhamnolipid production at this concentration.

Quantitative rhamnolipid and growth inhibitions following treatment with sponge-associated *Bacillus* species extracts

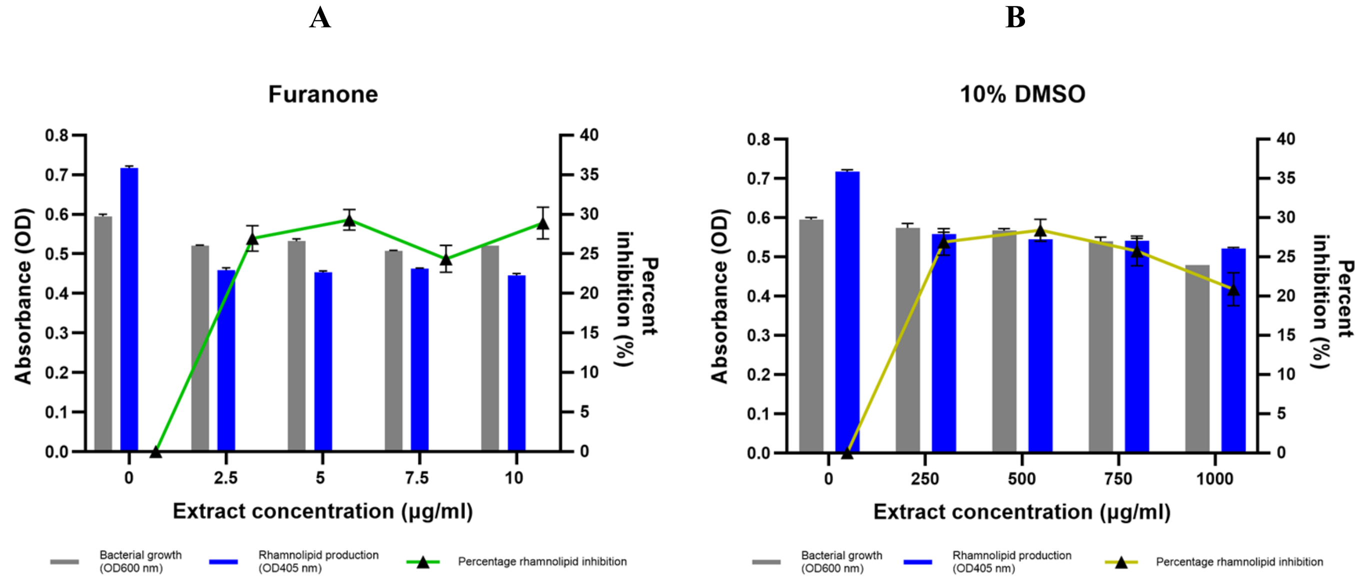

Figure S6.1. Inhibition of rhamnolipids using furanone and 10% DMSO**.** Positive control furanone (**A**) did not result in *Pseudomonas aeruginosa* cell death and inhibited rhamnolipid production insignificantly ($p > 0.05$) in a concentration independent manner. Solvent 10% DMSO (**B**) had a negligible effect on cell growth, as well as an insignificant effect on rhamnolipid production ($p > 0.05$). Data reflects average results ±SD following experiments in triplicate on three separate occasions.

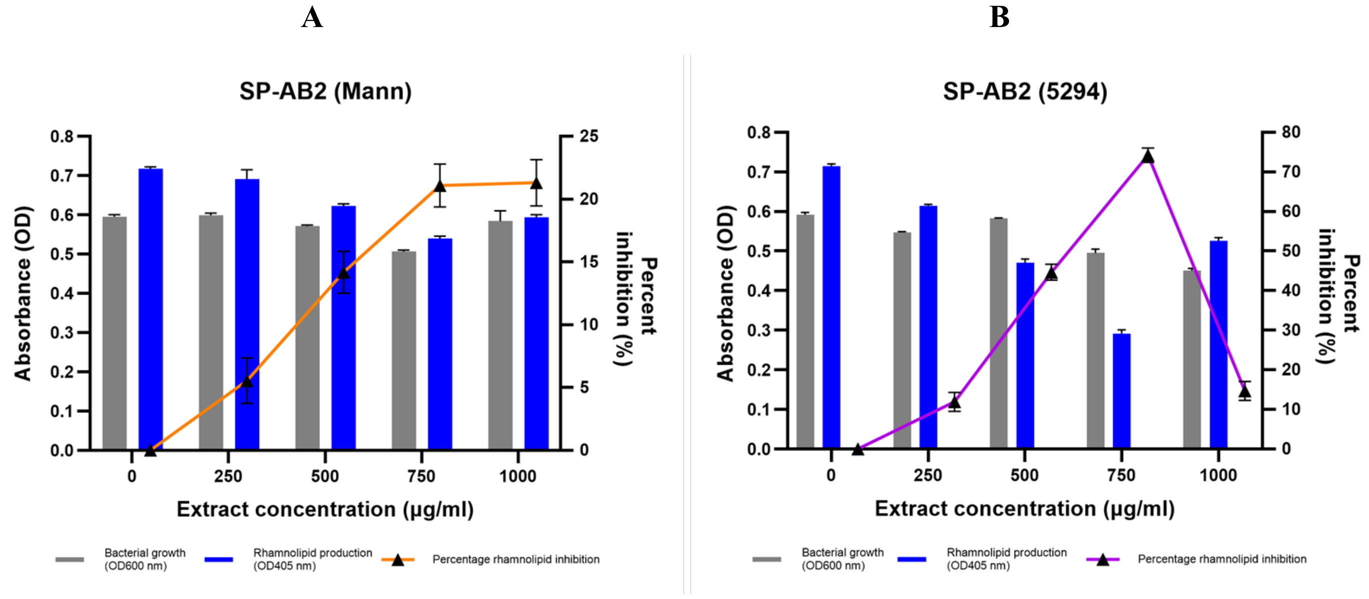

Figure S6.2. Inhibition of rhamnolipids using sponge-associated bacterial extracts**.** Effect of *Bacillus thuringiensis* SP-AB2 extracts on *Pseudomonas aeruginosa* growth and rhamnolipid production. (**A**) SP-AB2 (Mann) and (**B**) SP-AB2 (5294). Data reflects average results ±SD following experiments in triplicate on three separate occasions. Growth inhibition for both extracts was ≤40%.

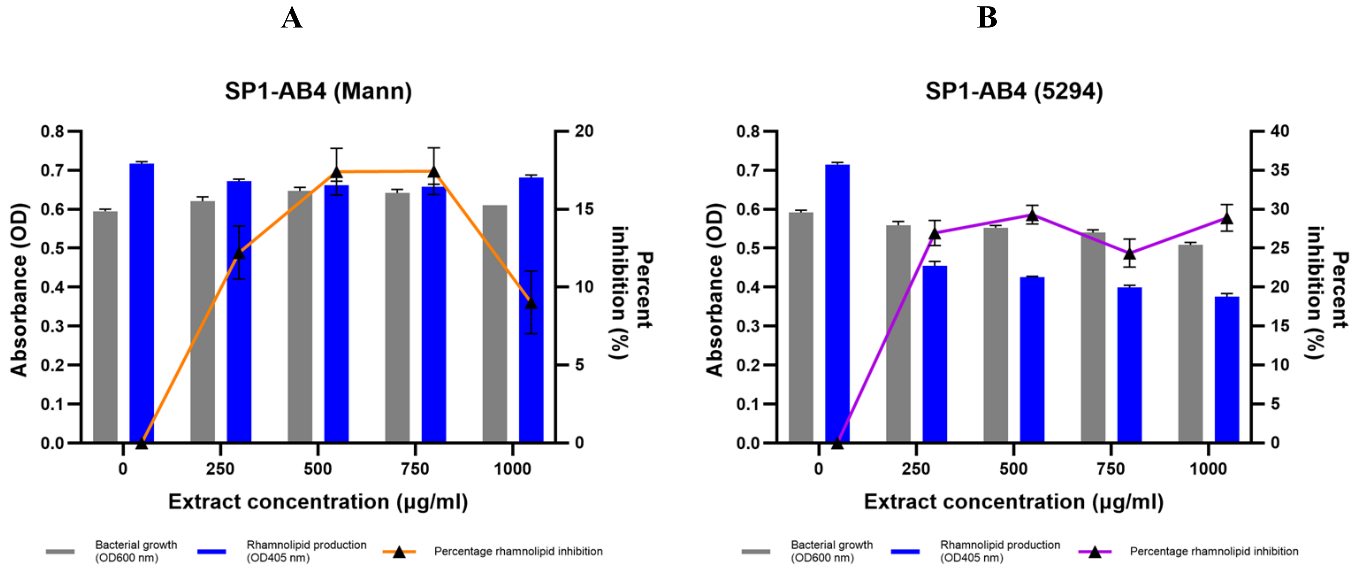

Figure S6.3. Inhibition of rhamnolipids using sponge-associated bacterial extracts**.** Effect of *Bacillus cereus* SP1-AB4 extracts on *Pseudomonas aeruginosa* growth and rhamnolipid production. (**A**) SP1-AB4 (Mann) and (**B**) SP1-AB4 (5294). Data reflects average results ±SD following experiments in triplicate on three separate occasions. Growth inhibition for both extracts was ≤40%.

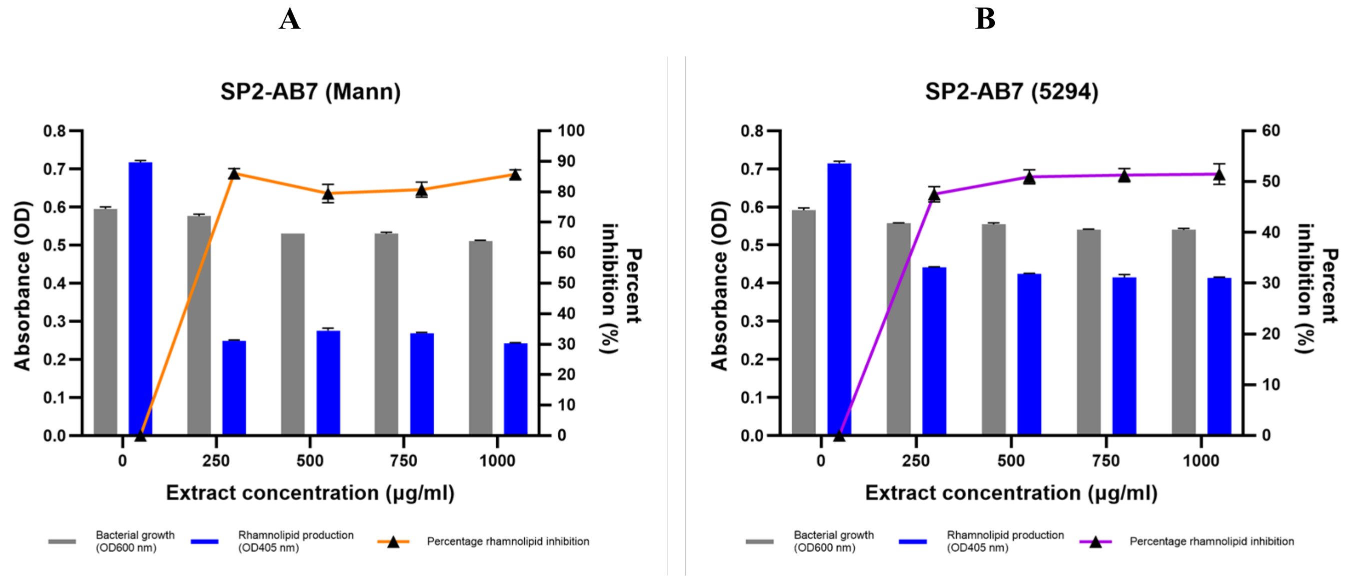

Figure S6.4. Inhibition of rhamnolipids using sponge-associated bacterial extracts**.** Effect of *Bacillus mobilis* SP2-AB7 extracts on *Pseudomonas aeruginosa* growth and rhamnolipid production. (**A**) SP2-AB7 (Mann) and (**B**) SP2-AB7 (5294). Data reflects average results ±SD following experiments in triplicate on three separate occasions. Growth inhibition for both extracts was ≤40%.

A **B**

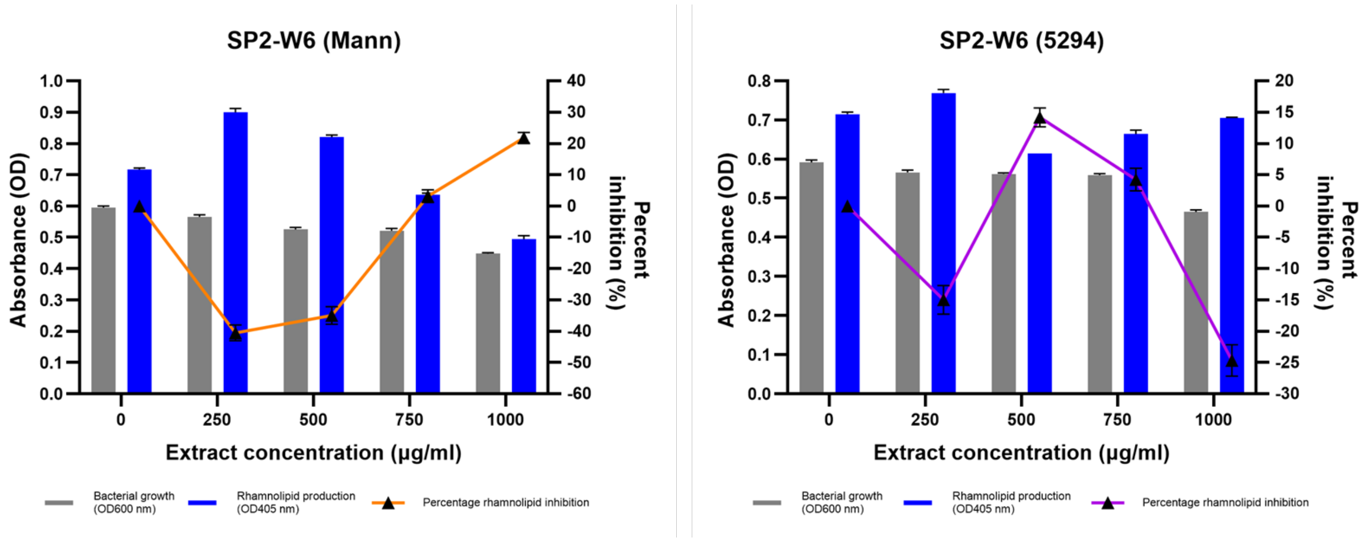

Figure S6.5. Inhibition of rhamnolipids using sponge-associated bacterial extracts**.** Effect of *Bacillus pumilus* SP2-W6 extracts on *Pseudomonas aeruginosa* growth and rhamnolipid production. (**A**) SP2-W6 (Mann) and (**B**) SP2-W6 (5294). Data reflects average results ±SD following experiments in triplicate on three separate occasions. Growth inhibition for both extracts was ≤40%.

A **B**

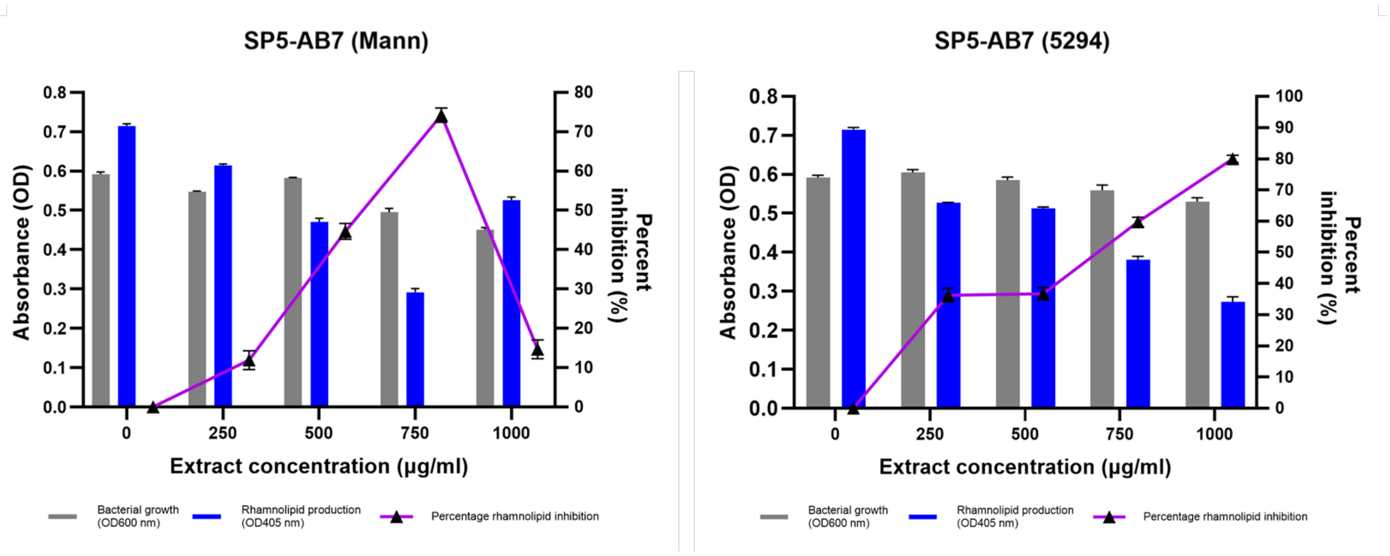

Figure S6.6. Inhibition of rhamnolipids using sponge-associated bacterial extracts**.** Effect of *Bacillus wiedmannii* SP5-AB7 extracts on *Pseudomonas aeruginosa* growth and rhamnolipid production. (**A**) SP5-AB7 (Mann) and (**B**) SP5-AB7 (5294). Data reflects average results ±SD following experiments in triplicate on three separate occasions. Growth inhibition for both extracts was ≤40%.

Inhibition of *P. aeruginosa* motility following treatment with sponge-associated *Bacillus* species extracts

Table S8. Inhibition of swimming motility following treatment with sponge-associated *Bacillus* species extracts. Readings were taken every 24 h following incubation at 37 °C for 72 h. Readings taken at 72 h are reflected here as they were considered to reflect sustained QSI activity.

Extract code	Untreated	250 µg/mL		500 µg/mL		1000 µg/mL	
	Zone of swimming (mm) ± SD*	Zone of swimming (mm) ± SD*	Swimming inhibition (%)	Zone of swimming (mm) ± SD*	Swimming inhibition (%)	Zone of swimming (mm) ± SD*	Swimming inhibition (%)
**SP-AB2 (Mannitol)**	74.60 ± 1.00	53.25 ± 4.75	20.52	56.50 ± 0.00	15.67	62.50 ± 0.50	6.72
**SP-AB2 (5294)**	74.60 ± 1.00	55.50 ± 0.50	34.71	57.75 ± 0.75	32.06	53.25 ± 0.25	37.35
**SP1-AB4 (Mannitol)**	74.60 ± 1.00	54.00 ± 3.50	19.40	53.50 ± 1.50	20.15	54.75 ± 0.25	18.28
**SP1-AB4 (5294)**	74.60 ± 1.00	50.75 ± 2.75	40.29	61.50 ± 1.00	27.65	52.75 ± 2.75	37.94
**SP2-AB7 (Mannitol)**	74.60 ± 1.00	54.00 ± 0.00	36.47	60.50 ± 0.50	28.82	61.00 ± 1.00	28.24
**SP2-AB7 (5294)**	74.60 ± 1.00	37.50 ± 0.50	55.88	48.00 ± 0.00	43.53	42.00 ± 1.00	50.59
**SP2-W6 (Mannitol)**	74.60 ± 1.00	55.50 ± 3.00	17.16	40.50 ± 0.50	39.55	43.50 ± 0.00	35.07
**SP2-W6 (5294)**	74.60 ± 1.00	46.00 ± 4.50	31.34	50.50 ± 0.00	24.63	54.00 ± 0.00	19.40
**SP5-AB7 (Mannitol)**	74.60 ± 1.00	57.00 ± 2.50	32.94	61.25 ± 3.75	27.94	65.00 ±0.00	23.53
**SP5-AB7 (5294)**	74.60 ± 1.00	58.25 ± 0.25	13.06	57.00 ± 0.00	14.93	47.00 ± 1.00	29.85
**Cinnamaldehyde$**	74.60 ± 1.00	37.00 ± 2.00	50.40	33.50 ± 1.50	55.09	41.50 ± 1.50	44.37
**10% DMSO**	74.60 ± 1.00	61.25 ± 5.25	17.90	60.50 ±2.00	18.90	59.00 ± 0.00	20.91

*All readings in swimming inhibition are given ± standard deviation.
$Swimming inhibition for cinnamaldehyde at 1000 µg/mL was the result of *P. aeruginosa* cell death. All other extracts tested had a growth inhibitory effect of <40% at all concentrations tested.

Table S9. Inhibition of swarming motility following treatment with sponge-associated *Bacillus* species extracts. Readings were taken every 24 h following incubation at 37 °C for 72 h. Readings taken at 72 h are reflected here as they were considered to reflect sustained QSI activity.

Extract code	Untreated	250 µg/mL		500 µg/mL		1000 µg/mL	
	Zone of swarming (mm) ± SD*	Zone of swarming (mm) ± SD*	Swarming inhibition (%)	Zone of swarming (mm) ± SD*	Swarming inhibition (%)	Zone of swarming (mm) ± SD*	Swarming inhibition (%)
**SP-AB2 (Mannitol)**	39.50 ± 0.50	32.00 ± 8.00	18.99	36.50 ± 0.00	7.59	34.25 ± 3.75	13.29
**SP-AB2 (5294)**	39.50 ± 0.50	27.50 ± 8.50	30.38	21.25 ± 5.75	46.20	22.75 ± 4.75	42.41
**SP1-AB4 (Mannitol)**	39.50 ± 0.50	28.50 ± 10.50	27.85	27.00 ± 10.50	31.65	37.25 ± 1.75	5.70
**SP1-AB4 (5294)**	39.50 ± 0.50	17.00 ± 0.00	56.96	17.50 ± 0.00	55.70	19.00 ± 0.00	51.90
**SP2-AB7 (Mannitol)**	39.50 ± 0.50	35.25 ± 1.75	10.76	16.00 ± 0.00	59.49	36.00 ± 0.00	8.86
**SP2-AB7 (5294)**	39.50 ± 0.50	25.00 ± 0.00	36.71	17.50 ± 1.50	55.70	28.00 ± 0.00	29.11
**SP2-W6 (Mannitol)**	39.50 ± 0.50	32.00 ± 2.00	18.99	40.00 ± 0.00	-1.27#	35.00 ± 0.00	11.39
**SP2-W6 (5294)**	39.50 ± 0.50	46.75 ± 22.75	-18.35#	34.50 ± 1.00	12.66	21.50 ± 4.00	45.57
**SP5-AB7 (Mannitol)**	39.50 ± 0.50	46.50 ± 4.00	-17.72#	37.50 ± 0.00	5.06	28.00 ± 0.00	29.11
**SP5-AB7 (5294)**	39.50 ± 0.50	29.75 ± 0.25	24.68	31.50 ± 0.00	20.25	30.00 ± 4.00	24.05
**Cinnamaldehyde$**	39.50 ± 0.50	22.50 ± 2.50	43.04	34.50 ± 3.50	12.66	42.75 ± 2.25	-8.23#
**10% DMSO**	39.50 ± 0.50	62.50 ± 2.50	-58.23#	72.50 ± 2.50	-83.54#	59.00 ± 3.00	-49.37#

*All readings in swarming inhibition are given ± standard deviation.
$Swarming inhibition for cinnamaldehyde at 1000 µg/mL was the result of *P. aeruginosa* cell death. All other extracts tested had a growth inhibitory effect of <40% at all concentrations tested.
#Negative values are indicative of an increase in swarming motility at this concentration.

Biofilm and growth inhibition following treatment with sponge-associated *Bacillus* species extracts

A

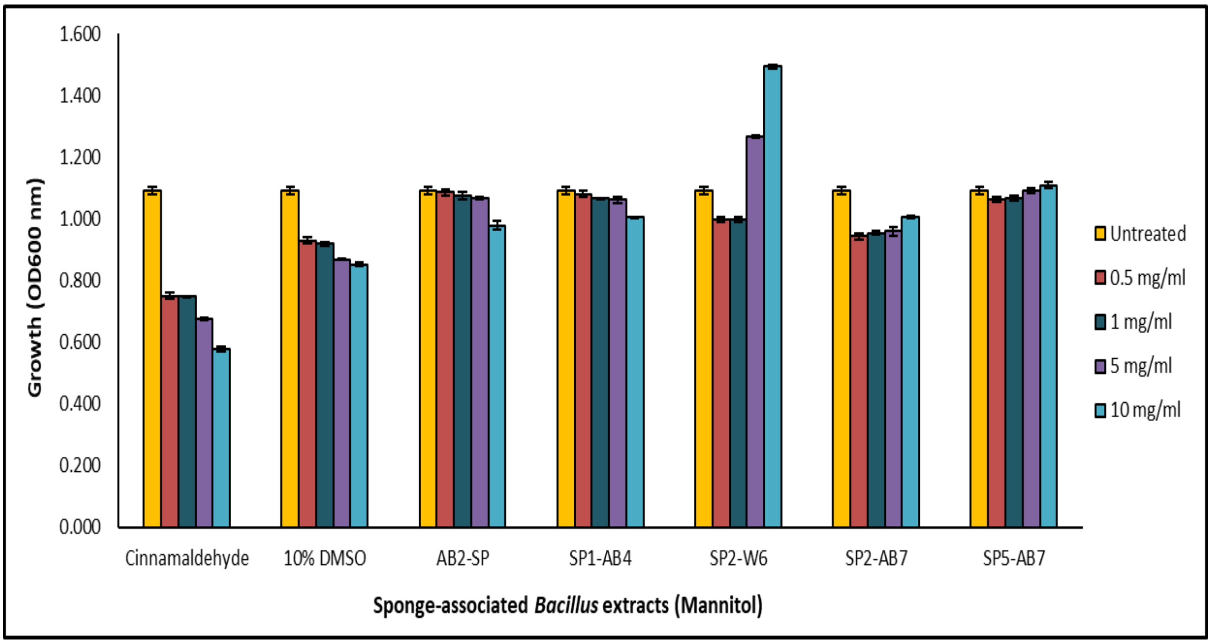

B

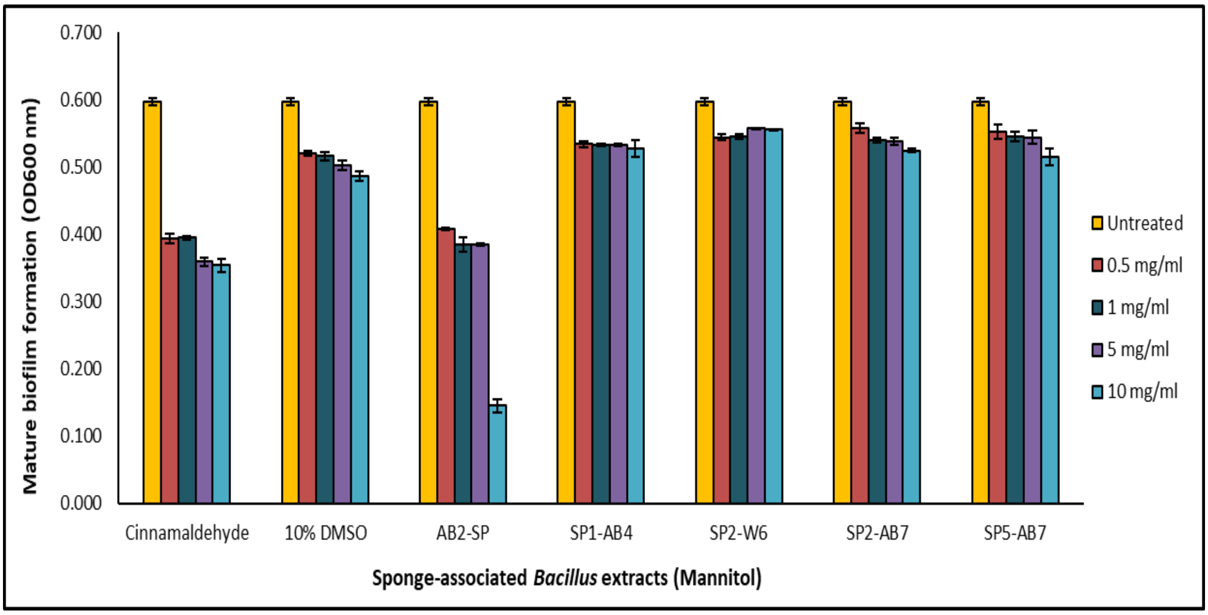

Figure S7. Effect of crude sponge-associated *Bacillus* species medium Mannitol extracts on *Pseudomonas aeruginosa* ATCC 27583 **(A)** bacterial growth and **(B)** mature biofilm as quantified by crystal violet staining in a microtiter plate assay. Cinnamaldehyde served as the positive control while volumes of 10% DMSO equivalent to those of the extracts tested as solvent control. Data represents the mean values of three replicates. Bars represent $OD_{600\ nm}$ values at varying concentrations.

A

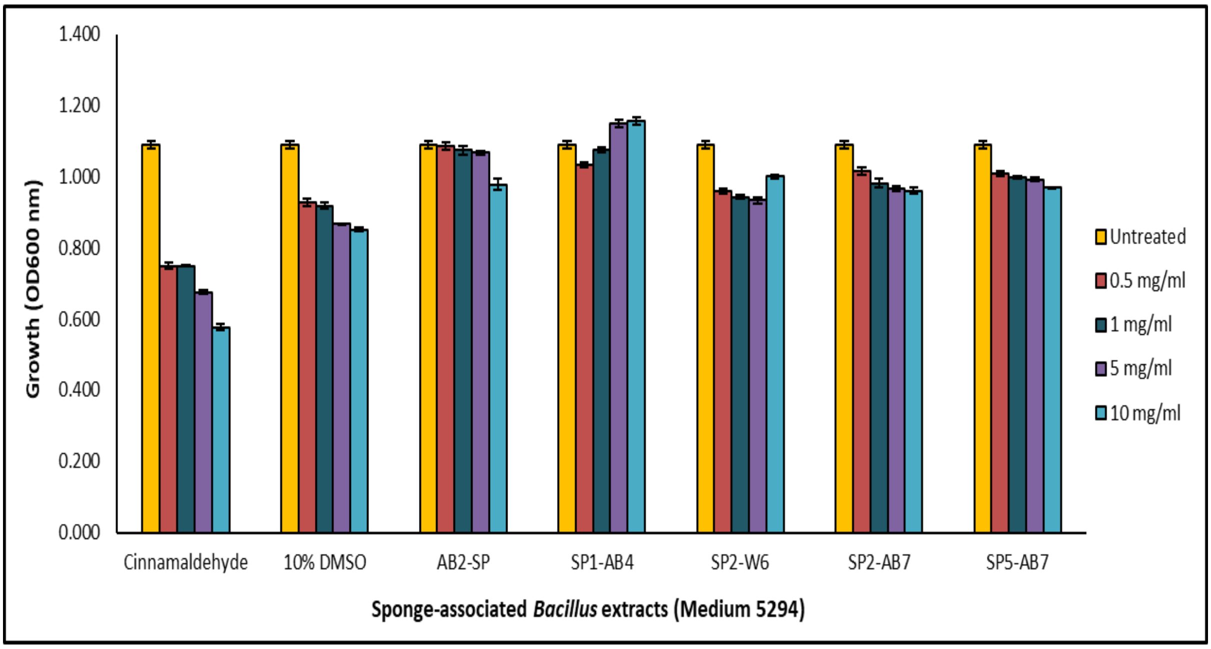

B

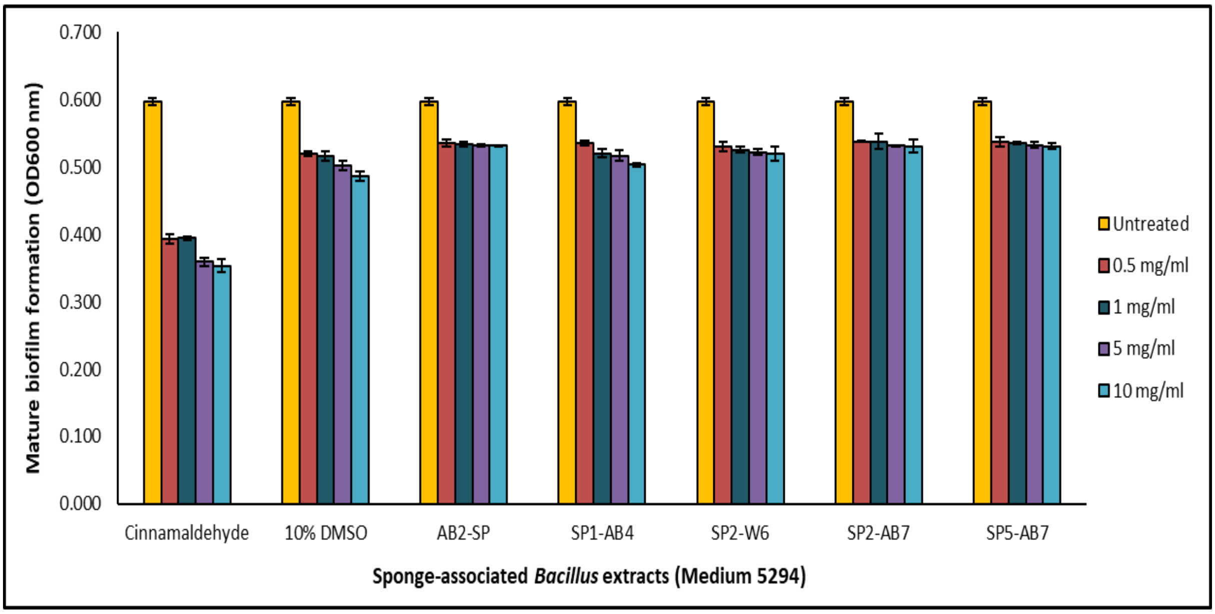

Figure S8. Effect of crude sponge-associated *Bacillus* species medium 5294 extracts on *Pseudomonas aeruginosa* ATCC 27583 **(A)** bacterial growth and **(B)** mature biofilm as quantified by crystal violet staining in a microtiter plate assay. Cinnamaldehyde served as the positive control while volumes of 10% DMSO equivalent to those of the extracts tested as solvent control. Data represents the mean values of three replicates. Bars represent $OD_{600\ nm}$ values at varying concentrations.

Table S10. Summary of the highest observed inhibition of virulence phenotypes regulated by QS in *Pseudomonas aeruginosa* ATCC 27853 by sponge-associated *Bacillus* species isolates obtained at varying treatment concentrations. Red colour = ≤ 20% inhibition of the virulence phenotype, Yellow colour = inhibition of the virulence phenotype ≤ 50%, and Green colour: inhibition of the virulence phenotype > 50%.

Extract	Pyocyanin	Pyoverdine	Elastase	Protease	Rhamnolipid	Swimming	Swarming	IA	MB
**SP-AB2 (Mann)**	32.63	38.48	26.04	9.19	21.30	20.52	18.99	88.77 (Killing)	86.28
**SP-AB2 (5294)**	36.21	26.89	2.35	53.51	74.22	37.35	46.20	40.96	12.33
**SP1-AB4 (Mann)**	35.57	31.43	50.32	50.07	17.44	20.15	31.65	73.58	13.26
**SP1-AB4 (5294)**	32.37	31.33	25.16	16.35	56.81	40.29	56.96	69.39	17.82
**SP2-AB7 (Mann)**	31.54	21.45	51.76	42.36	86.03	36.47	59.49	8.32	13.81
**SP2-AB7 (5294)**	19.83	30.92	46.01	59.93	51.49	55.88	55.70	4.35	12.55
**SP2-W6 (Mann)**	16.67	27.91	43.08	28.47	21.80	39.55	18.99	19.39	10.06
**SP2-W6 (5294)**	36.76	7.55	34.41	63.81	14.17	31.34	45.57	41.84	14.74
**SP5-AB7 (Mann)**	39.36	8.72	71.32	38.77	93.88	32.94	29.11	66.51	15.71
**SP5-AB7 (5294)**	30.97	34.86	43.86	46.90	79.96	29.85	24.68	19.54	12.54

Table S11. Summary of notable compounds identified *via* GC-MS together with mechanism of action, *Pseudomonas aeruginosa* QS system/s, targeted and effect of phenotype.

Isolate (Medium)	Key Compounds	Known/Proposed Mechanism	Targeted QS System	Phenotypes Inhibited
**SP2-AB7 (5294)**	1,2-benzenedicarboxylic acid (54.02%), n-hexadecanoic acid (5.95%)	LasR/PqsR binding	Las, PQS	Biofilm, Elastase, Pyocyanin
**SP5-AB7 (Mann)**	Benzeneacetic acid, cyclo(Leu-Pro)	Receptor antagonism, signal disruption	Rhl	Rhamnolipid, Pyocyanin
**SP2-AB7 (Mann)**	Malic acid (20.25%), cyclo(Leu-Pro) (5.75%)	Synergistic biofilm inhibition	Rhl	Rhamnolipid, Pyoverdine
**SP1-AB4 (Mann)**	Eicosane (18.27%), Heinecosane (5.11%)	Membrane interaction, anti-adhesion	Las	Initial Adhesion
